# Synthesis of First Copper Metal Complex of C=C Extended Curcuminoid Analogue: Structure, β-Cyclodextrin Association, and Biological Properties

**DOI:** 10.3390/molecules30193943

**Published:** 2025-10-01

**Authors:** Rosario Tavera-Hernández, Rubén Sánchez-Obregón, Marco A. Obregón-Mendoza, Antonio Nieto-Camacho, María Teresa Ramírez-Apan, Leidys L. Pérez-González, Raúl G. Enríquez

**Affiliations:** Instituto de Química, Universidad Nacional Autónoma de México, Circuito Exterior, Ciudad Universitaria, Mexico City 04510, Mexico; rubens@unam.mx (R.S.-O.); camanico2015@yahoo.com (A.N.-C.); mtrapan@yahoo.com.mx (M.T.R.-A.); leidyslaura92@gmail.com (L.L.P.-G.)

**Keywords:** analogue curcuminoid, copper complex, single-crystal structure, π-elongated chain, β-cyclodextrin complex, α-glucosidase

## Abstract

The search for bioactive compounds against chronic diseases such as cancer and diabetes includes curcuminoids as promising scaffolds. Here, we report the synthesis of a family of curcuminoid analogue compounds with an extended unsaturated central chain, as follows: difluoroboron complex **1**, the enolised curcuminoid **2**, and its homoleptic copper complex **3**, in moderate to good yields (68–90%). Additionally, their β-cyclodextrin (BCD) association complexes, **4** and **5**, were prepared through a mechanochemical method and characterised by spectroscopic techniques. Complete ^1^H and ^13^C NMR assignments and NOESY correlations revealed unique solvent effects on the conformational disposition of compound 2, while the copper complex **3** displayed the highest extinction coefficient (1.20 × 10^5^ M^−1^·cm^−1^). Furthermore, the authentication of the polymorph of **1** and the new crystal structures of **2** and **3**, determined by single-crystal X-ray analysis, were highlighted. Although the copper complex **3** initially exhibited the lowest a-glucosidase inhibitory activity (IC_50_ > 100 µM), it showed a significant increase (IC_50_ = 36.27 µM) upon association with BCD, reaching values comparable to the free ligand (IC_50_ = 45.63 µM). Compounds **1**–**5** were non-toxic to healthy cells (COS-7), but compound **5** stands out as a promising candidate against this metabolic condition.

## 1. Introduction

The search for new compounds with biological activity points to the family of natural products, a primary source that provides unique structural scaffolds with exceptional chemical and biological properties, and curcumin stands out with particular relevance [1,2]. Curcumin, demethoxycurcumin, and bisdemethoxycurcumin are polyphenols present in the rhizome of *Curcuma longa* [3]. Currently, the extensive study of these curcuminoids is due to their various reported biological activities, including antioxidant [4], anticancer [5], anti-inflammatory [6,7], antimicrobial [8], antidiabetic [9], and neuroprotective effects against Alzheimer’s disease [10,11]. Structurally, curcuminoids are composed of two aromatic rings connected by an unsaturated seven-carbon chain containing a β-diketone group, which exists in equilibrium between keto and enol tautomeric forms.

The current molecular architecture features an extended conjugated system that enables the formation of complexes with metal ions, altering its optical properties and interactions with biological targets [12]. Research has shown that modifications to the conjugation within the structure of curcuminoids, alongside their chelation with transition metals, can improve both the optical and biological properties of these compounds [13,14,15].

On the other hand, copper is a transition metal, and its ionic form is of physiological relevance as an essential component of many metalloenzymes involved in redox reactions [16]. Copper complexes have attracted increasing interest due to their biocompatibility and promising biological potential [17,18], since light excitation of Cu (II) complexes in the red region forms reactive species inducing cytotoxic activity, which represents an important factor in the photodynamic mechanism [19]. In addition, the importance the π-extended ligand in the improvement of high absorption in the near-infrared region has been reported [20]. Several studies have reported the antitumor [21,22], antimicrobial [23], and antioxidant [24] properties of copper complexes, as well as their inhibitory activity toward enzymes such as α-glucosidase [25]. α-Glucosidase is an enzyme of carbohydrate degradation, and its inhibition leads to control of postprandial blood glucose levels, resulting in interest for patients undergoing type 2 diabetes treatment [26,27]. Curcumin and curcuminoids exhibit inhibitory ability against the α-glucosidase enzyme, and the development of synthetic analogues has led to compounds with an improved inhibitory potency [28,29,30].

Previous research has reported homoleptic copper complexes with curcumin, diacetylcurcumin, dimethoxycurcumin, diphenylcurcumin, dibenzylcurcumin, and dietoxycurcumin as ligands. These copper complexes exhibit predominantly a square-planar geometry, forming ML_2_ structures through coordination with the β-diketone group of curcuminoids. Their antioxidant and antiproliferative activities and imaging properties improved in comparison with the free ligand [15,24,31].

There are limited investigations related to the synthesis, characterisation, and evaluation of the biological activity of curcuminoid analogues with extended conjugation in the unsaturated central chain. These curcuminoid analogues are particularly noteworthy for their optical properties [14,32,33] and as potential photosensitisers [34,35].

Although the biological and optical potential of curcuminoids is significant, their low aqueous solubility, susceptibility to hydrolysis, and photodegradation limit practical biological evaluation and bioavailability. Among the strategies to improve solubility in aqueous media, the formation of complexes with cyclodextrin has proven particularly effective in improving solubility and chemical stability [36,37]. β-Cyclodextrin (BCD) is efficient at forming complexes and is the most common available cyclodextrin. Structurally, BCD is composed of seven α-1,4-linked D-glucopyranose units, resulting in a toroid structure with a cavity (6–6.5 Å) of lower polarity than the surface [38,39].

Considering the potential for exploring curcuminoids and their metal complexes, the present study aims to examine a family of analogue curcuminoids with an extended eleven-carbon unsaturated central chain, using cinnamaldehyde for synthetic convenience due to its favourable aldolic condensation reaction and the lack of functional groups in the aromatic fragment promoting efficient chelation at the β-diketone function.

The curcuminoid analogue, as well as its boron and copper complexes, were fully characterised by spectroscopic, spectrometric, and X-ray diffraction methods. Furthermore, we report the formulation of the analogue and its copper complex with BCD (compounds **4** and **5**), as well as their spectroscopic characterisation, evaluating the α-glucosidase and antiproliferative activity inhibition of these compounds.

## 2. Results

### 2.1. Synthesis

**Scheme 1:**
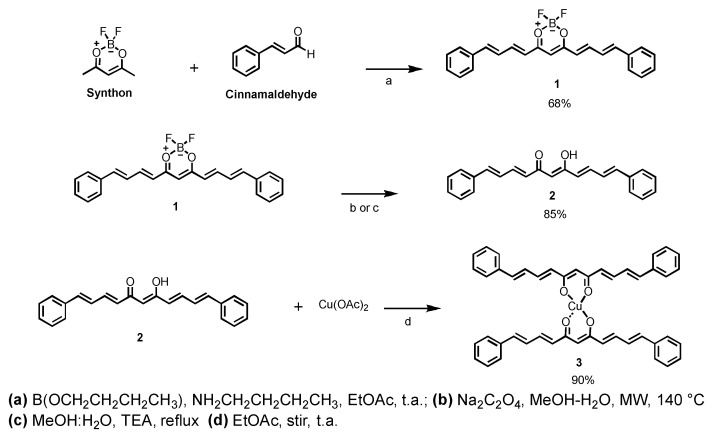
Reaction conditions in the synthesis of curcuminoid analogue (**2**) and its homoleptic copper complex (**3**).

The synthon was prepared in good yield (88%) using 2,4-pentanodione and THF-boron trifluoride complex. In this compound, the -BF_2_ group increases the acidity of methyl groups and prevents the Knovenagel reaction in the next step. Then, the condensation reaction between the synthon and cinnamaldehyde was conducted with tributyl borate and n-butylamine to produce the curcuminoid-BF_2_ analogue **1** as a dark purple solid in moderate yield (68%). The synthesis of ligand **2** was carried out following the reaction conditions previously reported [40], introducing minor modifications in the removal of the difluoroboron group. The cleavage of the diflouroboron group was carried out through reflux with triethylamine and methanol–DMSO–water as solvent (yield: 81%) or applying microwave irradiation using sodium oxalate in methanol–water as solvent at 140 °C for ten minutes (yield: 85%). Both methods have been reported as efficient to cleave the -BF_2_ group in the synthesis of curcuminoids [41,42]. Finally, compound **2** with the free β-diketone group and copper (II) acetate in ethyl acetate solution produced the homoleptic copper complex (**3**) with a 90% yield. Scheme 1 shows the reactions undertaken to obtain compounds **1**–**3**.

### 2.2. Nuclear Magnetic Resonance

**Figure 1:**
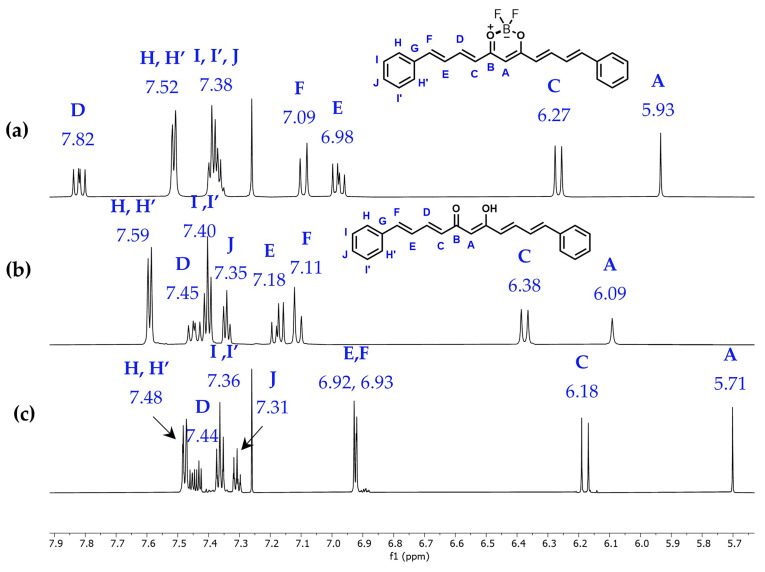
700 MHz ^1^H-NMR spectra of (**a**) curcuminoid-BF_2_ **1** in CDCl_3_; (**b**) curcuminoid analogue **2** in DMSO-d_6_; and (**c**) compound **2** in CDCl_3_.

Few reports document the synthesis of curcuminoid analogues (**2**). These reports provide limited information regarding their chemical shifts, with the assignments for protons and carbons largely absent. We examined the NMR spectra of **1** and **2** using 1D and 2D nuclear magnetic resonance experiments (Supplementary Material). Figure 1 presents the stacked ^1^H-NMR spectra of compounds **1** and **2**, alongside the hydrogen and carbon assignments in DMSO-d_6_ and CDCl_3_, as shown in Table 1.

The ^1^H NMR spectra of **1** and **2** show their own set of signals due to magnetic equivalence. Thus, the aromatic protons in **1** (CDCl_3_) appear as multiplets at δ7.38 and δ7.52 ppm, while in compound **2** (DMSO), they are multiplets at δ7.35, δ7.40, and δ7.59 ppm. Unsaturated protons H_D_ and H_E_ in **1** appear as a doublet of doublets at δ7.82 and δ6.98 ppm, while in **2**, they appear at δ7.45 and δ7.18 ppm, respectively. Coupling constants for H_D_ and H_E_ in both compounds are ca. 15 Hz due to the *trans* coupling of the unsaturated proton system. H_F_ and H_C_ hydrogens in **1** appear at δ7.09 and δ6.27 ppm with ca. *J* = 15.4 and 14.8, respectively. The corresponding set for **2** are doublets at δ7.11 and δ6.38 ppm with *J* = 15.6 and *J* = 15.1 Hz, respectively. The methine hydrogen appears as a singlet at δ5.93 and δ5.7 ppm in **1** and **2,** respectively. In addition, the ^1^H-NMR spectrum of **2** displays a broad signal at 16.06 ppm corresponding to -OH, which forms a hydrogen bond, indicating the keto-enol tautomer.

The ^1^H-NMR of compound **2** was also acquired in CDCl_3_, and its assignments are shown in Figure 1c and Table 1. In comparison with the spectrum in DMSO-d_6_, different multiplicities for H_D_, H_E_, and H_F_ were observed as a consequence of changes in chemical shifts. In addition, the NOESY spectrum showed interactions at greater distances between H_D_-H_A_ and H_H_-Hc when chloroform was used as solvent. The complete set of interactions is shown in Table S1.

### 2.3. Electronic Paramagnetic Resonance

The electron paramagnetic resonance spectrum of compound **3** (Figure S22) exhibited two sets of quartet hyperfine lines characteristic of copper (II) coordination complexes, indicating the coexistence of two species. The experimental values obtained directly from the spectrum are g‖ = 2.2819 and 2.3040, gꞱ = 2.0430 and 2.0544, while the A‖ values are 168.9 and 160.0 × 10^−4^ cm^−1^. In both cases, g‖ > gꞱ, suggesting an axial square-planar coordination geometry [43]. The calculated ratios *f* = g‖/A‖ are 135.1 and 144 cm, close to 150 cm, which is associated with a slight to moderate distortion from planar geometry [44].

### 2.4. Mass Spectrometry

The mass spectrum compound (**1**) showed a peak at *m/z* 411.6 [M + Cl]^−^, which is in agreement with the mass expected for this compound. In the case of ligand (**2**), a peak at *m/z* 329.9 corresponds to a [M + H]^+^ molecular ion, and the mass spectrum of the copper complex (**3**) showed a peak at *m/z* 781.538 [M + Cu]^+^. The mass spectra are in the Supplementary Material (Figures S23–S26).

### 2.5. Optical Properties

**Figure 2:**
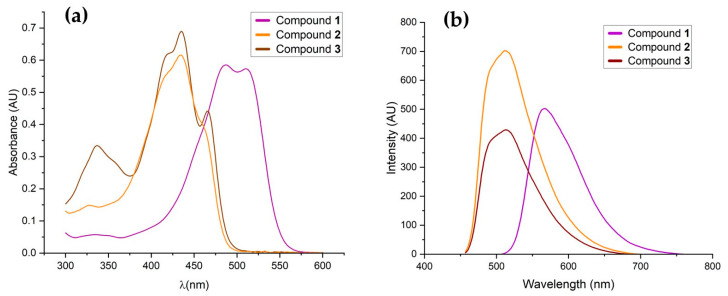
(**a**) Absorbance and (**b**) emission spectra of curcuminoid analogue-BF_2_ (**1**) (purple), analogue curcuminoid (**2**) (orange), and copper (II) complex (**3**) (brown) in DMSO.

The absorption and fluorescence spectra of compounds **1**–**3** were obtained using DMSO as solvent. The maximum absorption and emission are shown in Table 2 and the spectra are shown in Figure 2. Compound **1** showed two main maximum absorption bands in UV-Vis at 487 and 511 nm, while compounds **2** and **3** showed these at 434 and 435 nm, respectively, due to π-π* transitions.

The absorption curve of the copper complex (**3**) shows two shoulders at 422 and 466 nm attributed to charge transfer from the ligand to Cu(II), which has been reported in curcumin copper complexes [31,45]. The UV–Vis spectra of compounds **2** and **3** are provided in detail in the Supplementary Material (Figures S33 and S34).

The fluorescence spectra of compounds **1**–**3** are shown in Figure 2b. The emission maxima are similar for compounds **2** and **3** (Table 2). However, **1** exhibits fluorescence at a longer wavelength than 2. This tendency has been observed in curcuminoids-BF_2_ [46].

### 2.6. Infrared-FT Spectroscopy

The FT-IR spectrum of compound **1** shows medium-intensity bands at 1598 and 1587 cm^−1^ corresponding to stretching vibrations of C=O and C=C; a very intense band at 1504 cm^−1^ revealing C=C bond vibrations; and medium-intensity bands at 1384 and 1050 cm^−1^ evidencing B-O vibrations. The IR spectrum of compound **2** shows intense bands at 1604 and 1488 cm^−1^ corresponding to C=O and C=C, respectively. The FT-IR spectrum of copper (II) complex (**3**) exhibits medium-intensity bands at 1612 and 1598 cm^−1^, which correspond to C=O and C=C stretching, and at 1505 cm^−1^, an intense band is observed due to coordination with Cu (II); furthermore, new low-intensity bands are observed at 472 and 544 cm^−1^, indicating Cu-O bond vibrations. The FT-IR spectra of compounds **1**-**3** are in Supplementary Material (Figures S27–S29).

### 2.7. Single-Crystal X-Ray Diffraction

**Figure 3:**
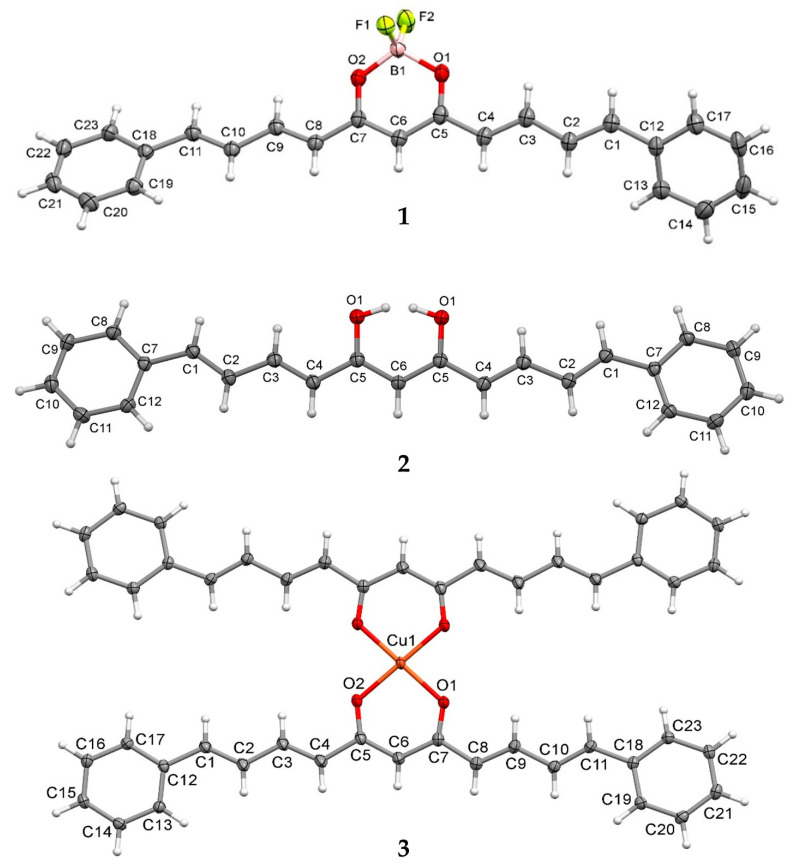
ORTEP representations of compounds **1**, **2**, and **3**. Displacement ellipsoids were drawn at the 50% probability level.

The crystallographic structures of compounds **1**–**3** were obtained, and ORTEP diagrams are shown in Figure 3; the crystallographic data are summarized in Table 3 and full details are available in the Supplementary Material (Tables S6–S21). The crystal structure of analogue-BF_2_ (**1**) shows a triclinic system. Three planes are defined, with a significant deviation in planarity in the unsaturated eleven-carbon chain (3E,6Z,8E)-7-hydroxyundeca-1,3,6,8,10-pentaen-5-one (RMS = 0.135 Å), whereas the phenyl moieties remain essentially planar (RMS = 0.002 and 0.004 Å). The angles formed between the central carbon chain plane and the phenyl groups are 25.18 and 30.78°. However, the phenyl group planes have a dihedral angle of 55.90° between them. The disposition of the -BF_2_ group is ca 90° with respect to the plane of the curcuminoid structure core.

The analogue (**2**) crystal structure has an orthorhombic system and crystallises as a THF solvate, which interacts with three analogue molecules in the crystal packing. The asymmetric unit consists of half of a curcuminoid analogue, completed by the crystallographic mirror plane, and the linear structure exhibits a significant deviation in planarity. The following two planes are defined: a (3E,6Z,8E)-7-hydroxyundeca-1,3,6,8,10-pentaen-5-one (RMS = 0.1710 Å) and the phenyl moiety (RMS = 0.003 Å), with both at 21.45° from each other. An intramolecular hydrogen bond within the keto-enol group is observed (distance O···H 1.67 Å).

The crystal of the copper complex (**3**) shows a monoclinic system and reveals a four-coordinate number for copper, adopting a square planar geometry. The asymmetric unit includes a ligand unit binding to a copper atom, positioned on an inversion centre. The planes formed by deprotonated (3E,6Z,8E)-7-hydroxyundeca-1,3,6,8,10-pentaen-5-one (RMS = 0.082 Å) and phenyl groups (0.0020 and 0.007 Å) show a slight deviation from planarity, forming an angle of 4.33° and 5.23°. The Cu–O bond distances are approximately 1.91 Å.

Intermolecular interactions in the crystal packing of compounds **1**–**3** were analysed in CrystalExplorer software through the Hirshfeld surface (HS) and the related 2D fingerprint plots.

**Figure 4:**
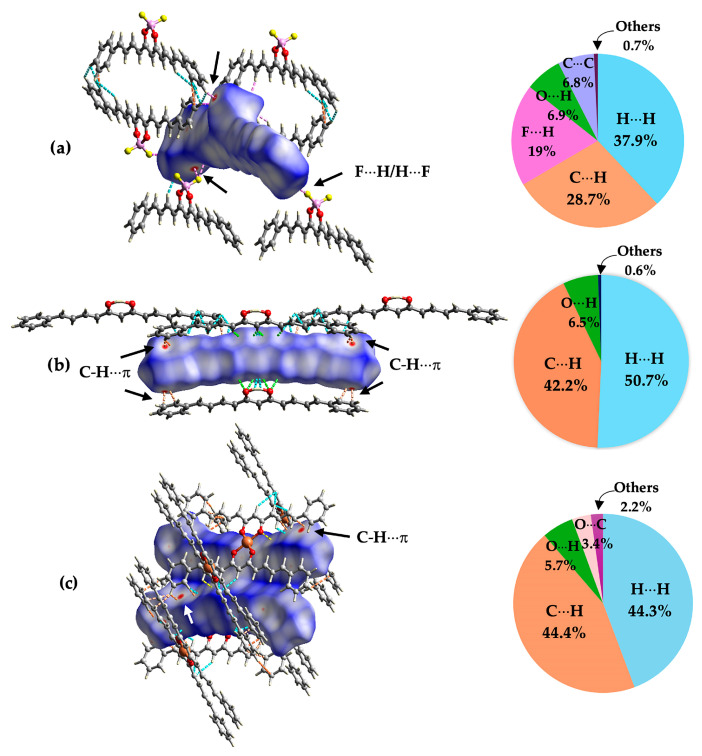
Hirshfeld surface and percentages of main intermolecular interaction contributions in the crystal of compounds (**a**) **1**, (**b**) **2**, and (**c**) **3**. The images show interactions at 2.9 Å (carbons are in grey, hydrogens in white, oxygens in red, fluorine in yellow, copper in orange and boron in pink).

The HSs were mapped using the descriptor *d*_norm_, which indicates that the intermolecular contact distances were normalised with the van der Waals radius of the atoms, involving the distance from the surface to the closest atom inside (d_i_) and outside (d_e_) [47,48]. In the HSs of compounds **1**–**3**, some areas were distinguished, with red corresponding to closer contacts (strong interactions), blue to those longer than the van der Waals radius, and white to distances equal to the sum of van der Waals radii [49]. In all crystal structures, longer-distance contacts (weak interactions) were predominant. In the HS of compound **1**, B-F···H-C interactions were represented by red areas (Figure 4), while in compounds **2** and **3**, red areas were established by C-H···π interactions with the phenyl rings.

The fingerprint plots (Figure S38) reveal that the contact contributions in the crystal packing of compounds **1**–**3** are mainly H···H (38–51%) and C···H/H···C (29–44%) interactions, which extend along the fingerprint plots and are related to the Van der Waals forces. However, in the case of analogue-BF_2,_ H···F/F···H interactions are relevant (19%) and appear in the two sharp spikes extending from the bottom to the top of the 2D fingerprint plot, suggesting strong interactions when the distance is shorter than the sum of Van der Waals radii [48]. Other interactions, like C-C (π-π contacts), are scarce.

### 2.8. Improvement of Water Solubility Through Association with β-Cyclodextrin (BCD) Formation and Biological Activity Evaluation

**Figure 5:**
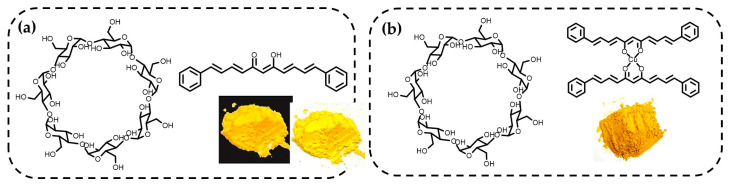
Formulations of (**a**) curcuminoid analogue with β-cyclodextrin **4** and (**b**) analogue copper complex with β-cyclodextrin **5**.

The analogue curcuminoid **2** and its copper complex **3** showed poor solubility in polar solvents; therefore, formulations with dry BCD were prepared using mechanochemistry. The solid obtained directly from the mill was shaken with water and filtered to obtain the association complex as a yellow and brown solid for **4** and **5**, respectively (Figure 5), which was analysed by NMR, infrared, and UV-Vis spectroscopy.

#### 2.8.1. Nuclear Magnetic Resonance of the Association Complexes with BCD

**Figure 6:**
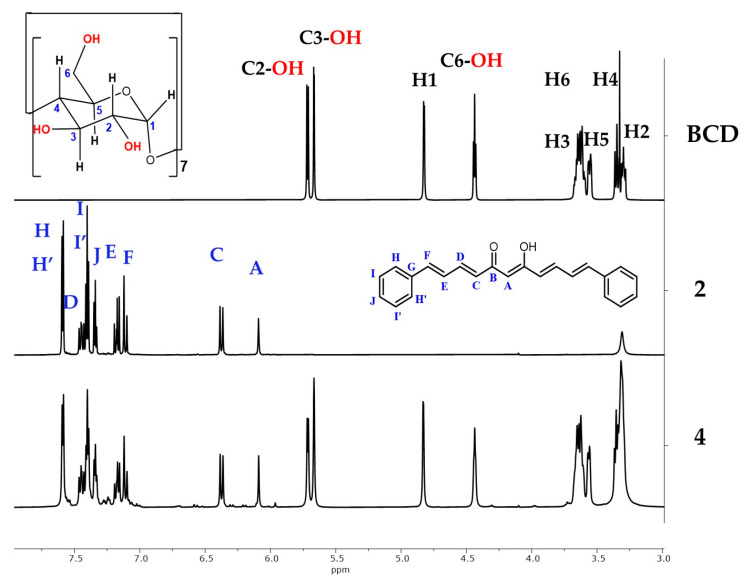
Stacked ^1^H-NMR spectra of BCD, compound (**2**), and association complex **4** (DMSO-d_6_, 700 MHz).

The shifts in the ^1^H-NMR spectrum of BCD were consistent with those reported in the literature [50]. The stacked ^1^H-NMR spectra of the free BCD and compounds **2** and **4** association complex are shown in Figure 6. The signals of BCD in the ^1^H-NMR spectrum of **4** are observed between 3 and 5.8 ppm. The ^1^H and ^13^C chemical shift changes defined by Δδ = δ_complex_ − δ_free_ (Tables S2 and S3) suggest interactions between BCD and **2** in the complex. The hydrogen shifts of BCD indicate a shielding effect on H3, H4, and H6 due to the anisotropy caused by π the electron of the conjugate system in **2**. A deshielding effect is observed on H1, H2, and C3-OH, with the highest Δδ values for H2, followed by H1, H4, and H6. Additionally, NOEs are observed between the enol-hydroxyl group of **2** and H2 and the C6-OH of BCD, indicating interactions with the exterior of BCD.

#### 2.8.2. UV-Vis and Infrared Spectroscopy of Association Complexes with BCD

**Figure 7:**
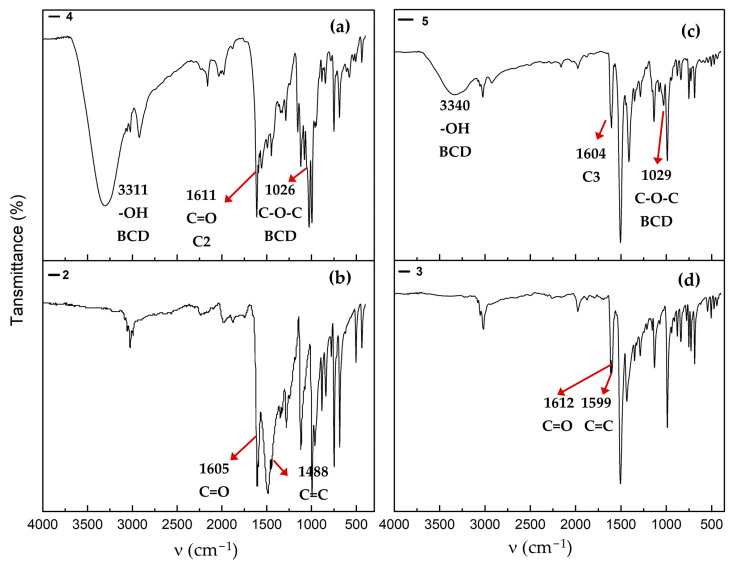
Prominent absorption bands in the infrared spectra of (**a**) association complex **4**, (**b**) compound **2**, (**c**) association complex **5**, and (**d**) copper complex **3**.

The FT-IR spectrum of the **4** complex shows two intense bands at 3311 and 1026 cm^−1^, corresponding to the -OH and C-O-C vibrations, respectively, which are related to the BCD structure (Figure 7a). Additionally, a broad and intense band absorption at 1488 cm^−1,^ attributed to the vibration of the C=C bond of compound **2** (Figure 7b), is not observed in the complex with BCD, which may be attributed to changes in band intensity and wavelength number.

The FT-IR spectrum of the inclusion complex of **5** displays the characteristic BCD bands at 3340 cm^−1^ (O–H stretching) and 1029 cm^−1^ (C–O–C stretching), which shift from 3307 and 1021 cm^−1^, respectively (see FT-IR spectrum of BCD, Figure S32). Minor shifts in wavelength number are also observed in the bands assigned to the C=O and C=C stretching vibrations, which appear at 1612 and 1599 cm^−1;^ however, in the spectrum of the BCD complex, they collapse at 1604 cm^−1^ (Figure 7c,d). FT-IR spectra of **4** and **5** are provided in detail in the Supplementary Materials (Figures S30 and S31).

The UV-Vis absorption maxima of the association complexes **4** and **5** in DMSO were observed at 431 and 438 nm, respectively. In comparison, the free forms of compounds **2** and **3** exhibited absorption maxima at 434 and 435 nm, respectively, as shown in Table 2. These results reveal a hypsochromic shift for compound **2** upon complexation with BCD, whereas compound **3** exhibited a bathochromic shift upon BCD association. 

The concentration of association complexes **4** and **5** was determined by UV-Vis spectroscopy, yielding values of 393.60 µg/mg and 685.94 µg/mg, respectively.

Additionally, the thermal profiles obtained by Thermogravimetric Analysis (TGA) and Differential Scanning Calorimetry (DSC) indicated the formation of a new phase in compounds **4** and **5**, as evidenced by the disappearance of the melting points observed in the free compounds (**2** and **3**) and by temperature changes of exothermic peaks. In the Supplementary Material (Graphics S1 and S2), a detailed analysis of the thermograms is provided.

### 2.9. Biological Activity

The antiproliferative and α-glucosidase inhibitory activities of compounds **2** and **3**, as well as their BCD complexes **4** and **5**, were evaluated.

Antiproliferative activity was assessed in various cancer cell lines using the sulforhodamine B assay at a concentration of 10 µM. The resulting cell growth inhibition percentages are presented in Table S4. Neither the compounds nor their BCD complexes demonstrated antiproliferative activity at this concentration.

**Figure 8:**
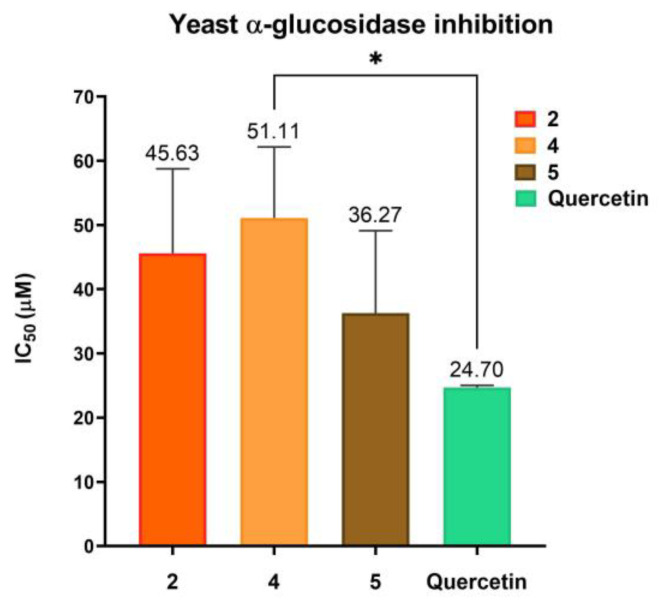
Yeast α-glucosidase inhibition activity of compound **2** and BCD association complexes **4** and **5**, expressed as half inhibitory concentration. *n* = 3, 4, 5; * *p* < 0.05.

A yeast α-glucosidase inhibition assay was performed for compounds **2**, **3**, and their BCD complexes **4** and **5** using quercetin as a positive control; complete data are shown in Table S5, and in Figure 8, the half-inhibition concentrations (IC_50_) are shown.

The inhibitory activity of compound **2** shows no change concerning its BCD complex **4**, exhibiting IC_50_ values of 45.63 ± 13.12 and 51.11 ± 11.05 µM, respectively. In contrast, compound **3** does not have important inhibitory activity against α- glucosidase (16.19 ± 3.18% at 100 µM, Table S5); however, in complex with BCD, it shows enhanced activity, exhibiting IC_50_ = 36.27 ± 12.85 µM, which is comparable with compound **2**’s inhibitory activity. The enzyme inhibitory activities of **2** and **5** do not show a significant difference from quercetin activity.

## 3. Discussion

Curcumin and curcuminoids are fascinating compounds due to their biological activity and applications as dyes and photosensitizers [10,35,51,52]. Research on the synthesis of analogue curcuminoids has centred on modifications around the aromatic or β-diketone group [53]; however, analogues with an extended central chain are unexplored, and a few reports describe spectroscopic and biological properties [14,32,33,34,35]. The synthesis of a curcuminoid analogue with an eleven-carbon central chain followed the previously reported methodology through a condensation reaction of cinnamaldehyde and synthon, followed by the removal of the difluoroboron group. In this final step, microwave irradiation and reflux resulted in efficient methodologies for obtaining compound **2** in good yield (81–85%). The microwave irradiation method required less time compared to refluxing conditions. This synthetic route to synthesise compound 2 led to an improvement in time and yield compared to the reported method by Kazantzis et al., which used an acetone–boric oxide complex as the first step (63%) [35].

Overlap suppression of signals in 1D and 2D NMR experiments at 700 MHz led to a complete hydrogen and carbon assignment, observing the characteristic deshielding effect of the -BF_2_ group over vinyl and aromatic hydrogens in compound **1**. In addition, the influence of the solvents DMSO-d_6_ and CDCl_3_ on the multiplicity of H_D_, H_E_, and H_F_ was evidenced in the ^1^H-NMR spectrum of compound **2**. In addition, the NOESY spectrum showed distinctive hydrogen interactions between H_D_-H_A_ and H_H_-Hc when CDCl_3_ was used as solvent. Such an effect can be attributed to the polarity and viscosity of the deuterated solvent, which favours different conformer populations [54]. This fact suggests that the molecular flexibility of compound **2** is due to the extended central chain, in comparison with the parent diphenylcurcuminoid, for which no changes in the multiplicity of proton signals in CDCl_3_ and DMSO-d_6_ have been reported in the literature [24,55].

Similarly, the EPR spectrum of compound **3** exhibits the coexistence of two species and the g‖, gꞱ, and A‖ and F values agree with the previously reported homoleptic complexes of copper with curcuminoids and curcumin, whose predominant species exhibit a square-planar geometry with distortion [24,31]. In contrast, none of the reported homoleptic copper–curcuminoid complexes have shown evidence for the coexistence of different species. Also, a change in solvent can alter the electronic distribution and geometry of the coordination sphere of copper complexes [56].

The observed optical properties show that complexation with the -BF_2_ group in compound **1** gives rise to red-shifted absorption bands compared to the curcuminoid analogue **2**; this effect has been observed in reported curcuminoid–boron complexes [41,57,58]. Additionally, Bai and coworkers studied curcuminoids-BF_2_, finding that an extra double bond in the central chain led to a red shift compared to seven-carbon-chain curcuminoids-BF_2_ [14]. On the other hand, Tahay and coworkers reported a red shift in the maximum absorption of compound **2** compared to diphenylcurucuminoid, attributed to the length of the π-conjugation [34]. The extinction coefficient of **2** exceeds that of curcumin and bisdemethoxycurcumin, agreeing with the reported value [35]. Copper complex **3** demonstrates an even higher extinction coefficient (Table 2), indicating a better interaction with light, a desirable property for applications in dyes and photosensitizers [14]. Additionally, this opens up the possibility of phototherapeutic applicability.

In the present work, the crystal structures of compounds **1**–**3** are reported for the first time, although Bai and coworkers previously reported a polymorphic monoclinic crystal of compound **1** [14]. Cooper complex **3** showed a square planar geometry, which is predominantly observed in reported curcuminoid copper complexes [24,31]. In addition, the copper complex exhibited the best planarity, revealing efficient conjugation, as evidenced by a significant extinction coefficient, which could improve its optical properties, a phenomenon observed in copper complexes with extended conjugation [59].

Hirshfeld surfaces and the related 2D fingerprint plots of crystal structures reveal that the main interactions are governed by Van der Waals forces (H···H and C-H···π), with similar contributions reported in the crystal packing for a copper complex of curcumin [31], an asymmetric curcuminoid [60], and zinc complexes of curcuminoids [61]. Closer contacts (strong interactions) are displayed on the extreme sides of aromatic rings through C-H···π interactions in compounds **2** and **3**. In contrast, in compound **1**, the BF_2_ region, through B-F···H-C interactions, reveals strong interactions.

These predominant interactions described for compounds **2** and **3** are related to a reduced solubility in polar solvents, which led to the development of BCD-based formulations and further biological evaluation. The improvement in solubility enhances potential applications, particularly considering that a cytotoxic effect of compound **2** against a prostate cancer cell line has been documented, leading to increased efficacy under irradiation in photodynamic therapy experiments [28].

The spectroscopic analysis of BCD complexes of compounds **2** and **3** demonstrates guest–host interactions; ^1^H-NMR shifts reveal shielding and deshielding effects attributed to guest aromatic moieties in guest molecules, an effect that has been reported in complexes with BCD [62,63,64]. These results suggest a partial insertion of compound **2** in the cavity of BCD through the interaction of the π-conjugated system with H3, which resides in the cavity near the wider rim of BCD [65]. In addition, NOE’s correlations confirm interactions with the exterior of BCD. Infrared spectra show changes in intensity and shifts, mainly in bands related to the C=C and C=O stretching vibrations of the guest molecules and the O-H or C-O-C stretching vibrations of BCD. This effect is observed in the association of curcumin or azomethine with BCD, where the disappearance of the band corresponding to the aromatic ring is reported, suggesting the introduction of benzene rings into the BCD cavity [66]. Likewise, changes in wavenumber are a consequence of interactions between guest and host [67].

The UV-Vis spectra of **4** and **5** association complexes show both bathochromic and hypsochromic effects in the maximum absorption, respectively. The literature reports bathochromic effects for inclusion complexes of ursolic acid, oleanolic acid, and sulfabenzamide with BCD, attributed to changes in the medium’s polarity and to interactions within the BCD cavity or at its outer surface [68,69]. The results provide evidence for the effective formation of complexes with BCD.

We evaluated compounds **2**, **3**, and their BCD complexes for their antiproliferative and α-glucosidase inhibition effects. All compounds showed no antiproliferative activity against different cancer cell lines; however, compound two and its BCD complex inhibited α-glucosidase without a significant difference, with comparable curcumin inhibitory activity (IC_50_ = 37.2/78.2 µM) and activities better than demetoxicurcumin (IC_50_ = 82.4 µM) and bisdemetoxicurcumin (IC_50_ = 90.6 µM) [28,70]. In the case of **3**, there was no inhibition activity at high concentrations; however, its BCD complex **5** exhibited a notable improvement in inhibition activity (IC_50_ = 36.27 µM), highlighting the importance of copper (II) and the formulation with BCD. Reports indicate that the copper ion exhibits better α-glucosidase inhibition than divalent metal ions like Zn^2+^, Ca^2+^, Mg^2+^, and Mn^2+^ [71]. In the same manner, metallorganic copper complexes have also shown better enzyme inhibitory activity than free ligands [72,73]. Even the complex of hesperetin with copper exerts a synergistic effect with acarbose, and kinetic studies suggest that its copper complex could interact with the active site pocket, inhibiting carbohydrate hydrolysis [73]. The latter fact reinforces the importance of the formulation of **3** with BCD, aiming to improve its potency as inhibitor of α-glucosidase.

## 4. Materials and Methods

2,4-Pentanodione, boron trifluoride THF complex (CAS 462-34-0), cinnamaldehyde (CAS 080687), copper acetate (CAS 142-71-2), tributyl borate (CAS 688-74-4), *N*-butylamine (CAS 109-73-9), β-cyclodextrin (CAS 7585-39-9), triethylamine (CAS 142-71-2), and solvents of HPLC grade were commercially acquired from Sigma Aldrich^®^ (St. Louis, MO, USA). Cinnamaldehyde (40 mL) was previously distilled under reduced pressure (10 mmHg), collecting the cinnamaldehyde fraction at 80 °C.

Melting points were measured using an Electrothermal Engineering IA9100 digital apparatus (Electrothermal Engineering Ltd, Essex, UK), and values are uncorrected.

### 4.1. Analytical Determinations

Infrared spectra were recorded on an FT-IR NICOLET IS-50 (Thermo Fisher Scientific, Waltham, MA, USA) using the attenuated total reflectance (ATR) technique (4000–400 cm^−1^).

^1^H, ^13^C NMR, and two-dimensional spectra (COSY, HSQC, HMBC, NOESY, and ROESY) were recorded on a Bruker Avance III HD 700 MHz spectrometer (Bruker, Rheinstetten, Germany) using DMSO-d_6_ as a solvent and reference. Spectra were processed using MestReNova software 14.2.0 (Mestrelab Research S.L., Santiago de Compostela, Spain) [74].

The mass spectrum of compound **3** was acquired on a Bruker Microflex MALDI-TOF spectrometer (Bruker Daltonics, Bremen, Germany) equipped with a nitrogen laser (λ = 337 nm). Analyses were performed in positive ion mode with an acceleration voltage of 20 kV, using 2,5-dihydroxybenzoic acid (DHB) as the matrix.

The electron paramagnetic resonance (EPR) spectrum was recorded on a JEOL JES-TE300 (JEOL Ltd., Akishima, Tokyo, Japan) spectrometer operating in the X-band at 9.5 GHz. Magnetic field calibration was performed using an ES-FC5 NMR field meter (JEOL Ltd., Tokio, Japan). Spectral acquisition and data processing were carried out with the ES-IPRITS data system (v.3, Jeol Ltd., Tokyo, Japan, 1997). The sample was prepared as a 3 mM solution in THF and placed in a 5 mm quartz tube. Measurements were conducted at 77 K, with the magnetic field centred at 300.0 mT.

The mass spectra of compounds 1 and 2 were recorded on a Bruker Esquire 6000 spectrometer (Bruker Daltonics, Bremen, Germany) equipped with an electrospray ionisation (ESI) source, operated in both positive and negative ion modes. The drying temperature was set at 300 °C, and the nebuliser pressure was maintained at 10 psi.

HPLC-UV was performed on an Agilent 1200 (Agilent Technologies, Waldbronn, Germany) with a UV-Vis diode array detector Waters 2996. Separation was performed on Eclipse Plus C18 (2.1 × 100 mm × 3.5 µm) column using a gradient of methanol and water starting at 60:40 and ending with 100% of methanol at a flow rate of 0.2 mL/min. Samples were dissolved in DMSO.

Single-crystal X-ray diffraction was performed on a Bruker D8 Venture diffractometer (Bruker, Karlsruhe, Germany), CCD equipped with a graphite monochromator, and MoKα as the source of radiation (λ = 0.71073) at 150 K for compounds **1** and **2** and 100 K for compound **3**. Direct methods by SHELXS (v2019/3) solved the structures, [75], which were refined by full-matrix least-squares on F^2^ using SHELXL (v2014/2) [76]. All non-hydrogen atoms were refined anisotropically. For compound **2,** the symmetry transformation #1-x+1,y,z was used to generate equivalent atoms. The structure was visualised using the MERCURY software (v.2025.1.1, Cambridge Crystallographic Data Centre, Cambridge, UK) [77] and RMS values were calculated in Olex2 (v.1.5, OlexSys Ltd., Durham, UK) [78].

Intermolecular interactions present in the crystal packing were analysed using CrystalExplorer (v.21.5, University of Western Australia, Perth, Australia) [79]. Hirshfeld surfaces and finger 2D plots were generated from the crystallographic information files (CIFs) obtained by single-crystal X-ray diffraction.

UV-Vis spectra were determined on Shimadzu UV-160 and Shimadzu UV-1800 spectrophotometers (Shimadzu, Kyoto, Japan) in the range of 200–800 nm. Measurements were performed using quartz cuvettes of 1.0 cm, and DMSO was used as a blank.

Fluorescence spectra were recorded on an Agilent Cary Eclipse spectrofluorometer (Agilent Technologies, Santa Clara, CA, USA) using quartz cuvettes with a length of 1.0 cm. Samples were prepared at a concentration of 3 µM using DMSO as the solvent and measured at 25 °C. Each compound was excited at its maximum absorption wavelength (compound **1**: 487 nm; compound **2**: 434 nm; and compound **3**: 435 nm). Excitation and emission slit widths were set to 5 nm, and the scan rate was 600 nm/min.

Complexes with β-cyclodextrin were prepared in a planetary ball mill PQ-N04 (Across International, Livingston, NJ, USA) using zirconia jars of 100 mL and balls of 0.5 cm in diameter.

### 4.2. Synthetic Procedures

Synthon was prepared using 2,4-pentanodione and boron trifluoride–THF complex, as previously reported [40].

**Compound 1**: Distilled cinnamaldehyde (7.000 g, 53 mmol) was placed in a 125 mL Erlenmeyer flask and dissolved in ethyl acetate (20 mL). Tributyl borate (6.70 mL, 28 mmol) was added, and the mixture was stirred to ensure complete homogenization. The resulting solution was added to a 250 mL round-bottom flask containing the synthon (4.70 g, 53 mmol) previously dissolved in ethyl acetate (47 mL). Separately, n-butylamine (2.80 mL, 28 mmol) was dissolved in ethyl acetate (6 mL) and placed in an addition funnel. This amine solution was added dropwise to the reaction mixture under continuous stirring. The reaction was maintained at room temperature with stirring for 4 h to afford a purple precipitate, which was filtered and washed with a 90:10 water/acetone mixture. Crystallisation was performed in an acetone–tetrahydrofuran mixture. Yield: 68%, m.p. = 220 °C, ^1^H NMR (700 MHz, Chloroform-*d*) δ 7.82 (t, *J* = 14.8 Hz, 2H), 7.53–7.49 (m, 4H), 7.41–7.31 (m, 6H), 7.09 (d, *J* = 15.4 Hz, 2H), 6.98 (t, *J* = 15.3 Hz, 2H), 6.27 (d, *J* = 14.8 Hz, 2H), 5.93 (s, 1H). ^13^C NMR (176 MHz, CDCl_3_) δ 179.59, 147.66, 145.13, 135.80, 130.27, 129.14, 128.01, 126.64, 124.22, 102.22. FT-IR (ATR) 3024, 2158, 11972, 1172, 1667, 1599 cm^−1^, 1586.55 cm^−1^, 1503, 1383, 1130, 1050, 984 885, 849, 752, 689. MS (ESI-) *m/z* 411.6 [M + Cl]^−^.

**Compound 2**: Reflux: In a 1 L round-bottom flask, compound 1 (3.000 g, 8 mmol) was dissolved in DMSO (7.5 mL), then methanol (400 mL) and water (2.5 mL) were added. Triethylamine (2.20 mL, 16 mmol) was added to the solution, and the reaction mixture was stirred under reflux for 10 h. The orange precipitate was filtered and washed with a solution of acetone–water (90:10, *v*/*v*). Yield: 81%, orange powder, mp: 189–191 °C. Crystallisation was performed in tetrahydrofuran.

Microwave irradiation: The experimental procedure was carried out according to the methodological conditions described in the literature [80,81], and some variations were made. Compound 1 (0.400 g, 1 mol) and sodium oxalate (0.250 g, 1.8 mol) were placed in a sealed microwave reaction vessel, and 20 mL of methanol–water (75:25, *v*/*v*) solution was added. The reaction mixture was subjected to microwave irradiation at 140 °C for 10 min. The precipitate was collected by vacuum filtration and washed with water. Recrystallisation from ethyl acetate-hexane afforded the orange solid product. Yield: 85%, orange powder, mp: 189–191 °C.

^1^H NMR (700 MHz, DMSO-d_6_) δ 16.06 (br s, 1H), 7.59 (d, *J* = 7.5 Hz, 4H), 7.45 (t, *J* = 14.9 Hz, 2H), 7.40 (t, *J* = 7.6 Hz, 4H), 7.34 (t, *J* = 7.3 Hz, 2H), 7.18 (t, *J* = 15.4 Hz, 2H), 7.11 (d, *J* = 15.6 Hz, 2H), 6.38 (d, *J* = 15.1 Hz, 2H), 6.09 (s, 1H). ^13^C NMR (176 MHz, DMSO-d_6_) δ 182.88, 141.00, 140.34, 136.10, 129.06, 128.87, 127.74, 127.36, 127.25, 101.3. FT-IR (ATR) 3023 cm^−1^, 2992 cm^−1^, 2239 cm^−1^, 1985 cm^−1^, 1605 cm^−1^, 1488 cm^−1^, 1281 cm^−1^, 1119 cm^−1^, 993 cm^−1^, 747 cm^−1^, 684 cm^−1^, 502 cm^−1^, 436 cm^−1^. MS (ESI+) *m*/*z* = 329.9 [M + H]^+^. Purity 99.85% (HPLC).

**Compound 3**: Compound **2** (0.400 g, 1.20 mmol) was dissolved in 70 mL of ethyl acetate in a round-bottom flask. Separately, copper (II) acetate (0.1320 g, 0.7 mmol) was dissolved in a methanol–water solution. The resulting solution was added dropwise to the reaction mixture, which was stirred at room temperature for 36 h. The precipitate was filtered and washed with a water–acetone solution (90:10, *v*/*v*). Crystallisation was carried out in acetonitrile and acetonitrile–tetrahydrofuran mixture. Brown powder, yield: 90%, decomposition temperature: 269–270 °C. FT-IR (ATR) 3052 cm^−1^, 3017 cm^−1^, 1975 cm^−1^, 1873 cm^−1^, 1794 cm^−1^, 1691.05 cm^−1^, 1612 cm^−1^, 1599 cm^−1^, 1505 cm^−1^, 1435 cm^−1^, 1130 cm^−1^, 988 cm^−1^, 686 cm^−1^, 544 cm^−1^, 504 cm^−1^, 472 cm^−1^ and 441 cm^−1^. MS (MALDI-TOF) *m*/*z* = 781.538 [M + Cu]^+^. Purity 100% (HPLC).

### 4.3. Preparation of Association Complexes with β-Cyclodextrin

The formulations with β-cyclodextrin were prepared using a planetary mill ball.

Compounds **2** (0.120 g, 0.365 mmol) or **3** (0.120 g, 0.160 mmol) and β-cyclodextrin (2 equivalents), previously dried in a conventional microwave, were placed in separate zirconia jars with 15 balls (0.5 cm in diameter). The mixtures were subjected to four 30 min mill cycles at 500 rpm, alternating between clockwise and counterclockwise rotation. The unreacted β-cyclodextrin was removed by extraction with water (10 mL) under stirring for 10 min, and the solids were collected by vacuum filtration.

**Compound 4**: yellow powder, yield 45%, m.p. 281–285 °C. ^1^H NMR (700 MHz, DMSO-d_6_) δ 16.06 (br s, 1H), 7.59 (d, *J* = 7.6 Hz, 4H), 7.45 (t, *J* = 14.9 Hz, 2H), 7.40 (t, *J* = 7.6 Hz, 4H), 7.34 (t, *J* = 7.4 Hz, 2H), 7.17 (t, *J* = 15.6 Hz, 2H), 7.11 (d, *J* = 15.5 Hz, 2H), 6.37 (d, *J* = 15.1 Hz, 2H), 6.09 (s, 1H), 5.71 (d, *J* = 6.9 Hz, 4H), 5.67 (d, *J* = 2.55 Hz, 3H), 4.83 (d, *J* = 3.64 Hz, 3H), 4.44 (t, *J* = 5.60, 3H), 3.63 (m, 10H), 3.56 (m, 3H), 3.35 (m, 4H), 3.30 (m, 4H). ^13^C NMR (176 MHz, DMSO-d_6_) δ 182.89, 141.02, 140.35, 136.10, 129.07, 128.88, 127.74, 127.36, 127.26, 101.93, 101.36, 81.53, 73.03, 72.41, 72.03, 59.90. FT-IR (ATR) 3312 cm^−1^, 2922 cm^−1^, 2160 cm^−1^, 2033 cm^−1^, 1979 cm^−1^, 1611 cm^−1^, 1557 cm^−1^, 1447 cm^−1^, 1026 cm^−1^, 996 cm^−1^, 749 cm^−1^, 688 cm^−1^, 577 cm^−1^, 505 cm^−1^, 438 cm^−1^. Concentration by UV/VIS = 393.60 µg/mg.

**Compound 5**: brown powder, yield 35%, decomposition temperature = 300 °C, FT-IR (ATR) 3340 cm^−1^, 3022 cm^−1^, 2159 cm^−1^, 1976 cm^−1^, 1604 cm^−1^, 1504 cm^−1^, 1411 cm^−1^, 1135 cm^−1^, 1029 cm^−1^, 988 cm^−1^, 750 cm^−1^, 687 cm^−1^, 504 cm^−1^, 471 cm^−1^, 436 cm^−1^. Concentration by UV/VIS = 685.94 µg/mg.

### 4.4. Antiproliferative Activity

Antiproliferative activity was evaluated by Sulforhodamine B assay against colon cancer (HCT-15), breast cancer (MCF-7), leukaemia (K-262 CLM), central nervous system glial (U-251), and prostate cancer (PC-3) cell lines, which the National Cancer Institute supplied. The cell lines were cultured in RPMI-1640 medium supplemented with 10% fetal bovine serum, 2 mM L-glutamine, 10,000 units per mL penicillin G, 100 µg/mL streptomycin sulfate, and 0.25 µg/mL amphotericin B Gibco (Thermo Fisher Scientific, Grand Island, NY, USA). They were incubated at 37 °C in an atmosphere of 5% CO_2_ and 95% humidity. For the assay, cells were suspended in a 0.1% trypsin-EDTA solution (Gibco). Cell viability was counted using a hematocytometer and diluted with medium to achieve the following densities: 5 × 10^4^ cells/mL (SKLU-1, K562) and 7.5 × 10^4^ cells/mL (U251, PC-3). Then, 100 µL/well of the aforementioned cell suspensions were seeded in 96-well plates and incubated at 37 °C to allow for cell adhesion. A control plate (time zero evaluation) was used, containing the same volumes of each cell line and the target (RPMI-1640 growth medium).

After 24 h, the cells were treated with 100 µL of the compounds to be evaluated, previously dissolved in RPMI-1640 medium and 1% DMSO, and then incubated for 48 h at 37 °C in a 5% CO_2_ atmosphere. Only 100 µL of RPMI-1640 culture medium was added to the control plate.

After 48 h, the adherent cells were fixed by adding 50 µL of a cold 50% (m/vol) trifluoroacetic acid solution and incubated at 4 °C for 60 min. The supernatant was then removed, washed three times with water, and allowed to dry at room temperature. The trifluoroacetic acid-fixed cells were then treated with 100 µL of an SRB solution (0.4% m/vol in 1% acetic acid) for 30 min at room temperature. Unbound SRB was removed with four washes with 1% acetic acid, and bound SRB was extracted with 100 µL of unbuffered 10 mM Tris base. The optical density of the treated samples was determined in a Bio Kinetics microplate reader at a wavelength of 515 nm (Synergy HT, BioTek, Winooski, VT, USA). The colour intensity was directly proportional to the number of live cells.

### 4.5. Inhibition of Yeast α-Glucosidase

The inhibition of α-glucosidase was evaluated using a previously adapted method [82,83]. Briefly, a solution (25 µL) of tested samples in DMSO-H_2_O 1:1 was added to 150 µL of phosphate buffer (PBS, 67 mM, pH 6.8) and incubated at 37 °C for 10 min with 25 µL of reduced glutatione (3 mM in PBS) and 25 µL of of α-glucosidase type I solution (0.2 U/mL in PBS, CAS G5003-100UN) (Sigma Aldrich, Toluca, Estado de México, Mexico). Subsequently, 25 µL of the substrate solution (23.2 mM *p*-nitrophenyl-α-D-glucopyranoside, CAS N1377-1G, in PBS) (Sigma Aldrich, Toluca, Estado de México, Mexico) was added, and the mixture was incubated at 37 °C for 15 min under shaking. The reaction mixture was stopped by the addition of a CaCO_3_ 1M solution (50 µL), followed by agitation for 5 min. The optical density was measured at 405 nm, and quercetin was used as a positive standard. The percentage of inhibition was calculated according to the following equation:

Inhibition (%) = [(Acontrol − Asample)/Acontrol] × 100

where A is the absorbance at 405 nm of the sample and the control.

## 5. Conclusions

The successful synthesis of a group of extended-chain curcuminoid analogues containing eleven carbon atoms as the primary component is achieved with commendable yields. The alternative use of microwave radiation or reflux demonstrates the effectiveness of these methods in the final stages of the synthesis process. Moreover, we obtain the crystal structures of ligand **2** and its BF_2_ complex, as well as those of the first copper complexes, **1** and **3**. We also provide the ^1^H and ^13^C NMR assignments of compounds **1** and **2** in detail. Notably, the conformational changes of compound **2** are dependent on the solvent, highlighting a degree of flexibility within the unsaturated central chain. All compounds (**1**–**3**) exhibit promising optical properties, showing a red shift in maximum absorption and a high extinction coefficient compared to their seven-carbon curcuminoid counterparts.

Additionally, we report a formulation of compounds **2** and **3** with BCD, underscoring the effectiveness of the mechanochemical method. Compounds **2** and **3** exhibit moderate/good inhibitory activity against α-glucosidase and are comparable to the positive control. Notably, the formulation of a copper complex with BCD **5** is crucial in enhancing enzyme inhibition activity, highlighting the significance of solubility improvement in these compounds for biological activity evaluation.

## Figures and Tables

**Table 1:** ^1^H and ^13^C chemical shifts of compounds **1** and **2** (700 MHz).

	Compound 1 (CDCl_3_)		Compound 2 (DMSO-d_6_)		Compound 2 (CDCl_3_)	
	^13^C (δ, ppm)	^1^H (δ, ppm)	^13^C (δ, ppm)	^1^H (δ, ppm)	^13^C (δ, ppm)	^1^H (δ, ppm)
A	102.22	5.93	101.34	6.09 (s)	101.87	5.71 (s)
B	179.59		182.88		183.24	
C	124.22	6.27	127.74	6.38 (d)	127.87	6.18 (d)
D	147.66	7.82	141.00	7.45 (dd)	140.88	7.44 (m)
E	126.64	6.98	127.36	7.18 (dd)	127.22	6.92 (d) *
F	145.13	7.09	140.34	7.11 (d)	140.38	6.93 (d) *
G	135.8		136.10		136.44	
H, H’	128.01	7.52	127.25	7.59 (m) ***	127.22	7.48 (m)
I, I’	129.14	7.38	128.87	7.40 (m) **	128.97	7.36 (m)
J	130.27	7.38	129.06	7.34 (m) **	129.15	7.31 (m)
-OH				16.06 (br, s)		15.85 (br, s)

* Superimposed signals, shifts can exchange; ** triplet-like; *** doublet-like.

**Table 2:** Maximum absorption and emission bands and extinction coefficient (ε) of curcuminoid analogue-BF_2_ (**1**), curcuminoid analogue (**2**), and cooper complex (**3**) in DMSO.

Compound	Absorption λ (nm)	ε (M^−1^·cm^−1^) [log ε]	Emission λ (nm)
**1**	487	75,372.75 [4.9]	567.01
**2**	434	63,305.95 [4.8]	511.04
**3**	435	120,156.79 [5.1]	512.98

**Table 3:** Crystal data of compounds **1**, **2**, and **3**.

Compound	1	2	3
CCDC (deposition number)	2,483,477	2,483,476	2,483,475
Empirical formula	C_23_H_19_BF_2_O_2_	C_27_H_28_O_3_	C_46_H_38_CuO_4_
Formula weight	376.19	400.49	718.30
Crystal system	Triclinic	Orthorhombic	Monoclinic
Space group	P-1	Cmc2_1_	C2/c
Unit cell dimensions			
a (Å)	a = 9.1069 (6)	a = 28.5258 (9)	a = 41.1114 (11)
b (Å)	b = 10.0955 (7)	b = 9.7389 (3)	b = 5.56190 (10)
c (Å)	c = 10.7715 (7)	c = 7.6957 (2)	c = 16.1765 (4)
α (°)	α = 74.270 (2)	α = 90	α = 90
β (°)	β = 85.586 (2)	β = 90	β = 100.2810 (10)
γ (°)	γ = 85.457 (2)	γ = 90	γ = 90
Volume (Å^3^)	948.65 (11)	2137.94 (11)	3639.49 (15)
Z	2	4	4
Density (calculate) (mg/m^3^)	1.317	1.244	1.311
Absorption coefficient (mm^−1^)	0.095	0.080	0.644
F (000)	392	856	1500
Crystal size (mm^3^)	0.291 × 0.239 × 0.178	0.400 × 0.280 × 0.200	0.346 × 0.106 × 0.089

## Data Availability

The original contributions presented in the study are included in the article/Supplementary Materials, further inquiries can be directed to the corresponding authors.

## Supplementary Materials

The following supporting information can be downloaded at: https://www.mdpi.com/article/10.3390/molecules30193943/s1, Figure S1: ^1^H-NMR spectrum of compound **1** (DMSO-d6, 700 MHz); Figure S2: ^13^C-NMR spectrum of compound **1** (DMSO-d_6_, 175 MHz); Figure S3: COSY spectrum of compound **1** (DMSO-d_6_); Figure S4: HSQC spectrum of compound **1** (DMSO-d_6_); Figure S5: HMBC spectrum of compound **1** (DMSO-d_6_); Figure S6: ^1^H-NMR spectrum of compound **2** (DMSO-d6, 700 MHz); Figure S7: ^13^C-NMR spectrum of compound **2** (DMSO-d_6_, 175 MHz); Figure S8: COSY spectrum of compound **2** (DMSO-d_6_); Figure S9: HSQC spectrum of compound **2** (DMSO-d_6_); Figure S10: HMBC spectrum of compound **2** (DMSO-d_6_); Figure S11: NOESY spectrum of compound **2** (DMSO-d_6_, 700 MHz); Figure S12: ^1^H-NMR spectrum of compound **2** (CDCl_3_, 700 MHz); Figure S13: ^13^C-NMR spectrum of compound **2** (CDCl_3_, 175 MHz); Figure S14: COSY spectrum of compound **2** (CDCl_3_); Figure S15: HSQC spectrum of compound **2** (CDCl_3_); Figure S16: HMBC spectrum of compound **2** (CDCl_3_); Figure S17: NOESY spectrum of compound **2** (CDCl_3_, 700 MHz); Figure S18: ^1^H-NMR spectrum of association complex 4(DMSO-d_6_, 700 MHz); Figure S19: ^13^C-NMR spectrum of spectrum of association complex 4 (DMSO-d_6_, 175 MHz); Figure S20: NOESY spectrum of spectrum of association complex 4 (DMSO-d_6_, 700 MHz); Figure S21: NOESY spectrum expansion of spectrum of association complex 4 (DMSO-d_6_, 700 MHz); Figure S22: Electron paramagnetic resonance spectrum of compound **3** (THF); Figure S23: Mass spectrometry spectrum of compound **1** (ESI-); Figure S24: Mass spectrometry spectrum of compound **2** (ESI+); Figure S25: Mass spectrometry spectrum of compound **3** (MALDI-TOF); Figure S26: Mass spectrometry spectrum of compound **3**, expansion (MALDI-TOF); Figure S27: Infrared spectrum of compound **1** (ATR); Figure S28: Infrared spectrum of compound **2** (ATR); Figure S29: Infrared spectrum of compound **3** (ATR); Figure S30: Infrared spectrum of spectrum of association complex 4 (ATR); Figure S31: Infrared spectrum of spectrum of association complex 5 (ATR); Figure S32: Infrared spectrum of β-cyclodextrin (ATR); Figure S33: UV-Vis spectrum of compound **2** (DMSO); Figure S34: UV-Vis spectrum of compound **3** (DMSO); Figure S35: UV-Vis spectra of association complexes 4 and 5 in DMSO; Figure S36: HPLC-UV chromatogram of compound **2**; Figure S37: HPLC-UV chromatogram of compound **2**; Figure S38: 2D-Fingerprint of Hirshfeld surface of compounds **1**–**3** and percentage of intermolecular interaction contributions in the crystal; Table S1: NOESY correlations of compound **2** in DMSO-d_6_ and CDCl_3_ (700 MHz); Table S2: Differences in ^1^H and ^13^C chemical shifts of free compound **2** and in complex; Table S3: Differences in ^1^H and ^13^C chemical shifts of free BCD and in complex; Table S4: Cellular growth inhibition (%); Table S5: Inhibition of yeast α-glucosidase. Determination of half-maximal inhibitory concentration (IC_50_); Table S6: Crystal data and structure refinement for 1; Table S7: Atomic coordinates (×10^4^) and equivalent isotropic displacement parameters (Å^2^ × 10^3^) for 1: U(eq) is defined as one third of the trace of the orthogonalized U^ij^ tensor; Table S8: Bond lengths [Å] and angles [°] for 1; Table S9: Anisotropic displacement parameters (Å^2^ × 10^3^) for 1. The anisotropic displacement factor exponent takes the form: −2π^2^[ h^2^ a*2U^11^ + … + 2hka* b* U^12^]; Table S10: Hydrogen coordinates (×10^4^) and isotropic displacement parameters (Å^2^ × 10^3^) for 1; Table S11: Crystal data and structure refinement for 2; Table S12. Atomic coordinates (×10^4^) and equivalent isotropic displacement parameters (Å^2^ × 10^3^) for 2. U(eq) is defined as one third of the trace of the orthogonalized U^ij^ tensor; Table S13: Bond lengths [Å] and angles [°] for 2; Table S14: Anisotropic displacement parameters (Å^2^ × 10^3^) for 2. The anisotropic displacement factor exponent takes the form: −2π^2^[ h^2^ a*2U^11^ + … + 2 h k a* b* U^12^]; Table S15: Hydrogen coordinates (×10^4^) and isotropic displacement parameters (Å^2^ × 10^3^) for 2; Table S16: Hydrogen bonds for 1 [Å and °]; Table S17: Crystal data and structure refinement for 3: Table S18: Atomic coordinates (×10^4^) and equivalent isotropic displacement parameters (Å^2^ × 10^3^) for 3. U(eq) is defined as one third of the trace of the orthogonalized U^ij^ tensor; Table S19: Bond lengths [Å] and angles [°] for 3; Table S20: Anisotropic displacement parameters (Å^2^ × 10^3^) for 3. The anisotropic displacement factor exponent takes the form: −2π^2^[ h^2^ a*2U^11^ + … + 2 h k a* b* U^12^]; Table S21: Hydrogen coordinates (×10^4^) and isotropic displacement parameters (Å^2^ × 10^3^) for 3; Graphic S1. TGA and DSC thermograms of compounds **2**, **4** and dry BCD [84,85,86]; Graphic S2. TGA and DSC thermograms of compounds **3**, **5** and dry BCD. The crystallographic data of compounds **1**–**3** were deposited in the CCDC with numbers 2483475–2483477, https://www.ccdc.cam.ac.uk/ (accessed on 28 August 2025).

## Abbreviations

The following abbreviations are used in this manuscript:

NOESY	Nuclear Overhauser Effect Spectroscopy
NMR	Nuclear magnetic resonance
FT-IR	Fourier Transform Infrared spectroscopy
UV-Vis	Ultraviolet–Visible spectroscopy
BDC	β-Cyclodextrin
ORTEP	Oak Ridge Thermal Ellipsoid Plot
RMS	Root Mean Square
CCDC	Cambridge Crystallographic Data Centre
MS	Mass Spectrometry
