# Supplementary material for: Synthesis of First Copper Metal Complex of C=C Extended Curcuminoid Analogue: Structure, β-Cyclodextrin Association, and Biological Properties
Source: Molecules. 2025 Oct 1;30(19):3943. doi: 10.3390/molecules30193943 (PMC12526022; doi:10.3390/molecules30193943)
Supplement: Supplementary file 1 [file molecules-30-03943-s001.zip › molecules-3883884-supplementary.pdf]

Supplementary Information

Synthesis of First Copper Metal Complex of C=C Extended Curcuminoid Analogue: Structure, β-Cyclodextrin Association, and Biological Properties

Rosario Tavera-Hernández *, Rubén Sánchez-Obregón, Marco A. Obregón-Mendoza *, Antonio Nieto-Camacho, María Teresa Ramírez-Apan, Leidys L. Pérez-González and Raúl G. Enríquez *

Instituto de Química, Universidad Nacional Autónoma de México, Circuito Exterior, Ciudad Universitaria, Mexico City 04510, Mexico; rubens@unam.mx (R.S.-O.); camanico2015@yahoo.com (A.N.-C.); mtrapan@yahoo.com.mx (M.T.R.-A.); leidyslaura92@gmail.com (L.L.P.-G.)

* Correspondence: rosario.tavera@gmail.com (R.T.-H.); marco.obregon@zaragoza.unam.mx (M.A.O.-M.); enriquezhabib@gmail.com or habib@unam.mx (R.G.E.);
Tel.: +52-55-55562-24404 (R.T.-H. & M.A.O.-M. & R.G.E.)

Index

Figure S1. 1H-NMR spectrum of compound 1 (DMSO-d6, 700 MHz) 4
Figure S2. ^{13}C-NMR spectrum of compound **1** (DMSO-d_6, 175 MHz) 4
Figure S3. COSY spectrum of compound 1 (DMSO-d_6) 5
Figure S4. HSQC spectrum of compound 1 (DMSO-d_6) 5
Figure S5. HMBC spectrum of compound 1 (DMSO-d_6) 6
Figure S6. ^{1}H-NMR spectrum of compound **2** (DMSO-d_6, 700 MHz) 6
Figure S7. ^{13}C-NMR spectrum of compound **2** (DMSO-d_6, 175 MHz) 7
Figure S8. COSY spectrum of compound 2 (DMSO-d_6) 7
Figure S9. HSQC spectrum of compound 2 (DMSO-d_6) 8
Figure S10. HMBC spectrum of compound 2 (DMSO-d_6) 8
Figure S11. NOESY spectrum of compound **2** (DMSO-d_6, 700 MHz) 9
Figure S12. ^{1}H-NMR spectrum of compound **2** ($CDCl_3$, 700 MHz) 9
Figure S13. ^{13}C-NMR spectrum of compound **2** ($CDCl_3$, 175 MHz) 10
Figure S14. COSY spectrum of compound **2** ($CDCl_3$) 10
Figure S15. HSQC spectrum of compound **2** ($CDCl_3$) 11
Figure S16. HMBC spectrum of compound **2** ($CDCl_3$) 11
Figure S17. NOESY spectrum of compound **2** ($CDCl_3$, 700 MHz) 12
Figure S18. ^{1}H-NMR spectrum of association complex **4** (DMSO-d_6, 700 MHz) 12
Figure S19. ^{13}C-NMR spectrum of association complex **4** (DMSO-d_6, 175 MHz) 13
Figure S20. NOESY spectrum of association complex **4** (DMSO-d_6, 700 MHz) 13
Figure S21. NOESY spectrum expansion of association complex **4** (DMSO-d_6, 700 MHz) 14
Figure S22. Electron paramagnetic resonance spectrum of compound **3** (THF) 14
Figure S23. Mass spectrometry spectrum of compound **1** (ESI-) 15
Figure S24. Mass spectrometry spectrum of compound **2** (ESI+) 16
Figure S25. Mass spectrometry spectrum of compound **3** (MALDI-TOF) 17
Figure S26. Mass spectrometry spectrum of compound **3**, expansion (MALDI-TOF) 17
Figure S27. Infrared spectrum of compound **1** (ATR) 18
Figure S28. Infrared spectrum of compound **2** (ATR) 18
Figure S29. Infrared spectrum of compound **3** (ATR) 19
Figure S30. Infrared spectrum of association complex **4** (ATR) 19
Figure S31. Infrared spectrum of association complex **5**(ATR) 20
Figure S32. Infrared spectrum of β-cyclodextrin (ATR) 20
Figure S33. UV-Vis spectrum of compound **2** (DMSO) 21
Figure S34. UV-Vis spectrum of compound **3** (DMSO) 21
Figure S35. UV-Vis spectra of association complexes **4** and **5** in DMSO 22
Figure S36. HPLC-UV chromatogram of compound **2** 22
Figure S37. HPLC-UV chromatogram of compound **2** 23
Figure S38. 2D-Fingerprint of Hirshfeld surface of compounds **1**-**3** and percentage of intermolecular interaction contributions in the crystal 24
Table S1. NOESY correlations of compound **2** in DMSO-d_6 and $CDCl_3$ (700 MHz) 25
Table S2. Differences in ^{1}H and ^{13}C chemical shifts of free compound **2** and in complex ($\Delta\delta = \delta_{complex} - \delta_{free}$) 25
Table S3. Differences in ^{1}H and ^{13}C chemical shifts of free BCD and in complex ($\Delta\delta = \delta_{complex} - \delta_{free}$) 25
Table S4. Cellular growth inhibition (%) 26
Table S5. Inhibition of yeast α-glucosidase. Determination of half-maximal inhibitory concentration (IC_{50}) 27
Table S6. Crystal data and structure refinement for **1** 28
Table S7. Atomic coordinates (x 10^4) and equivalent isotropic displacement parameters (Å^{2}x 10^3) for **1**. U(eq) is defined as one third of the trace of the orthogonalized U^{ij} tensor 29
Table S8. Bond lengths [Å] and angles [°] for **1**. 30

Table S9. Anisotropic displacement parameters ($Å^2$x 10^3) for **1**. The anisotropic displacement factor exponent takes the form: $-2p^2[h^2 a^{*2}U^{11} + ... + 2 h k a^* b^* U^{12}]$ 32
Table S 10. Hydrogen coordinates (x 10^4) and isotropic displacement parameters ($Å^2$x 10^3) for **1**........ 33
Table S11. Crystal data and structure refinement for **2** 34
Table S12. Atomic coordinates (x 10^4) and equivalent isotropic displacement parameters ($Å^2$x 10^3) for **2**. U(eq) is defined as one third of the trace of the orthogonalized U^{ij} tensor........ 35
Table S13. Bond lengths [Å] and angles [°] for **2**. 36
Table S14. Anisotropic displacement parameters ($Å^2$x 10^3) for **2**. The anisotropic displacement factor exponent takes the form: $-2p^2[h^2 a^{*2}U^{11} + ... + 2 h k a^* b^* U^{12}]$ 38
Table S15. Hydrogen coordinates (x 10^4) and isotropic displacement parameters ($Å^2$x 10^3) for **2**........ 39
Table S16. Hydrogen bonds for 1 [Å and °]. 39
Table S17. Crystal data and structure refinement for 3. 40
Table S18. Atomic coordinates (x 10^4) and equivalent isotropic displacement parameters ($Å^2$x 10^3) for **3**. U(eq) is defined as one third of the trace of the orthogonalized U^{ij} tensor........ 41
Table S19. Bond lengths [Å] and angles [°] for **3**. 42
Table S20. Anisotropic displacement parameters ($Å^2$x 10^3) for **3**. The anisotropic displacement factor exponent takes the form: $-2\pi^2[h^2 a^{*2}U^{11} + ... + 2 h k a^* b^* U^{12}]$ 44
Table S21. Hydrogen coordinates (x 10^4) and isotropic displacement parameters ($Å^2$x 10^3) for **3**........ 45
Graphic S1. TGA and DSC thermograms of compounds **2**, **4** and dry BCD........ 46
Graphic S2. TGA and DSC thermograms of compounds **3**, **5** and dry BCD 47

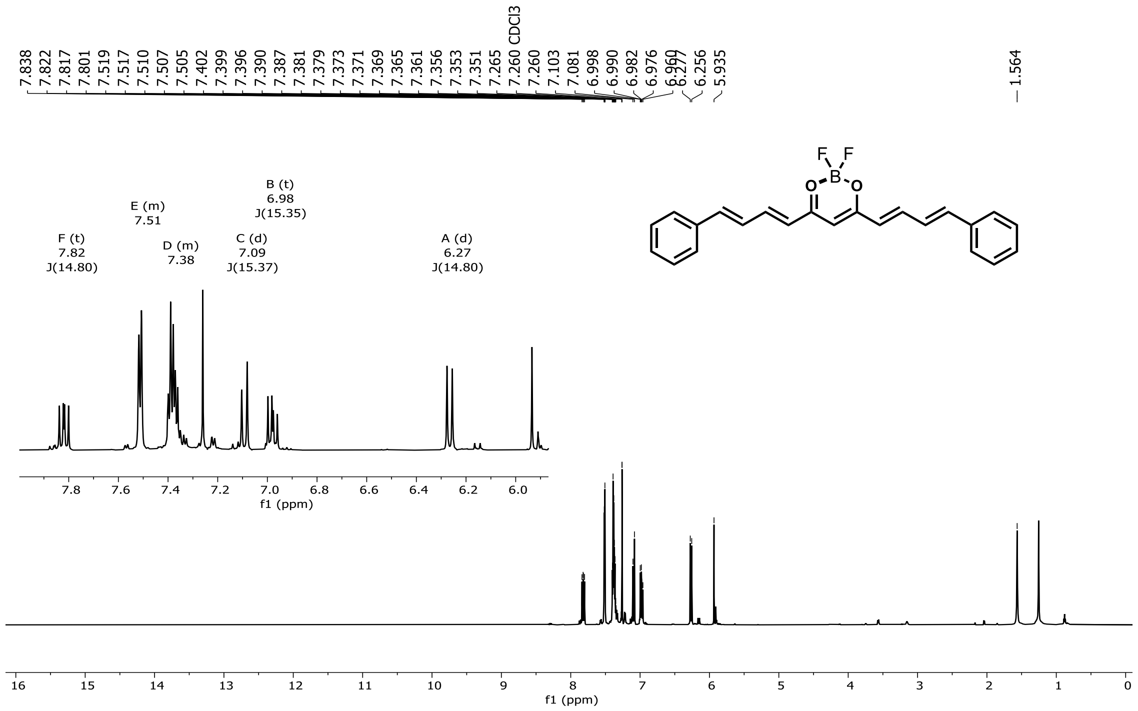

Figure S1. 1H-NMR spectrum of compound 1 (DMSO-d6, 700 MHz)

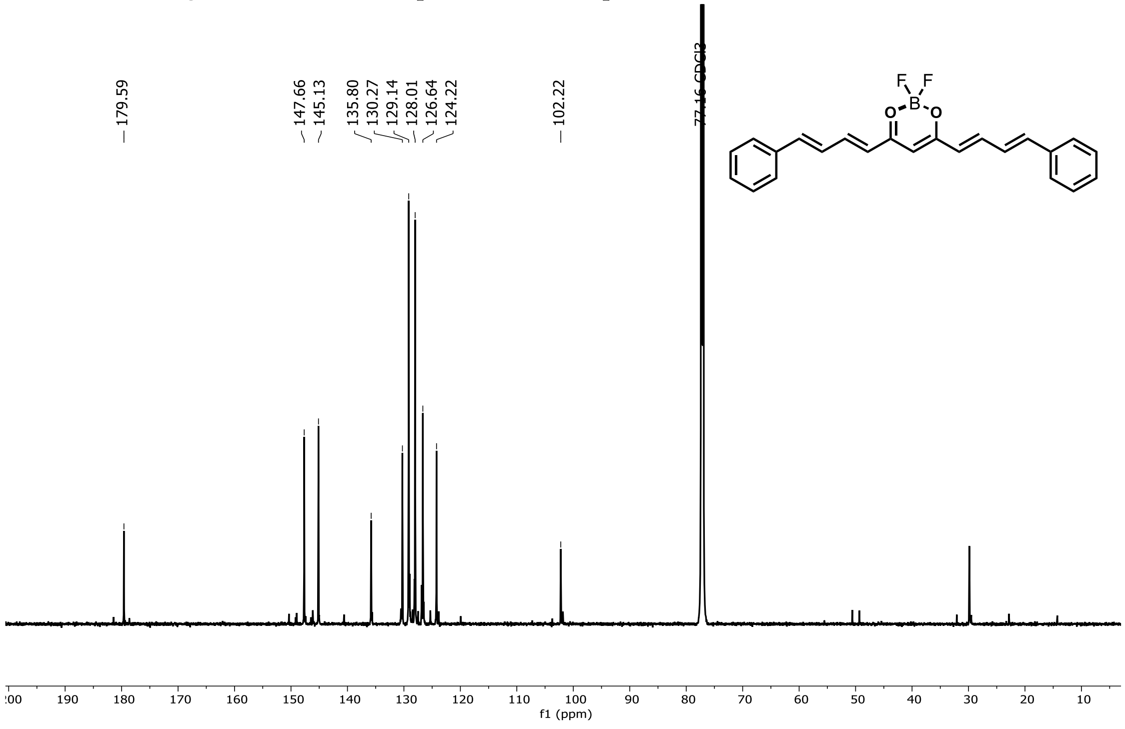

Figure S2. ^{13}C-NMR spectrum of compound **1** (DMSO-d$_6$, 175 MHz)

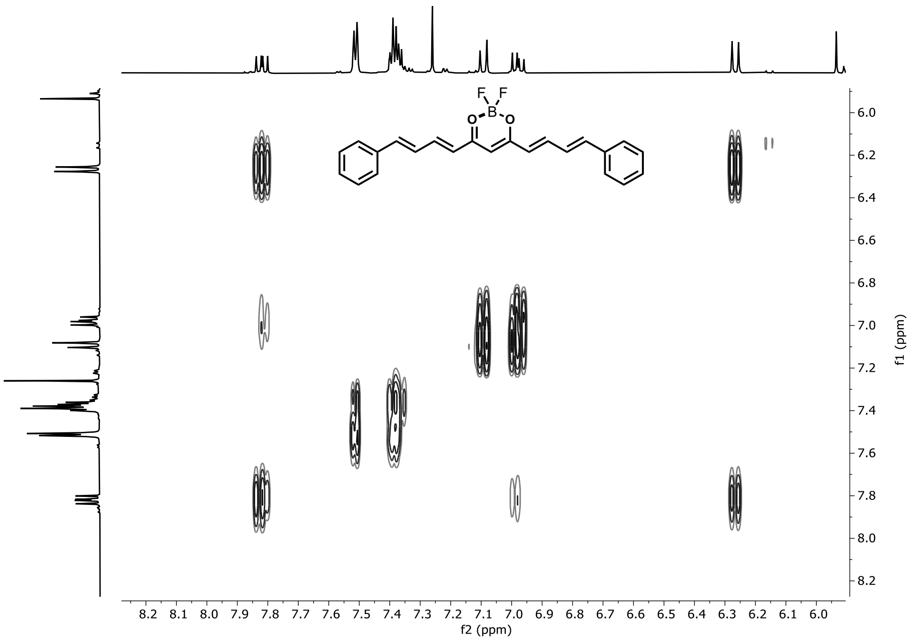

Figure S3. COSY spectrum of compound 1 (DMSO-d$_6$)

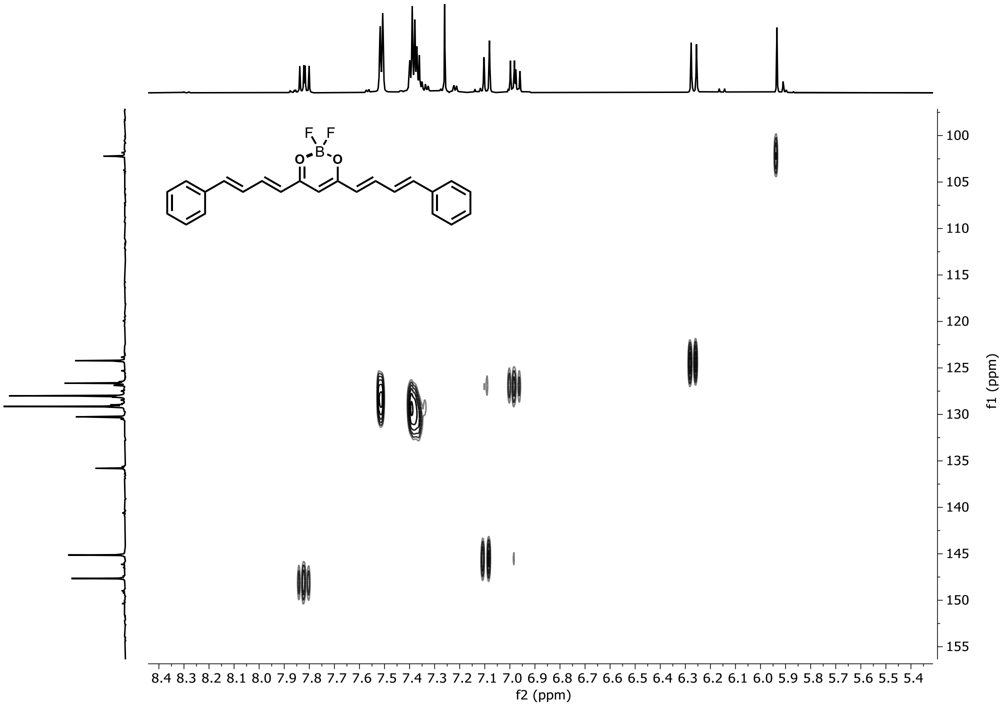

Figure S4. HSQC spectrum of compound 1 (DMSO-d$_6$)

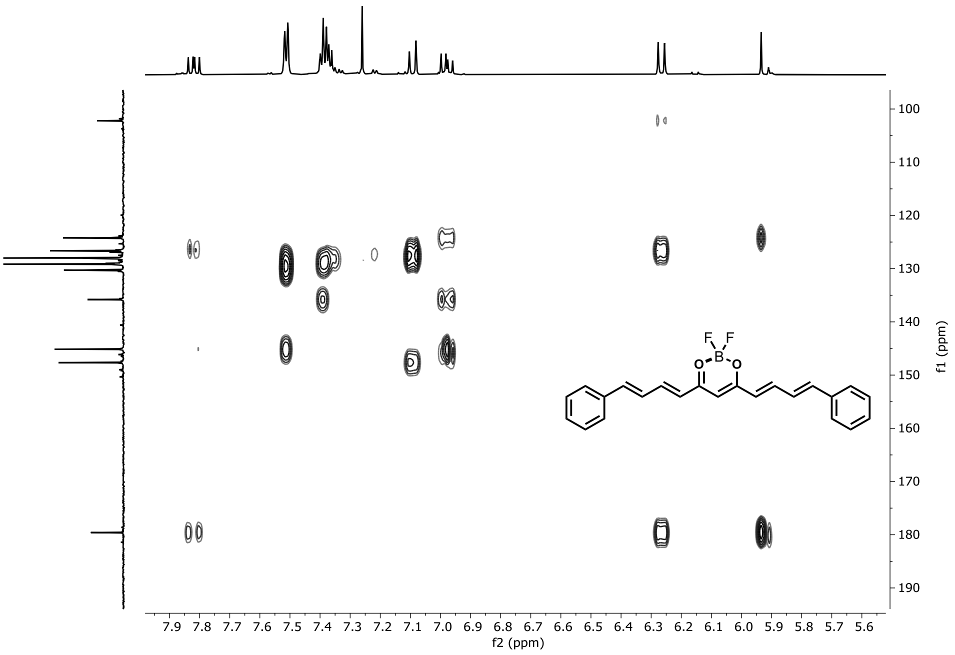

Figure S5. HMBC spectrum of compound 1 (DMSO-d$_6$)

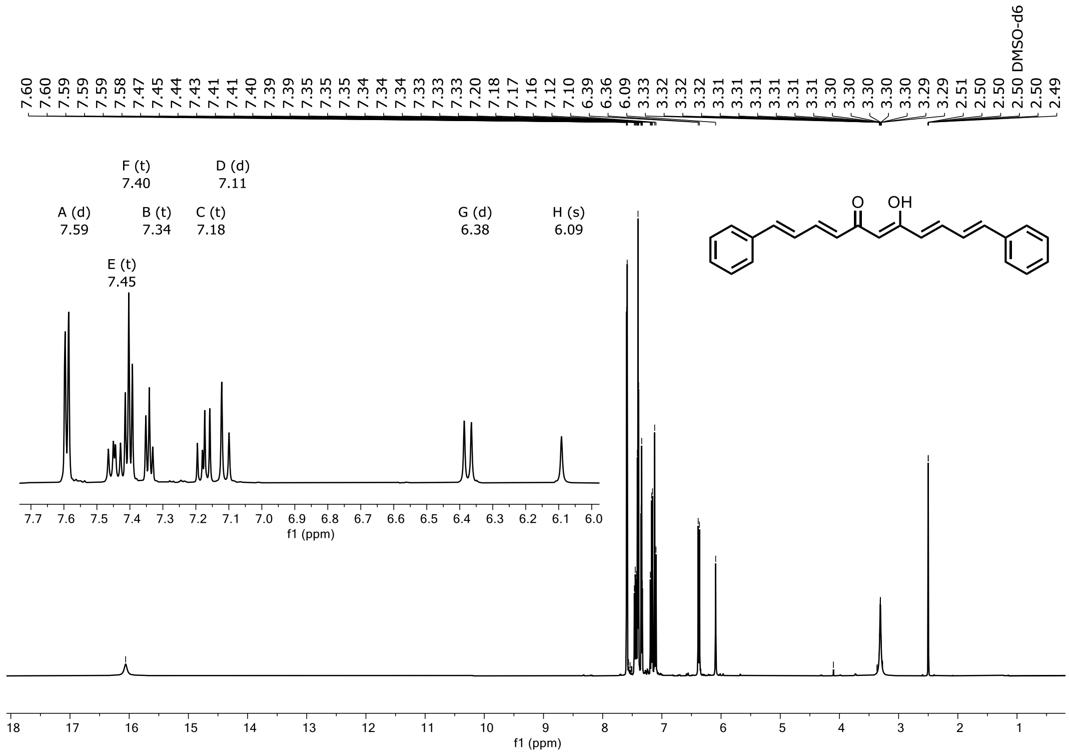

Figure S6. ^{1}H-NMR spectrum of compound **2** (DMSO-d$_6$, 700 MHz)

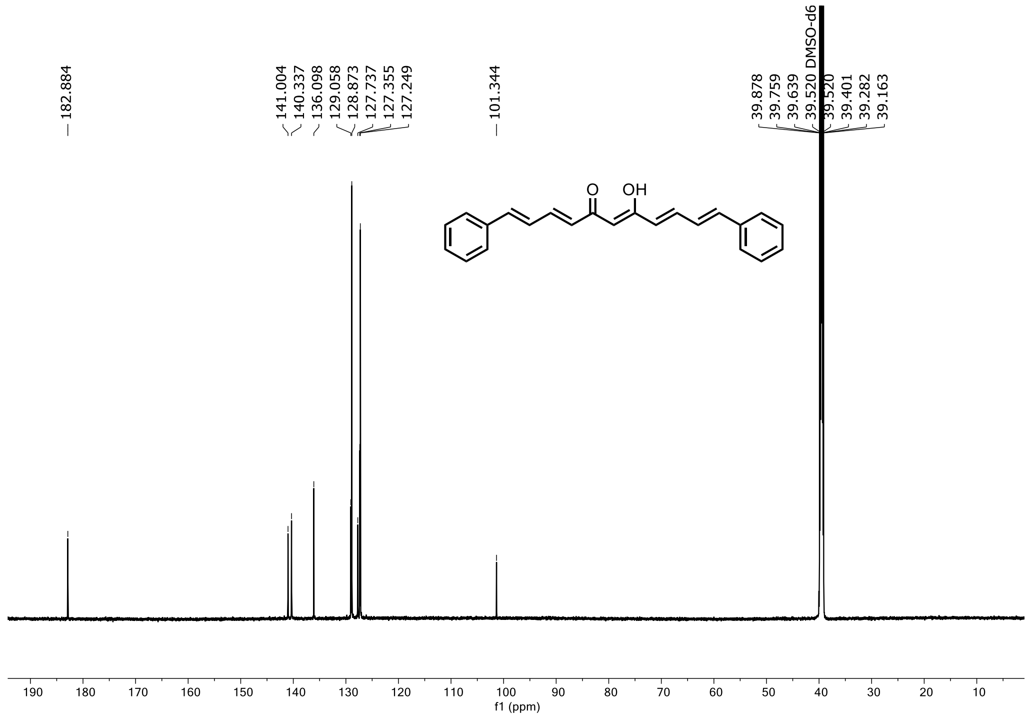

Figure S7. ^{13}C-NMR spectrum of compound **2** (DMSO-d$_6$, 175 MHz)

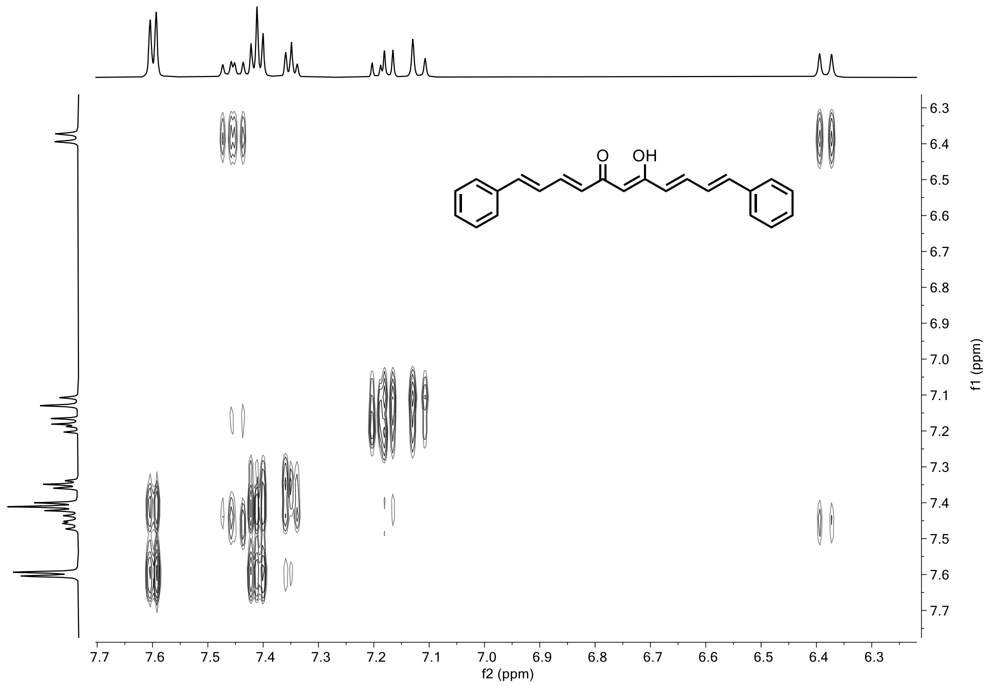

Figure S8. COSY spectrum of compound 2 (DMSO-d$_6$)

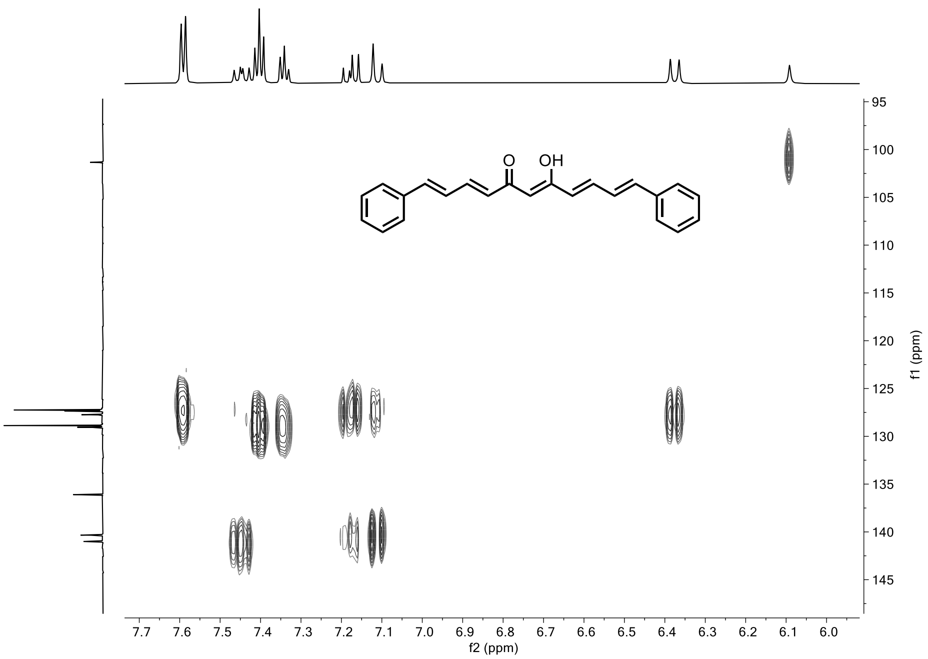

Figure S9. HSQC spectrum of compound 2 (DMSO-d_6)

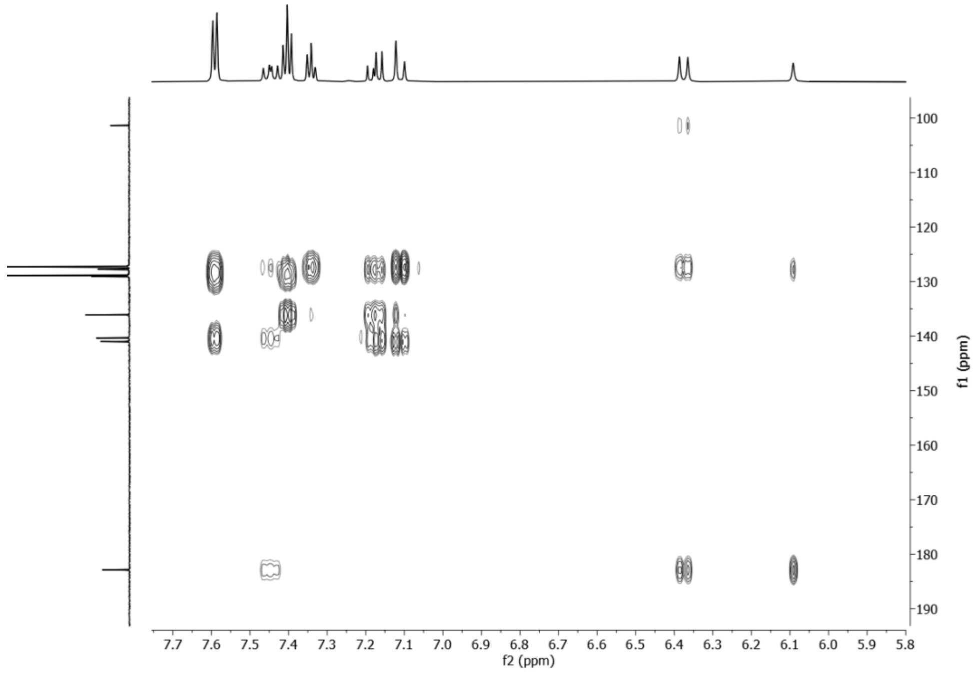

Figure S10. HMBC spectrum of compound 2 (DMSO-d_6)

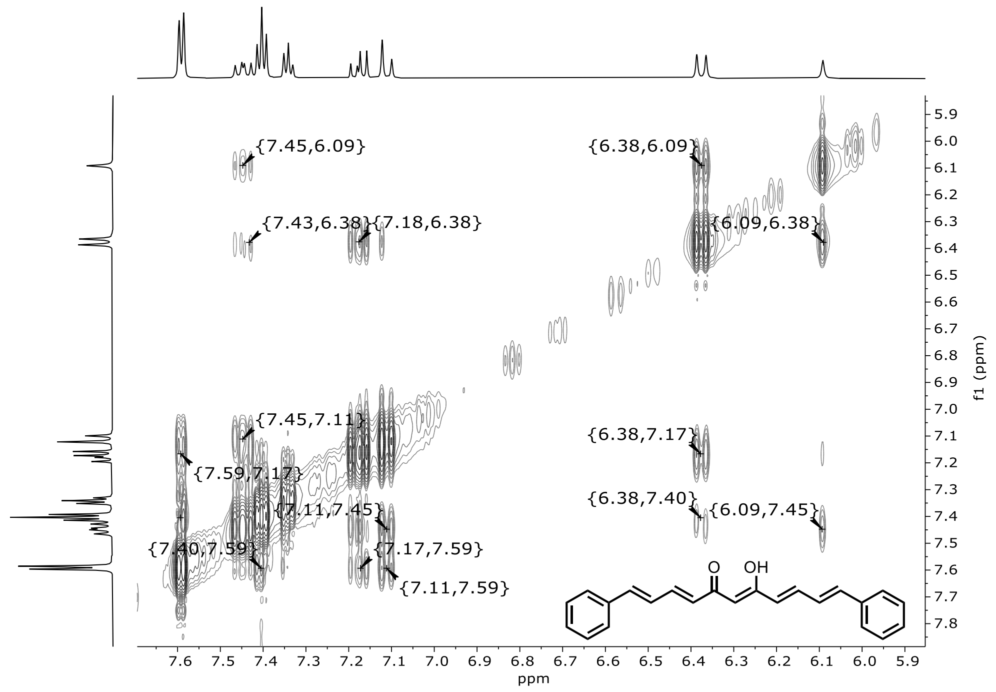

Figure S11. NOESY spectrum of compound **2** (DMSO-d6, 700 MHz)

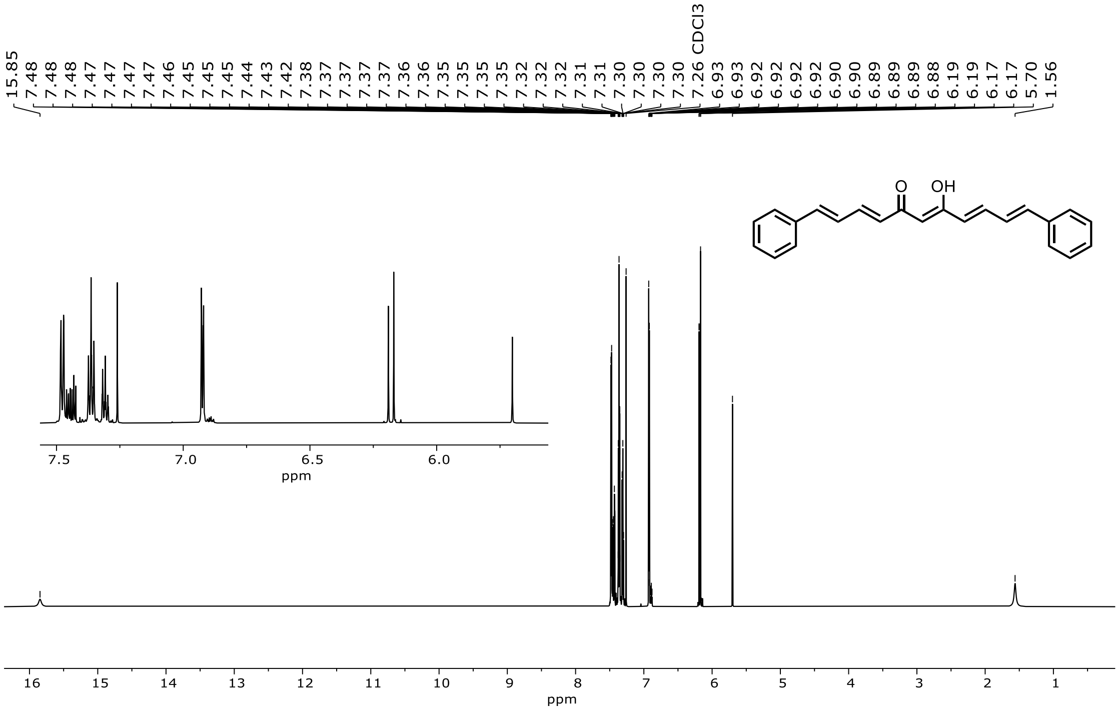

Figure S12. ^{1}H-NMR spectrum of compound **2** ($CDCl_3$, 700 MHz)

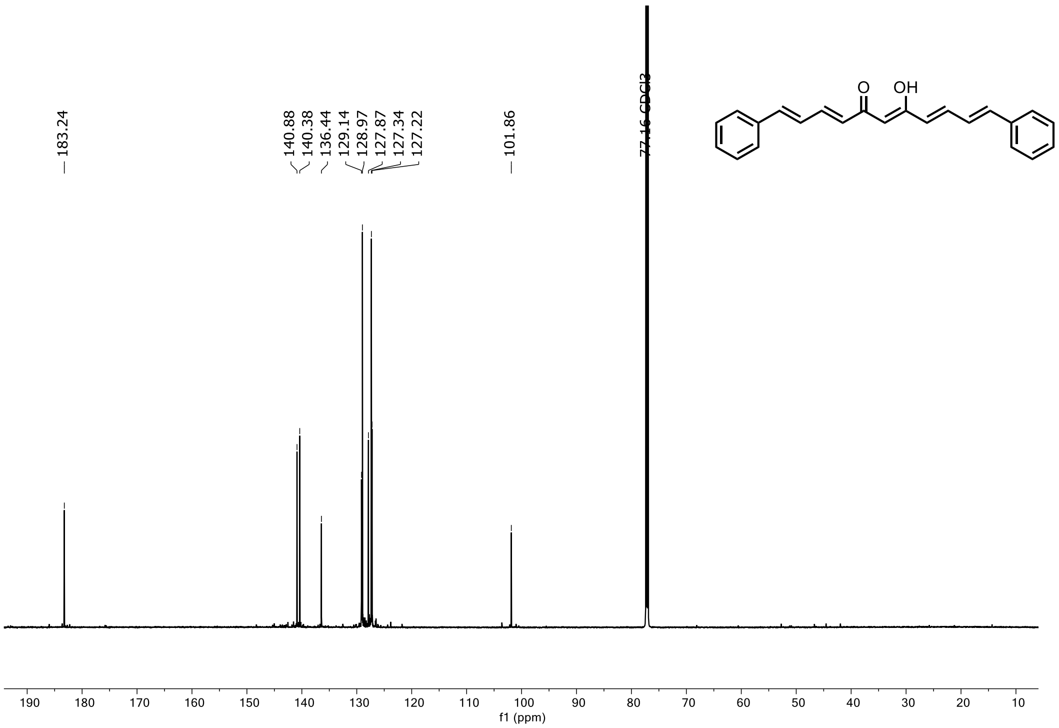

Figure S13. ^{13}C-NMR spectrum of compound **2** ($CDCl_3$, 175 MHz)

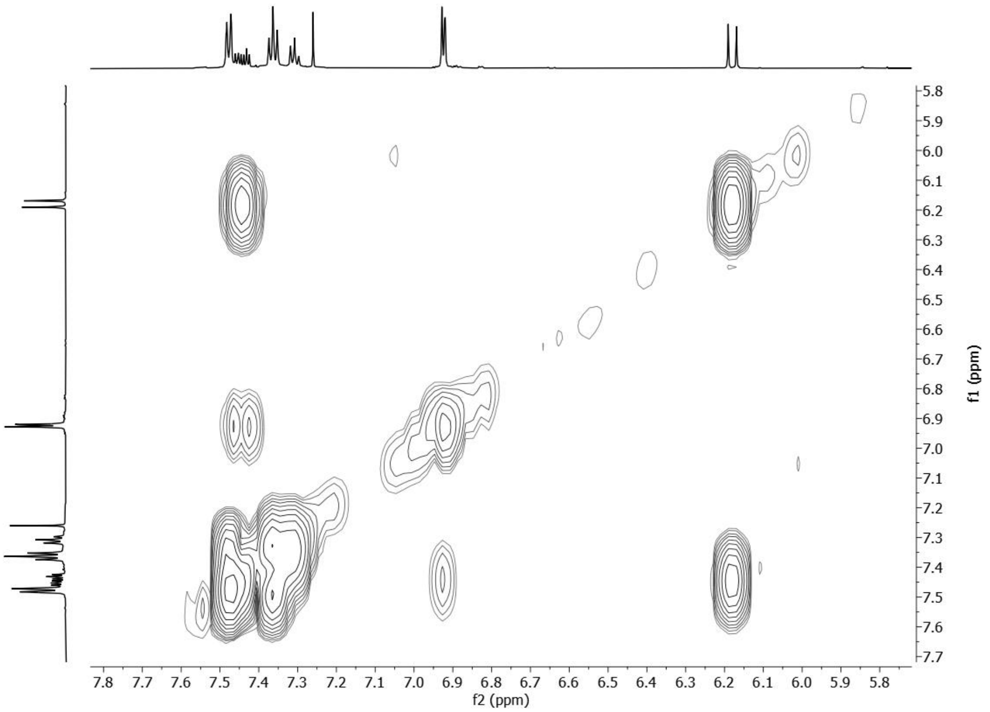

Figure S14. COSY spectrum of compound **2** ($CDCl_3$)

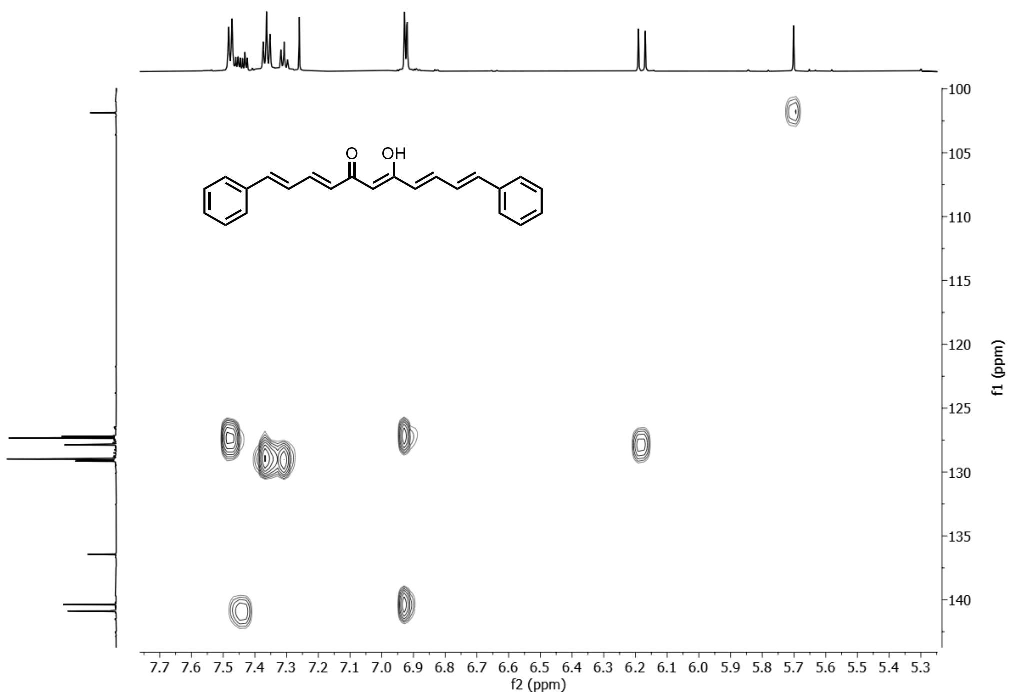

Figure S15. HSQC spectrum of compound **2** ($CDCl_3$)

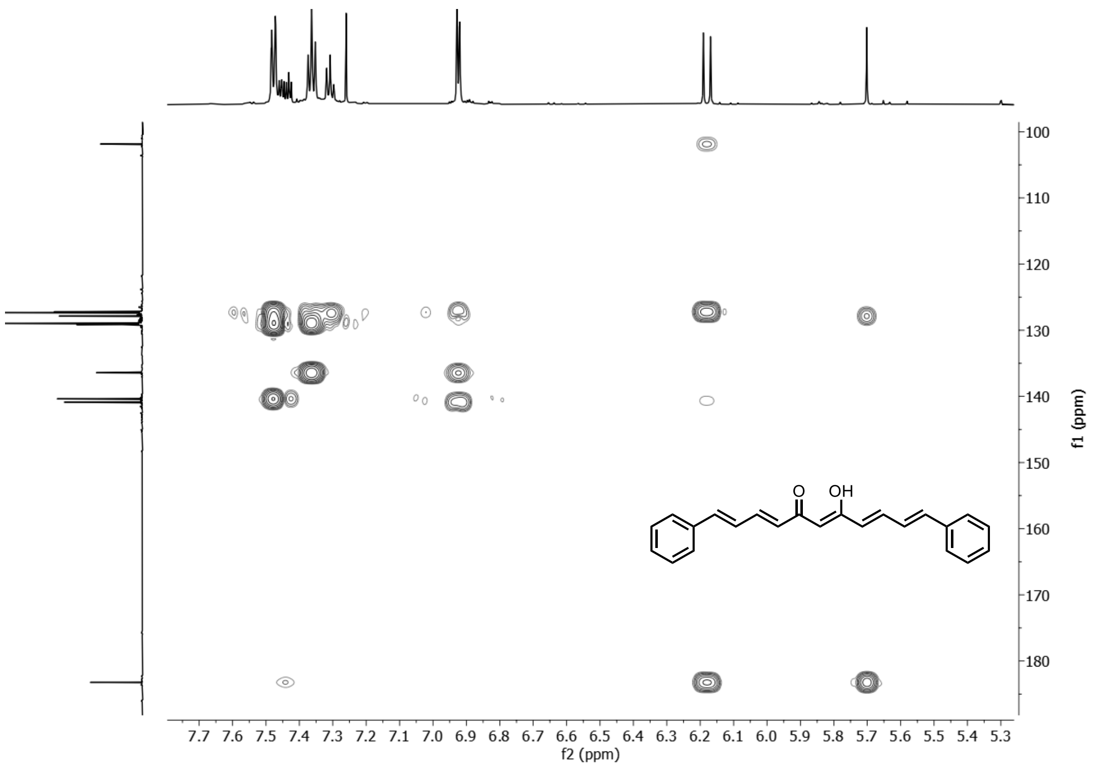

Figure S16. HMBC spectrum of compound **2** ($CDCl_3$)

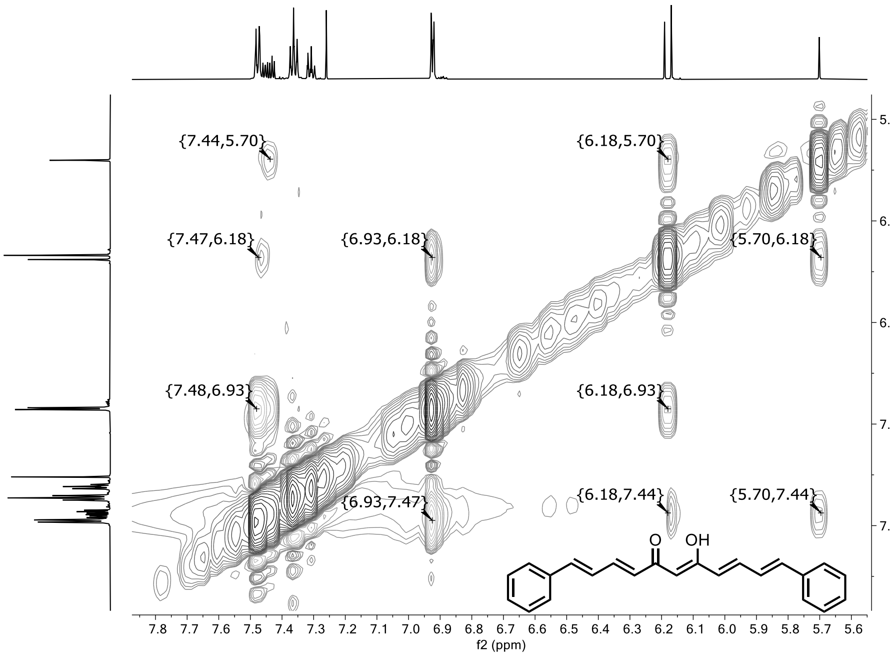

Figure S17. NOESY spectrum of compound **2** ($CDCl_3$, 700 MHz)

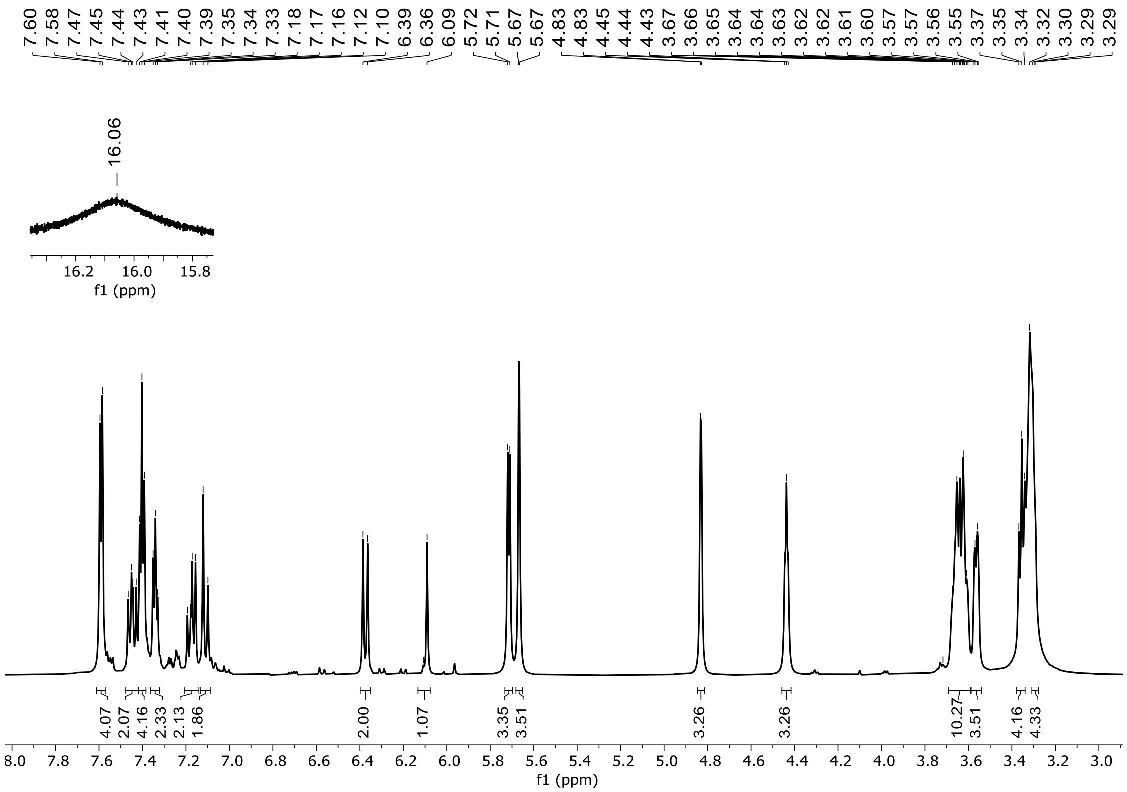

Figure S18. ^{1}H-NMR spectrum of association complex **4** (DMSO-d_6, 700 MHz)

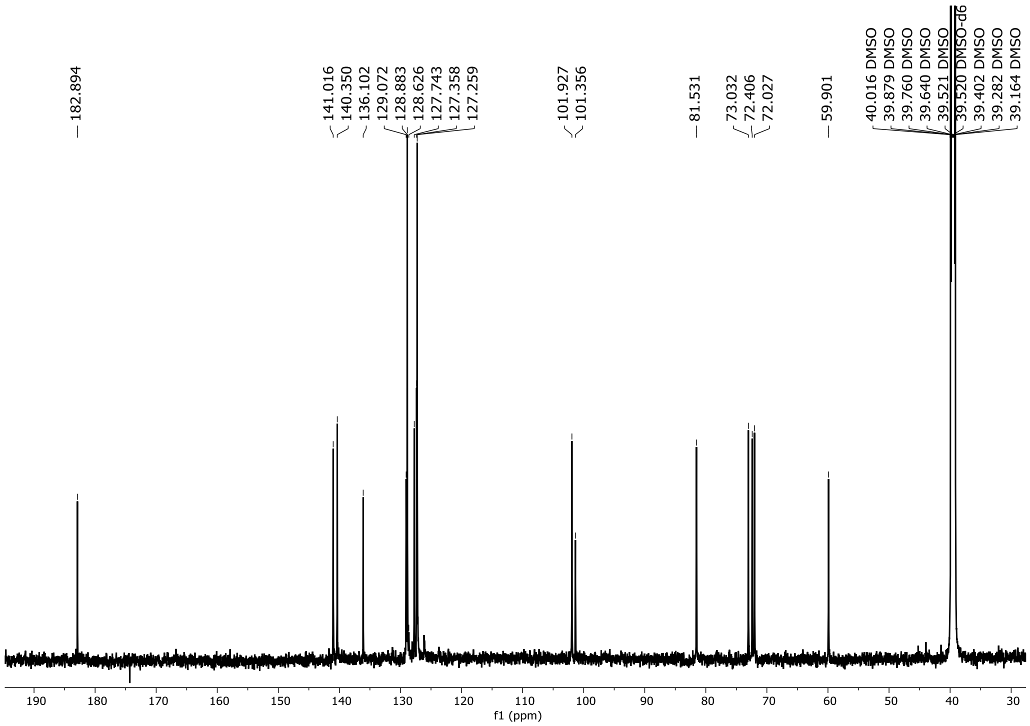

Figure S19. ^{13}C-NMR spectrum of association complex **4** (DMSO-d_6, 175 MHz)

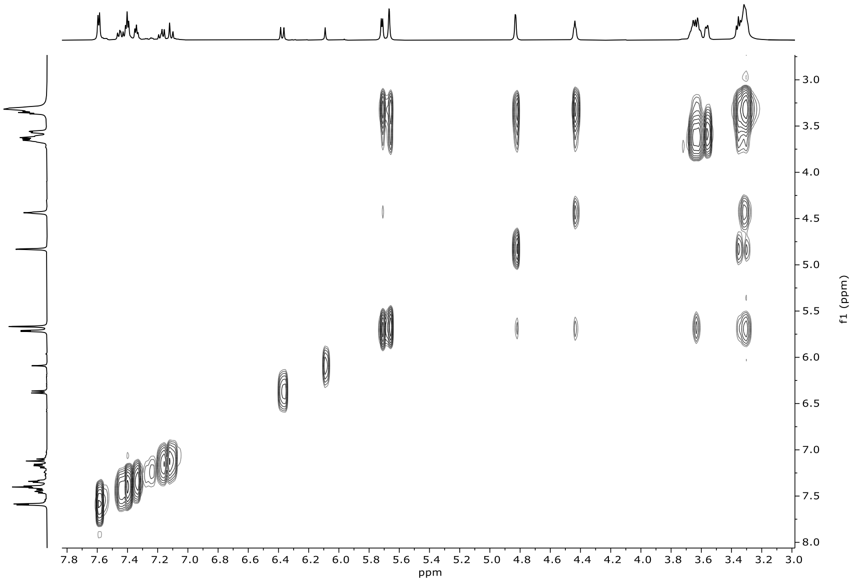

Figure S20. NOESY spectrum of association complex **4** (DMSO-d_6, 700 MHz)

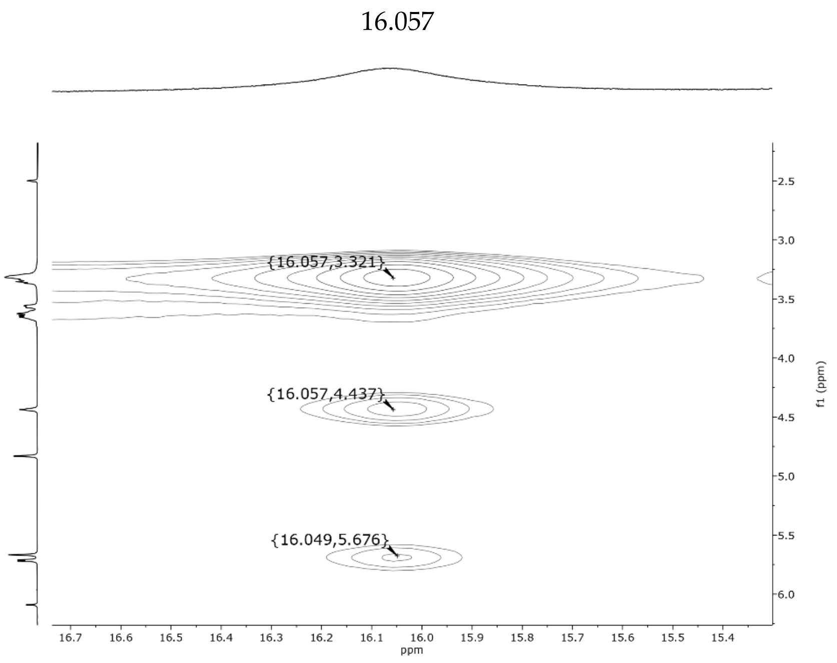

Figure S21. NOESY spectrum expansion of association complex **4** (DMSO-d6, 700 MHz)

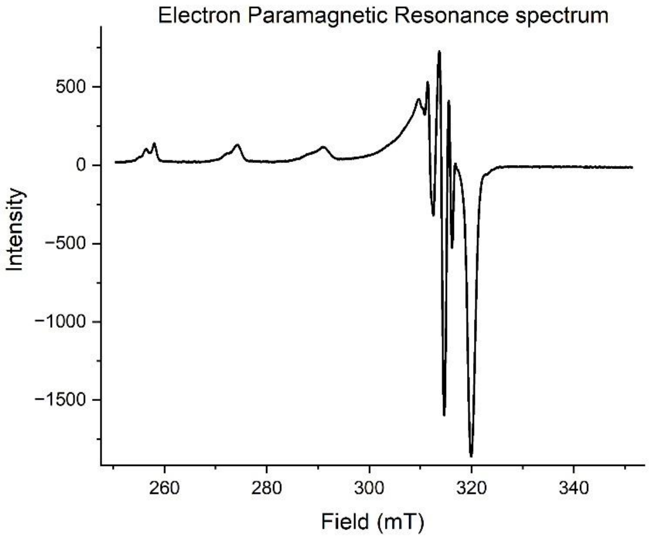

Figure S22. Electron paramagnetic resonance spectrum of compound **3** (THF)

Sample Name	28942		
No de registro	CurCin BF2	Operator	Carmen Marquez
Descripción		Instrument	esquire6000
Archivo	D:\Data\ehr\ehr00364.d		

Acquisition Parameter

Ion Source Type	ESI			Alternating Ion Polarity	off
Mass Range Mode	Std/Normal	Scan Begin	50 m/z	Scan End	2000 m/z
Capillary Exit	111.3 Volt	Skim 1	40.0 Volt	Trap Drive	51.1
Accumulation Time	18044 µs	Averages	5 Spectra	Auto MS/MS	off

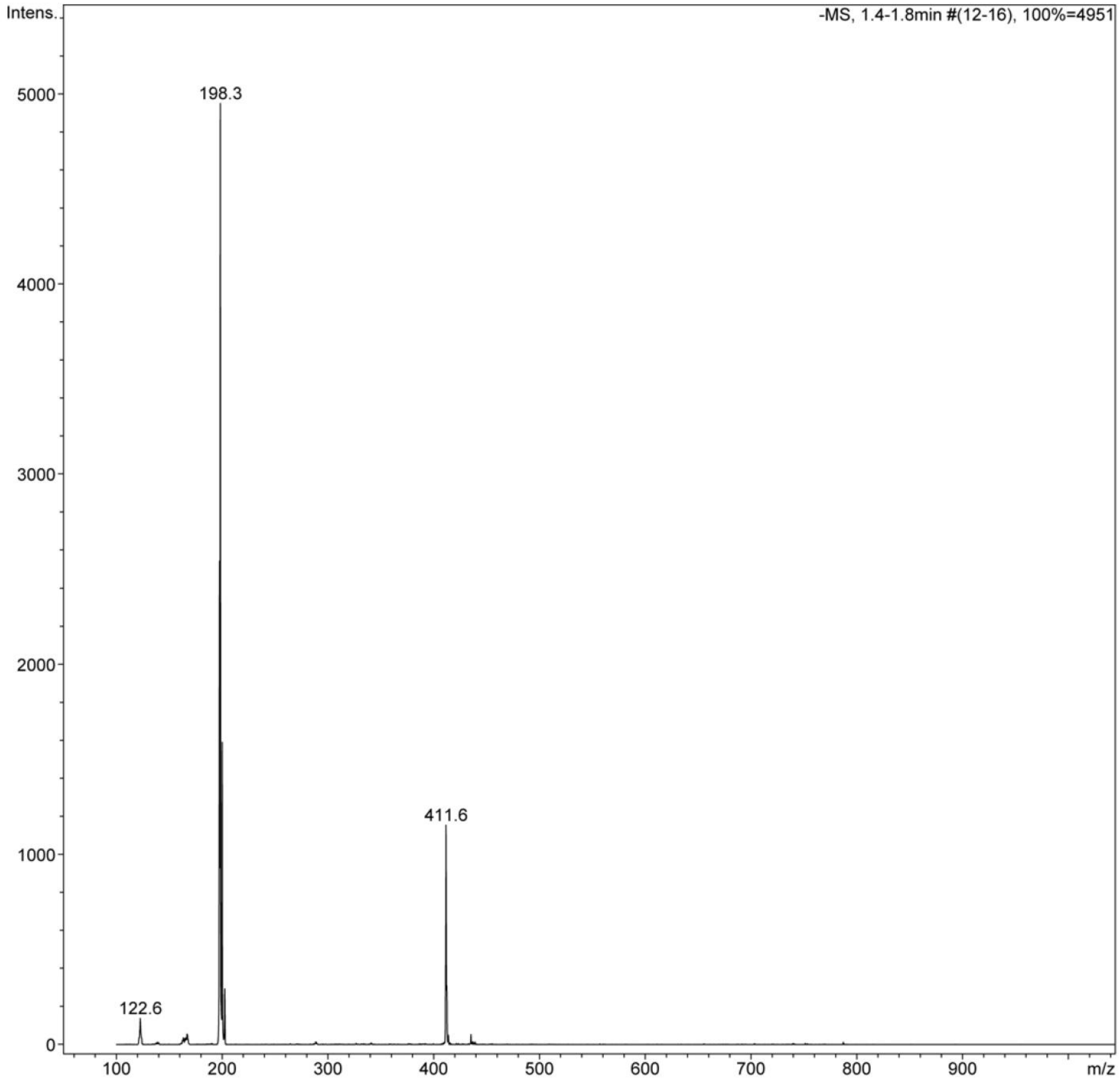

Figure S23. Mass spectrometry spectrum of compound **1** (ESI-)

Sample Name	28943		
No de registro	CurCin	Operator	Carmen Marquez
Descripción		Instrument	esquire6000
Archivo	D:\Data\ehr\ehr00363.d		

Acquisition Parameter

Ion Source Type	ESI			Alternating Ion Polarity	off
Mass Range Mode	Std/Normal	Scan Begin	70 m/z	Scan End	2000 m/z
Capillary Exit	111.3 Volt	Skim 1	40.0 Volt	Trap Drive	51.1
Accumulation Time	28875 µs	Averages	5 Spectra	Auto MS/MS	off

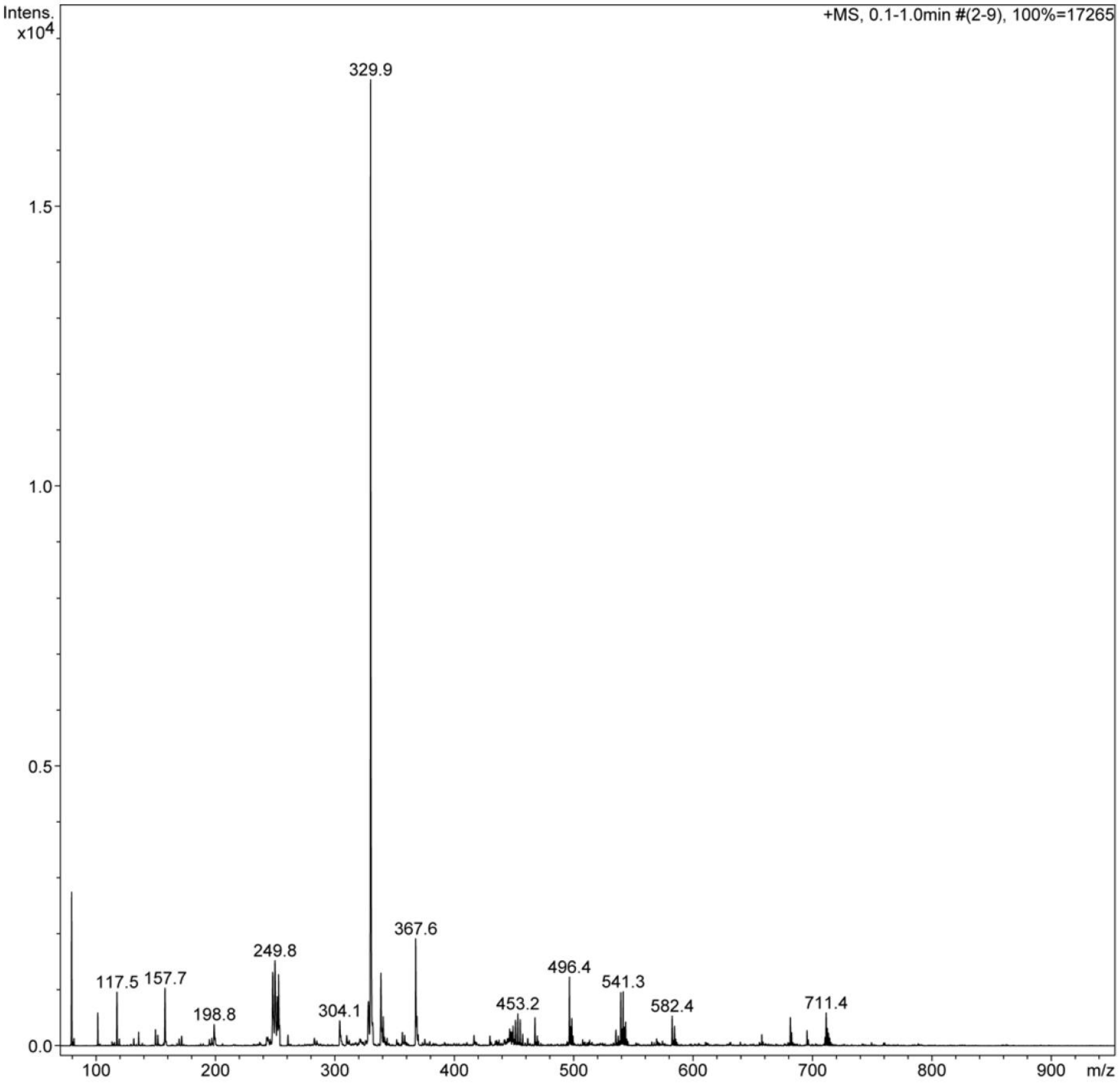

Figure S24. Mass spectrometry spectrum of compound **2** (ESI+)

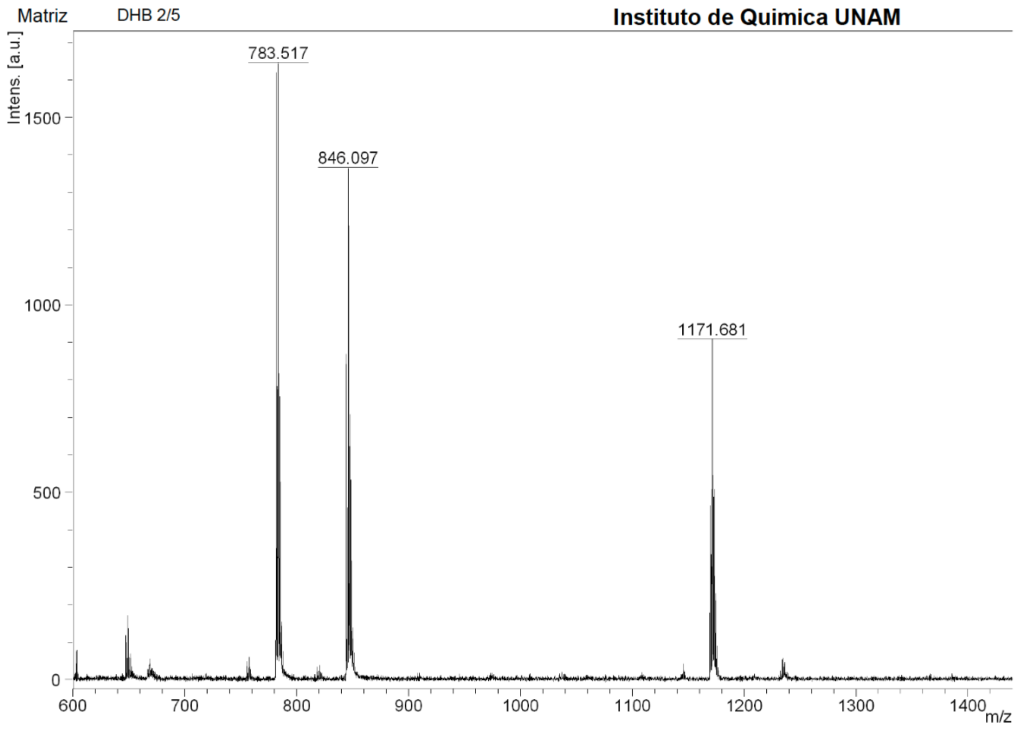

Figure S25. Mass spectrometry spectrum of compound **3** (MALDI-TOF)

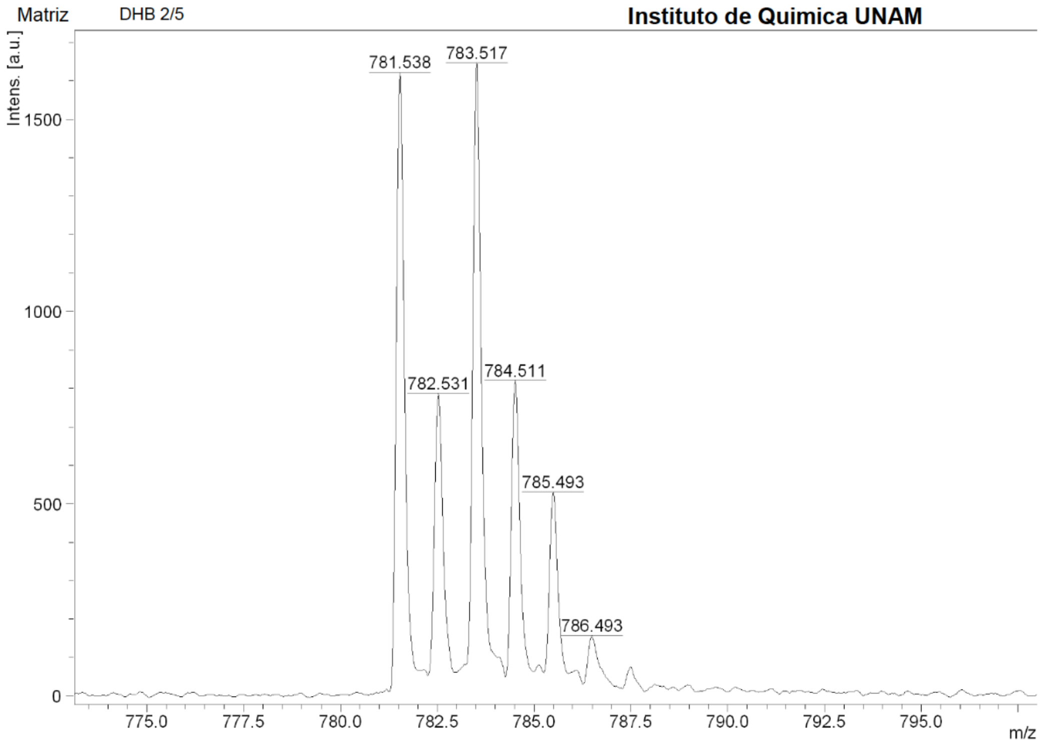

Figure S26. Mass spectrometry spectrum of compound **3**, expansion (MALDI-TOF)

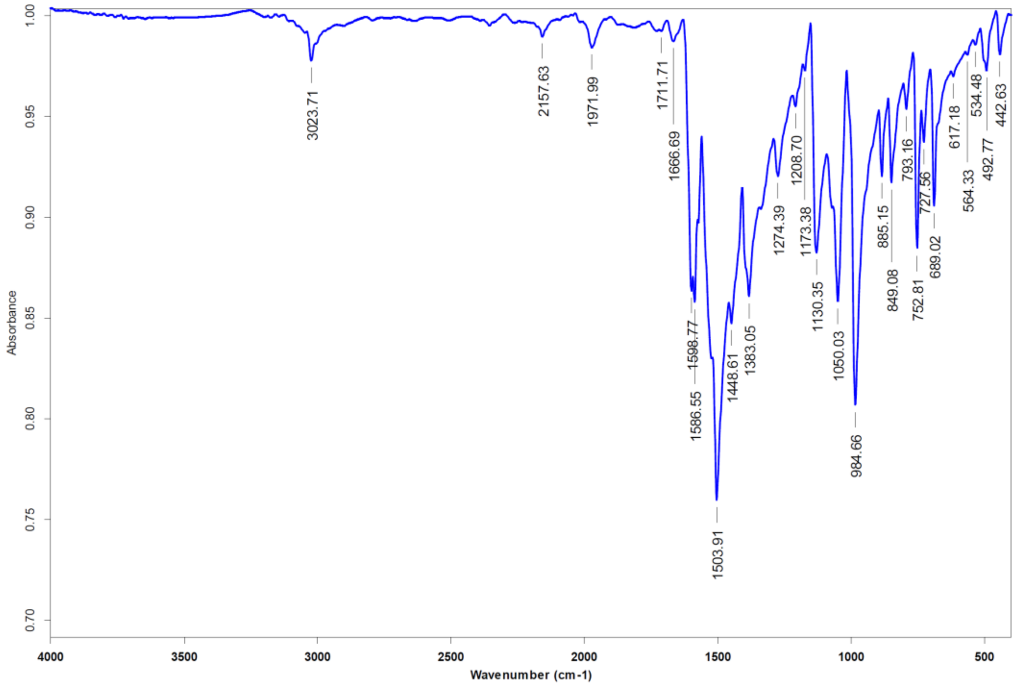

Figure S27. Infrared spectrum of compound **1** (ATR)

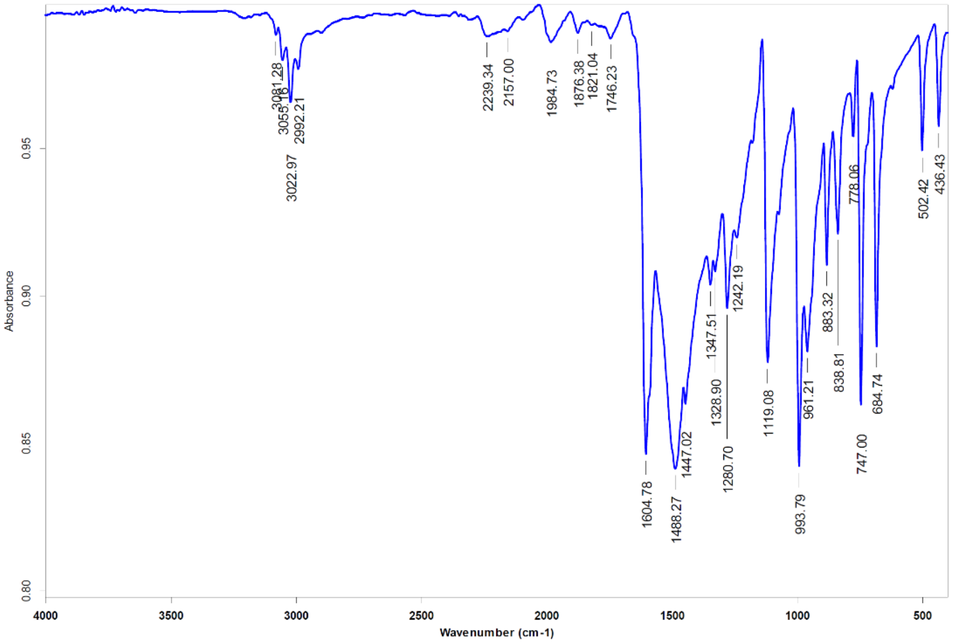

Figure S28. Infrared spectrum of compound **2** (ATR)

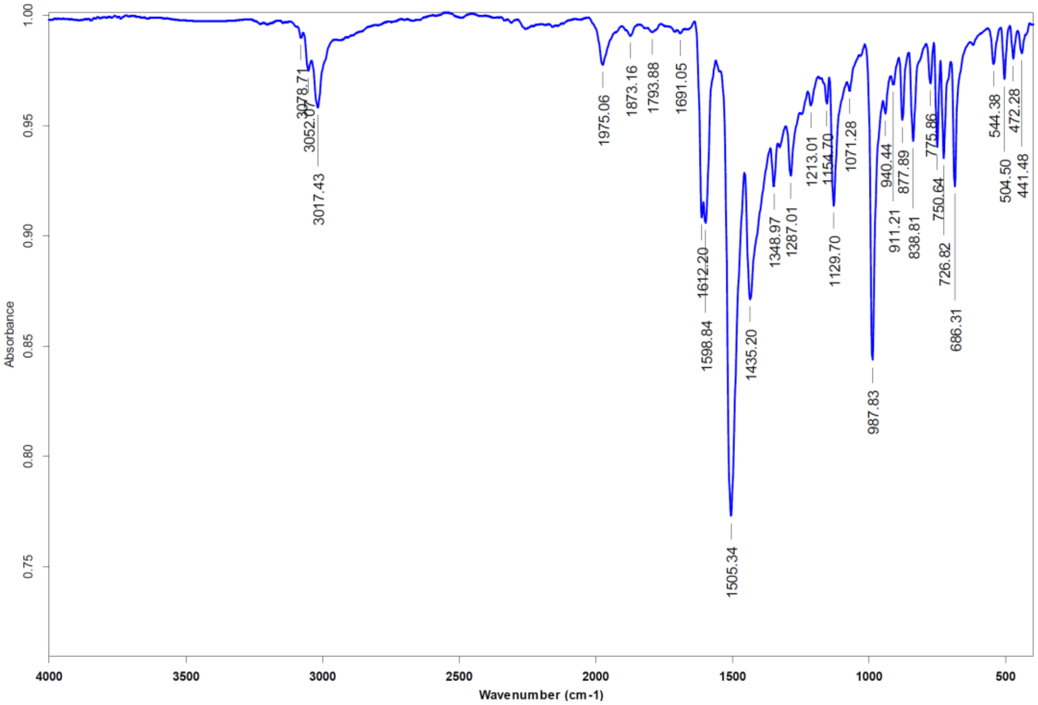

Figure S29. Infrared spectrum of compound **3** (ATR)

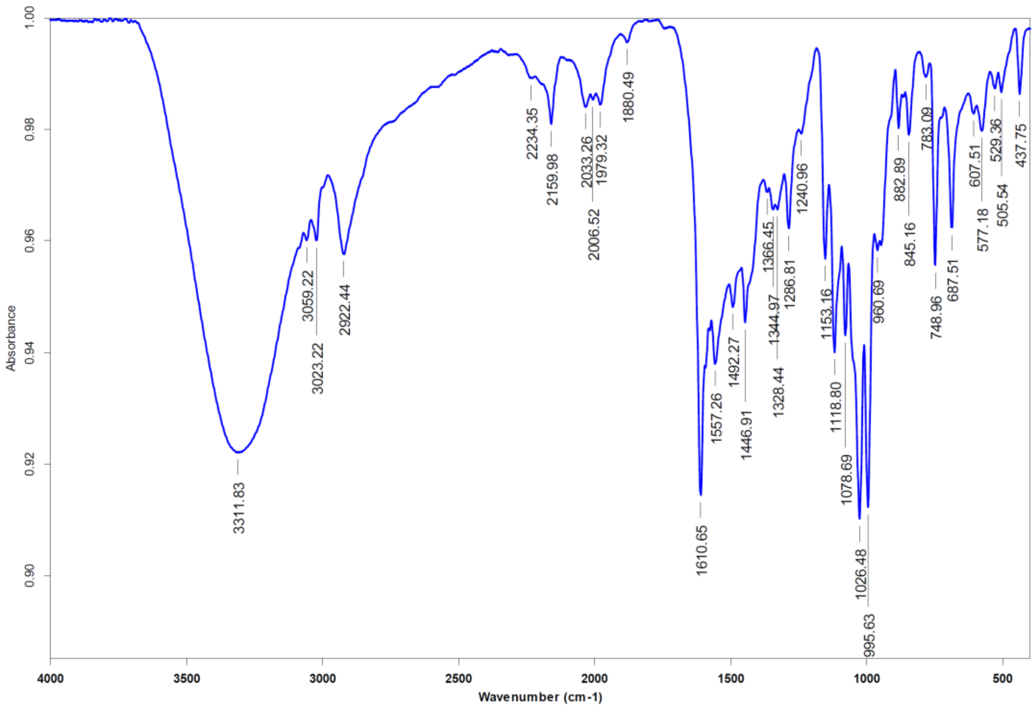

Figure S30. Infrared spectrum of association complex **4** (ATR)

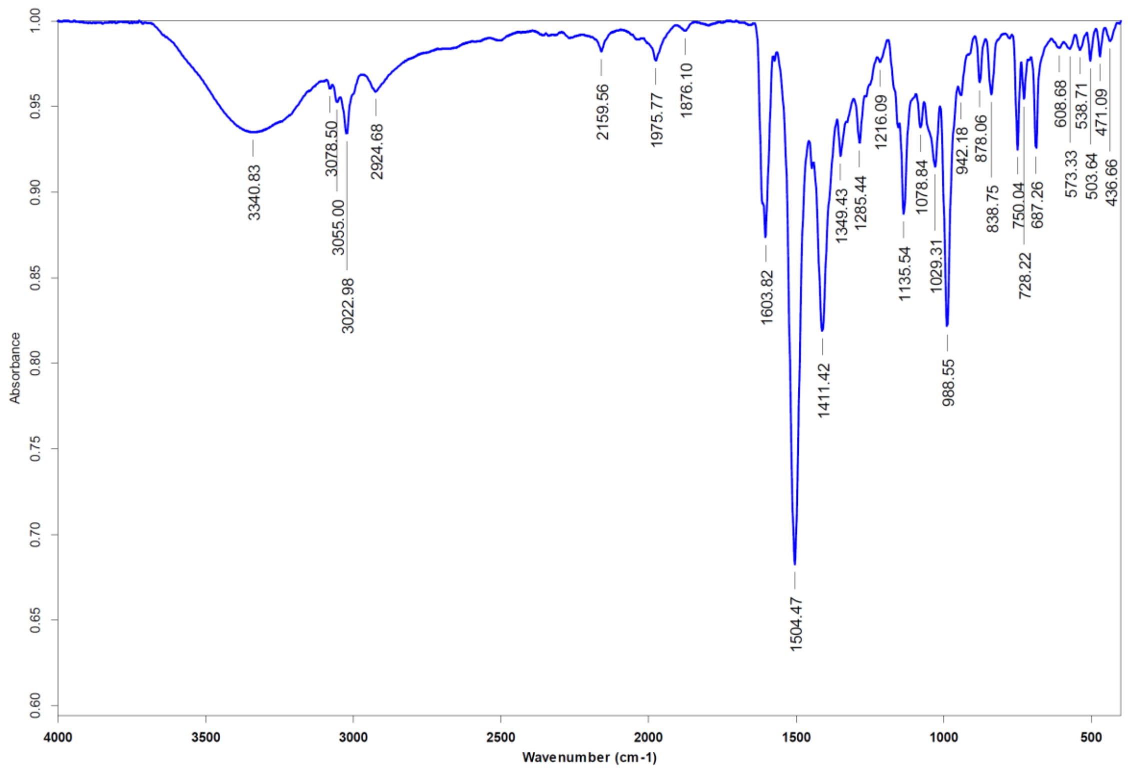

Figure S31. Infrared spectrum of association complex **5**(ATR)

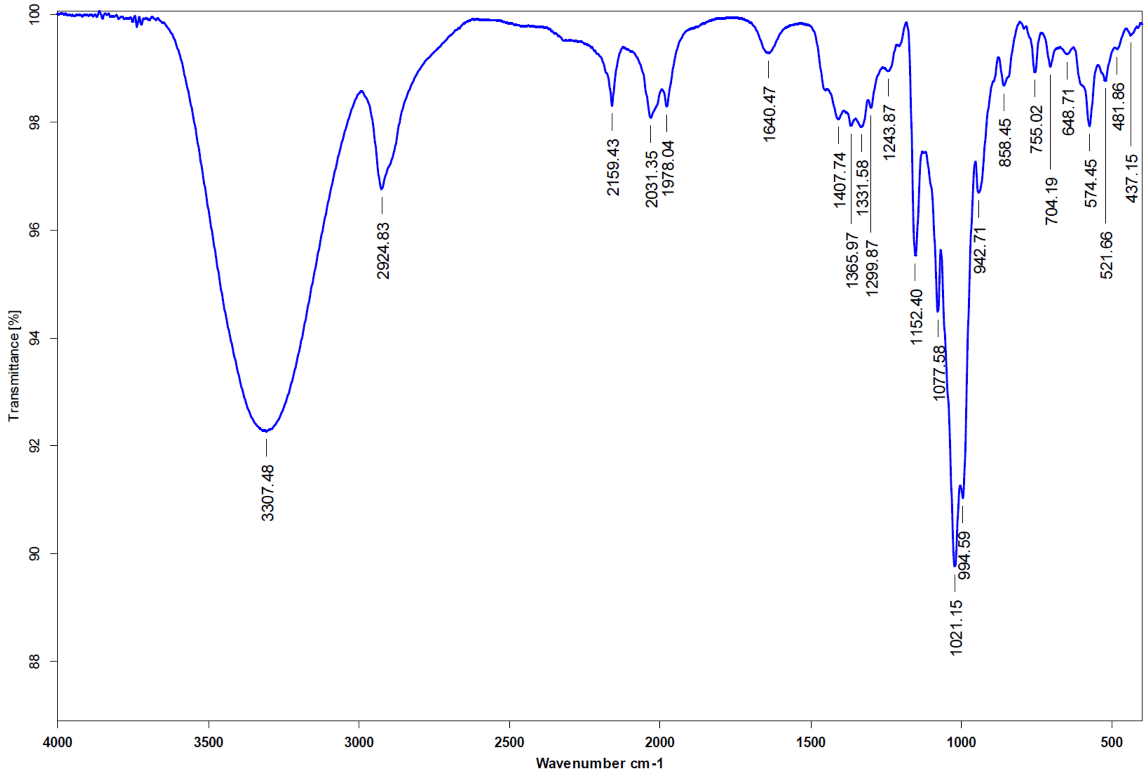

Figure S32. Infrared spectrum of β-cyclodextrin (ATR)

Ultraviolet-visible spectroscopy

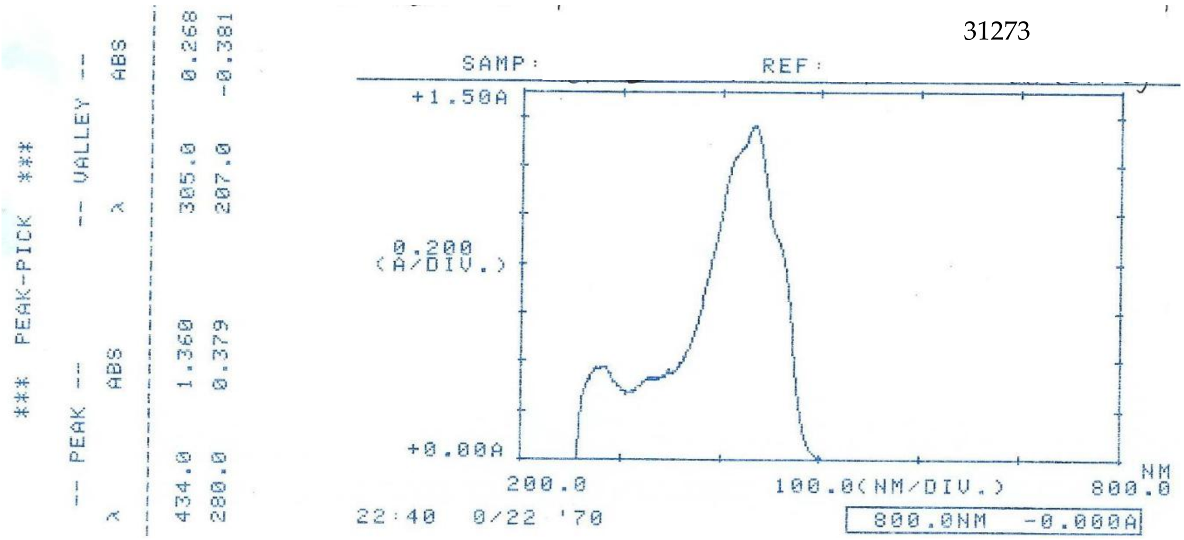

Figure S33. UV-Vis spectrum of compound **2** (DMSO)

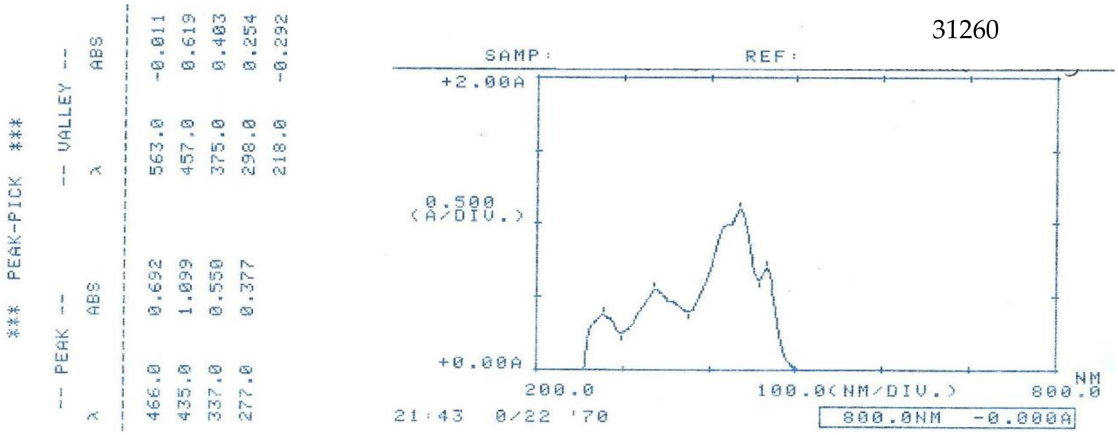

Figure S34. UV-Vis spectrum of compound **3** (DMSO)

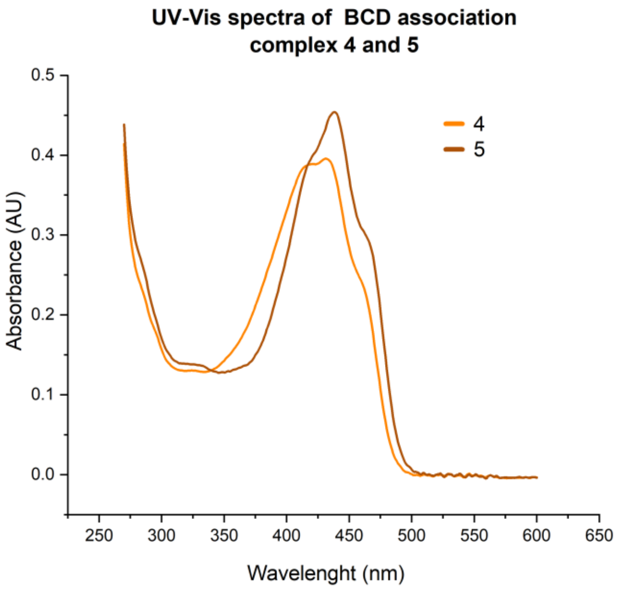

Figure S35. UV-Vis spectra of association complexes **4** and **5** in DMSO

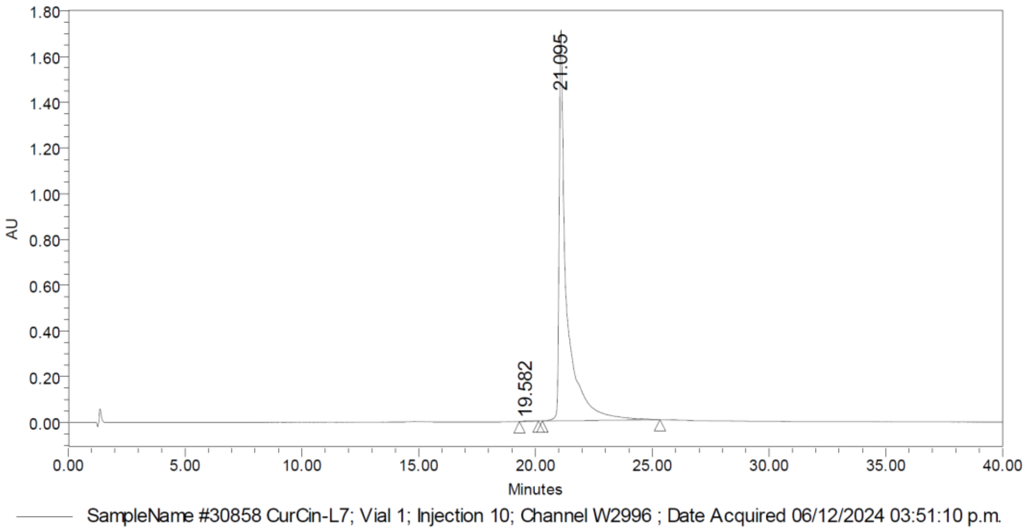

Peak Results

	Name	RT	Area	% Area	Int Type	Processed Channel Descr.	Width (sec)	Height (µV)
1		19.582	57734	0.15	bb	PDA 400.0 nm	49.000	2227
2		21.095	37808947	99.85	BB	PDA 400.0 nm	302.000	1711692

Figure S36. HPLC-UV chromatogram of compound **2**

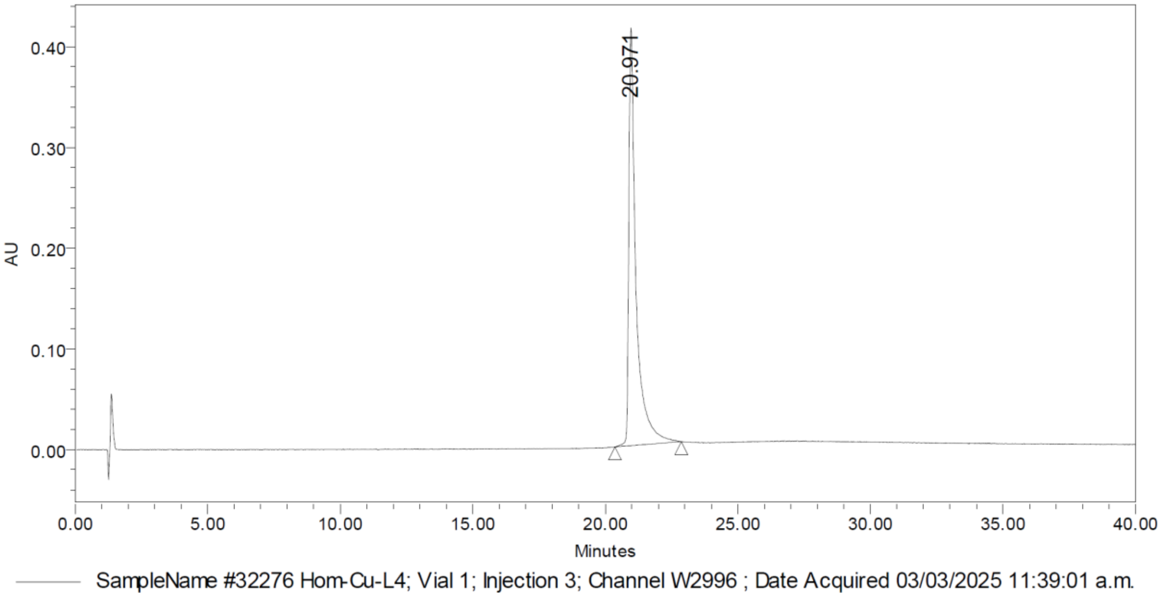

Peak Results

	Name	RT	Area	% Area	Int Type	Processed Channel Descr.	Width (sec)	Height (µV)
1		20.971	7838594	100.00	BB	PDA 426.0 nm	150.000	415328

Figure S37. HPLC-UV chromatogram of compound **2**

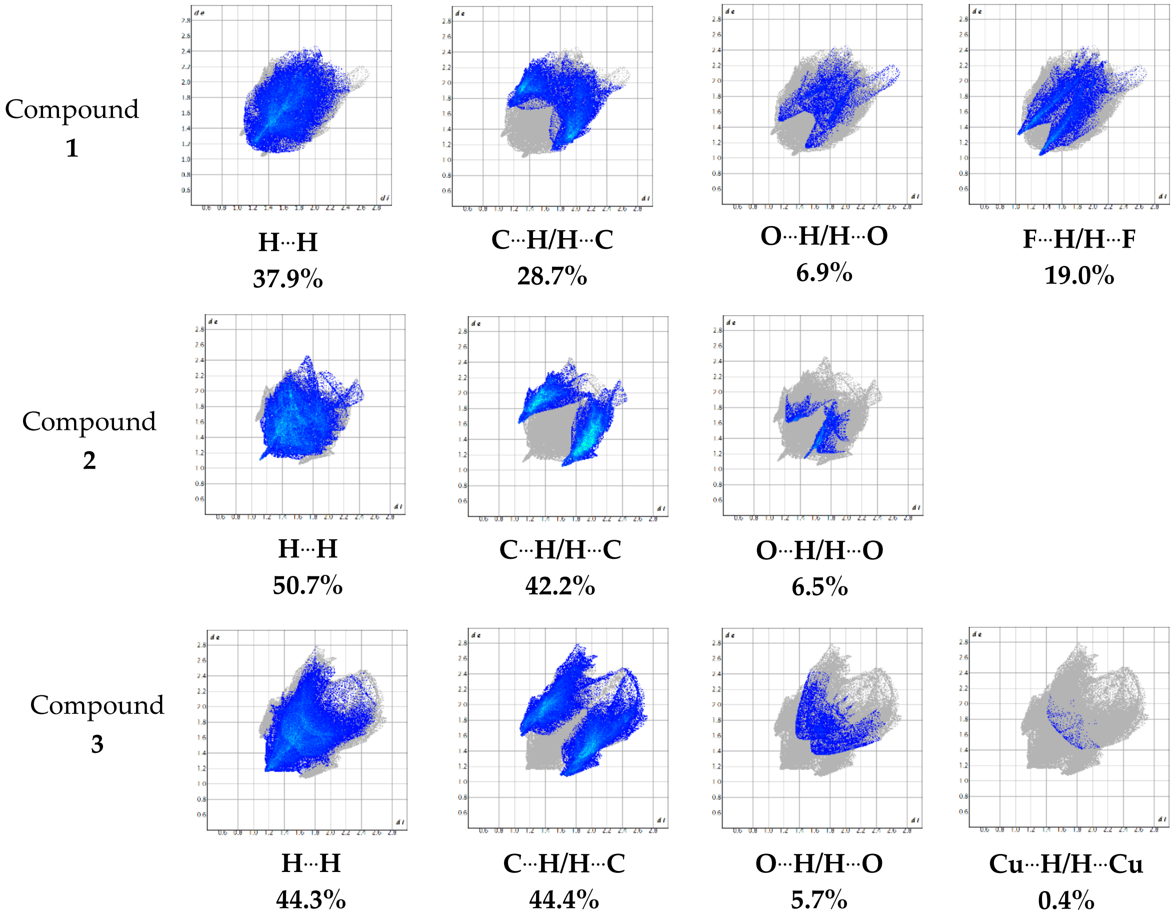

Figure S38. 2D-Fingerprint of Hirshfeld surface of compounds **1-3** and percentage of intermolecular interaction contributions in the crystal

T**able S1.** NOESY correlations of compound **2** in DMSO-d_6 and $CDCl_3$ (700 MHz)

**DMSO-d_6**		**$CDCl_3$**	
**δ (ppm)**	**Hydrogen name**	**δ (ppm)**	**Hydrogen name**
7.18-6.38	H_E-H_C	7.48-6.93	H_H-H_{EF}
7.45-6.38	H_D-H_C	7.48-6.18	H_H-H_C
7.45-6.09	H_D-H_A	7.44-5.71	H_D-H_A
7.45-7.11	H_D-H_F	6.93-6.18	H_{EF}-H_C
7.59-7.18	H_H-H_E	6.18-5.71	H_C-H_A
7.11-7.59	H_F-H_H		
6.38-6.09	H_C-H_A		

Table S2. Differences in ^{1}H and ^{13}C chemical shifts of free compound **2** and in complex ($\Delta\delta = \delta_{complex} - \delta_{free}$)

Name	Free compound **2**		Complex		Δδ (ppm)	
	^{1}H δ (ppm)	^{13}C δ (ppm)	^{1}H δ (ppm)	^{13}C δ (ppm)	^{1}H	^{13}C
A	6.092	101.344	6.091	101.355	-0.001	0.011
B		182.884		182.894	0	0.01
C	6.376	127.737	6.375	127.742	-0.001	0.005
D	7.447	141.004	7.447	141.016	0	0.012
E	7.176	127.355	7.175	127.357	-0.001	0.002
F	7.111	140.337	7.11	140.35	-0.001	0.013
G		136.098		136.101		0.003
H, H'	7.591	127.249	7.59	127.258	-0.001	0.009
I, I'	7.403	128.873	7.402	128.882	-0.001	0.009
J	7.341	129.058	7.341	129.072	0	0.014

Table S3. Differences in ^{1}H and ^{13}C chemical shifts of free BCD and in complex ($\Delta\delta = \delta_{complex} - \delta_{free}$)

	Free BCD		Complex		Δδ (ppm)	
Number	^{1}H δ (ppm)	^{13}C δ (ppm)	^{1}H δ (ppm)	^{13}C δ (ppm)	1H	13C
1	4.827	101.937	4.831	101.927	0.004	-0.01
2	3.291	81.534	3.299	81.531	0.008	-0.003
3	3.639	73.038	3.635	73.031	-0.004	-0.007
4	3.349	72.41	3.347	72.405	-0.002	-0.005
5	3.561	72.032	3.561	72.026	0	-0.006
6	3.639	59.909	3.635	59.9	-0.004	-0.009
C2-OH	5.715		5.715		0	
C3-OH	5.665		5.668		0.003	
C6-OH	4.437		4.437		0	

Antiproliferative activity

Assay: Sulforhodamine B
Concentration: 10 μM
Vehicle: DMSO

Table S4. Cellular growth inhibition (%)

	Cancer cell lines						
**Compound**	**U251**	**PC-3**	**K562**	**HCT-15**	**MCF-7**	**SKLU-1**	**COS7**
**2**	NA	11.78	NA	15.95	11.53	7.45	7.35
**4**	30.7	8.63	39.68	19.81	14.9	20.2	10.57
**3**	NA	1.95	NA	12.04	12.35	0.58	15.49
**5**	5.25	16.27	NA	21.5	21	12.88	12.27

NA: no activity

Cancer cell lines: U251= glioblastoma, PC-3= prostate, K562= leukemia, HCT-15= colon, MCF-7= breast, SKLU= lung COS-7: monkey kidney fibroblast

Table S5. Inhibition of yeast α-glucosidase. Determination of half-maximal inhibitory concentration (IC_{50})

**Compound**	**Concentration (µM)**	**D.O. 405 nm**	**Inhibition (%)**	**IC_{50} (µM)**
BCD (n=3)	Control	1.280±0.041		
	1	1.275±0.028	0.2±3.02	
	10	1.261±0.022	1.11±4.12	
	100	1.246±0.019	2.31±3.94	
**3** (n=3)	Control	1.280±0.041		
	1	1.314±0.041	-2.68±1.55	
	10	1.226±0.025	4.11±1.43	
	100	1.071±0.044**	16.19±3.18**	
**2** (n=3)	Control	1.305±0.046		
	10	1.205±0.051	7.61±2.80	
	17.78	1.084±0.067	16.93±3.75	
	31.62	0.894±0.105*	31.60±6.86*	45.63±13.12
	56.23	0.446±0.147**	66.28±10.17**	
	100	0.174±0.105**	86.93±7.53**	
**4** (n=3)	Control	1.280±0.041		
	10	1.127±0.028	11.60±3.88	
	17.78	1.074±0.030*	15.76±3.91*	
	31.62	0.880±0.053**	30.97±5.03**	51.11±11.05
	56.23	0.559±0.077**	55.95±6.79**	
	100	0.223±0.055**	82.43±4.50**	
**5** (n=4)	Control	1.299±0.052		
	10	1.137±0.087	12.72±4.74	
	17.78	0.950±0.087	27.11±5.33	36.27±12.85
	31.62	0.675±0.144**	48.69±10.23**	
	56.23	0.383±0.124**	71.23±9.12**	
	100	0.174±0.045**	86.81±3.00**	
Quercetin (n= 5)	Control	1.361±0.053		
	5.62	1.276±0.063	6.31±2.5	
	10	1.142±0.064*	16.22±2.95*	
	17.78	0.861±0.044**	36.87±1.16**	24.70±0.35
	31.62	0.556±0.019**	59.07±0.88**	
	56.23	0.376±0.016**	72.22±1.55**	

Values represent the mean of three to five independent experiments ± standard error of the mean. Data were examined by analysis of variance (ANOVA) followed by Dunnett's test to determine groups with significant differences. Values of $p \leq 0.05$ (*) and $p \leq 0.01$ (**) were considered significant differences from the control.

.

Table S6. Crystal data and structure refinement for **1**

Identification code	292EHR24	
Empirical formula	$C_{23} H_{19} B F_2 O_2$	
Formula weight	376.19	
Temperature	150(2) K	
Wavelength	0.71073 Å	
Crystal system	Triclinic	
Space group	P-1	
Unit cell dimensions	a = 9.1069(6) Å	α= 74.270(2)°.
	b = 10.0955(7) Å	β= 85.586(2)°.
	c = 10.7715(7) Å	γ = 85.457(2)°.
Volume	948.65(11) $Å^3$	
Z	2	
Density (calculated)	1.317 Mg/m^3	
Absorption coefficient	0.095 mm^{-1}	
F(000)	392	
Crystal size	0.291 x 0.239 x 0.178 mm^3	
Theta range for data collection	1.967 to 27.469°.	
Index ranges	-11<=h<=11, -13<=k<=13, -13<=l<=13	
Reflections collected	57505	
Independent reflections	4318 [R(int) = 0.0844]	
Completeness to theta = 25.242°	99.5 %	
Absorption correction	Integration	
Max. and min. transmission	0.9896 and 0.9806	
Refinement method	Full-matrix least-squares on F^2	
Data / restraints / parameters	4318 / 0 / 253	
Goodness-of-fit on F^2	1.080	
Final R indices [I>2sigma(I)]	R1 = 0.0490, wR2 = 0.1190	
R indices (all data)	R1 = 0.0610, wR2 = 0.1290	
Extinction coefficient	n/a	
Largest diff. peak and hole	0.313 and -0.220 e.$Å^{-3}$	

Table S7. Atomic coordinates (x 10^4) and equivalent isotropic displacement parameters ($Å^2$x 10^3) for **1**. U(eq) is defined as one third of the trace of the orthogonalized U^{ij} tensor.

	x	y	z	U(eq)
F(1)	2903(1)	3378(1)	6894(1)	38(1)
F(2)	3123(1)	3748(1)	8849(1)	46(1)
O(1)	5250(1)	3277(1)	7666(1)	32(1)
O(2)	3748(1)	5456(1)	7001(1)	31(1)
B(1)	3732(2)	3954(2)	7618(2)	30(1)
C(1)	9889(2)	-75(2)	8485(1)	29(1)
C(2)	9566(2)	1243(2)	7843(1)	29(1)
C(3)	8086(2)	1864(2)	7737(1)	29(1)
C(4)	7784(2)	3155(2)	7009(1)	28(1)
C(5)	6348(2)	3883(2)	6939(1)	26(1)
C(6)	6174(2)	5217(2)	6153(1)	27(1)
C(7)	4852(2)	5987(1)	6226(1)	25(1)
C(8)	4658(2)	7392(1)	5455(1)	27(1)
C(9)	3462(2)	8234(1)	5586(1)	26(1)
C(10)	3203(2)	9583(1)	4724(1)	26(1)
C(11)	1998(2)	10408(1)	4844(1)	26(1)
C(12)	11375(2)	-740(2)	8677(1)	28(1)
C(13)	12626(2)	13(2)	8484(2)	36(1)
C(14)	14002(2)	-659(2)	8733(2)	45(1)
C(15)	14149(2)	-2079(2)	9173(2)	45(1)
C(16)	12926(2)	-2835(2)	9356(2)	42(1)
C(17)	11542(2)	-2173(2)	9124(2)	34(1)
C(18)	1582(2)	11734(1)	3946(1)	25(1)
C(19)	2319(2)	12229(2)	2744(1)	30(1)
C(20)	1854(2)	13470(2)	1906(2)	36(1)
C(21)	642(2)	14244(2)	2258(2)	36(1)
C(22)	-98(2)	13773(2)	3446(2)	35(1)
C(23)	362(2)	12527(2)	4279(2)	31(1)

Table S8. Bond lengths [Å] and angles [°] for **1**.

		C(10)-H(10)	0.9500
F(1)-B(1)	1.387(2)	C(11)-C(18)	1.4649(19)
F(2)-B(1)	1.3652(19)	C(11)-H(11)	0.9500
O(1)-C(5)	1.3057(18)	C(12)-C(13)	1.391(2)
O(1)-B(1)	1.4908(19)	C(12)-C(17)	1.395(2)
O(2)-C(7)	1.3077(18)	C(13)-C(14)	1.386(2)
O(2)-B(1)	1.4822(19)	C(13)-H(13)	0.9500
C(1)-C(2)	1.344(2)	C(14)-C(15)	1.382(3)
C(1)-C(12)	1.466(2)	C(14)-H(14)	0.9500
C(1)-H(1)	0.9500	C(15)-C(16)	1.372(3)
C(2)-C(3)	1.439(2)	C(15)-H(15)	0.9500
C(2)-H(2)	0.9500	C(16)-C(17)	1.386(2)
C(3)-C(4)	1.346(2)	C(16)-H(16)	0.9500
C(3)-H(3)	0.9500	C(17)-H(17)	0.9500
C(4)-C(5)	1.4447(19)	C(18)-C(19)	1.395(2)
C(4)-H(4)	0.9500	C(18)-C(23)	1.3988(19)
C(5)-C(6)	1.387(2)	C(19)-C(20)	1.388(2)
C(6)-C(7)	1.3896(19)	C(19)-H(19)	0.9500
C(6)-H(6)	0.9500	C(20)-C(21)	1.390(2)
C(7)-C(8)	1.4408(19)	C(20)-H(20)	0.9500
C(8)-C(9)	1.350(2)	C(21)-C(22)	1.380(2)
C(8)-H(8)	0.9500	C(21)-H(21)	0.9500
C(9)-C(10)	1.4390(19)	C(22)-C(23)	1.387(2)
C(9)-H(9)	0.9500	C(22)-H(22)	0.9500
C(10)-C(11)	1.3440(19)	C(23)-H(23)	0.9500
C(5)-O(1)-B(1)	121.29(11)	C(2)-C(1)-H(1)	117.1
C(7)-O(2)-B(1)	121.49(11)	C(12)-C(1)-H(1)	117.1
F(2)-B(1)-F(1)	110.55(14)	C(1)-C(2)-C(3)	123.50(15)
F(2)-B(1)-O(2)	108.98(13)	C(1)-C(2)-H(2)	118.2
F(1)-B(1)-O(2)	108.24(12)	C(3)-C(2)-H(2)	118.2
F(2)-B(1)-O(1)	108.91(12)	C(4)-C(3)-C(2)	122.27(15)
F(1)-B(1)-O(1)	108.59(12)	C(4)-C(3)-H(3)	118.9
O(2)-B(1)-O(1)	111.56(13)	C(2)-C(3)-H(3)	118.9
C(2)-C(1)-C(12)	125.82(14)	C(3)-C(4)-C(5)	125.30(15)

Cont. Bond lengths [Å] and angles [°] for **1**.

C(3)-C(4)-H(4)	117.3	C(15)-C(14)-H(14)	119.7
C(5)-C(4)-H(4)	117.3	C(13)-C(14)-H(14)	119.7
O(1)-C(5)-C(6)	121.48(13)	C(16)-C(15)-C(14)	119.86(15)
O(1)-C(5)-C(4)	118.47(13)	C(16)-C(15)-H(15)	120.1
C(6)-C(5)-C(4)	120.00(14)	C(14)-C(15)-H(15)	120.1
C(5)-C(6)-C(7)	119.93(14)	C(15)-C(16)-C(17)	120.03(16)
C(5)-C(6)-H(6)	120.0	C(15)-C(16)-H(16)	120.0
C(7)-C(6)-H(6)	120.0	C(17)-C(16)-H(16)	120.0
O(2)-C(7)-C(6)	120.75(13)	C(16)-C(17)-C(12)	120.81(16)
O(2)-C(7)-C(8)	118.08(12)	C(16)-C(17)-H(17)	119.6
C(6)-C(7)-C(8)	121.18(13)	C(12)-C(17)-H(17)	119.6
C(9)-C(8)-C(7)	123.63(14)	C(19)-C(18)-C(23)	117.93(13)
C(9)-C(8)-H(8)	118.2	C(19)-C(18)-C(11)	122.93(13)
C(7)-C(8)-H(8)	118.2	C(23)-C(18)-C(11)	119.11(13)
C(8)-C(9)-C(10)	123.19(14)	C(20)-C(19)-C(18)	120.91(14)
C(8)-C(9)-H(9)	118.4	C(20)-C(19)-H(19)	119.5
C(10)-C(9)-H(9)	118.4	C(18)-C(19)-H(19)	119.5
C(11)-C(10)-C(9)	123.00(13)	C(19)-C(20)-C(21)	120.16(15)
C(11)-C(10)-H(10)	118.5	C(19)-C(20)-H(20)	119.9
C(9)-C(10)-H(10)	118.5	C(21)-C(20)-H(20)	119.9
C(10)-C(11)-C(18)	126.48(13)	C(22)-C(21)-C(20)	119.76(14)
C(10)-C(11)-H(11)	116.8	C(22)-C(21)-H(21)	120.1
C(18)-C(11)-H(11)	116.8	C(20)-C(21)-H(21)	120.1
C(13)-C(12)-C(17)	118.57(14)	C(21)-C(22)-C(23)	119.98(14)
C(13)-C(12)-C(1)	122.17(13)	C(21)-C(22)-H(22)	120.0
C(17)-C(12)-C(1)	119.19(14)	C(23)-C(22)-H(22)	120.0
C(14)-C(13)-C(12)	120.11(15)	C(22)-C(23)-C(18)	121.25(14)
C(14)-C(13)-H(13)	119.9	C(22)-C(23)-H(23)	119.4
C(12)-C(13)-H(13)	119.9	C(18)-C(23)-H(23)	119.4
C(15)-C(14)-C(13)	120.61(17)		

Table S9. Anisotropic displacement parameters ($Å^2 x 10^3$) for **1**. The anisotropic displacement factor exponent takes the form: $-2p^2[h^2 a^{*2}U^{11} + ... + 2 h k a^* b^* U^{12}]$

	U^{11}	U^{22}	U^{33}	U^{23}	U^{13}	U^{12}
F(1)	36(1)	30(1)	45(1)	-3(1)	-8(1)	1(1)
F(2)	49(1)	46(1)	32(1)	0(1)	6(1)	17(1)
O(1)	30(1)	26(1)	36(1)	-2(1)	-3(1)	8(1)
O(2)	28(1)	25(1)	38(1)	-4(1)	-1(1)	7(1)
B(1)	29(1)	26(1)	30(1)	-2(1)	0(1)	7(1)
C(1)	30(1)	28(1)	29(1)	-8(1)	-6(1)	4(1)
C(2)	29(1)	29(1)	28(1)	-8(1)	-5(1)	6(1)
C(3)	30(1)	30(1)	28(1)	-10(1)	-6(1)	6(1)
C(4)	27(1)	28(1)	30(1)	-10(1)	-6(1)	7(1)
C(5)	28(1)	27(1)	24(1)	-10(1)	-6(1)	5(1)
C(6)	25(1)	26(1)	30(1)	-8(1)	-4(1)	4(1)
C(7)	26(1)	24(1)	26(1)	-8(1)	-7(1)	3(1)
C(8)	25(1)	25(1)	29(1)	-7(1)	-6(1)	2(1)
C(9)	27(1)	22(1)	28(1)	-6(1)	-5(1)	1(1)
C(10)	26(1)	23(1)	28(1)	-6(1)	-4(1)	1(1)
C(11)	27(1)	24(1)	27(1)	-5(1)	-2(1)	1(1)
C(12)	31(1)	27(1)	23(1)	-6(1)	-5(1)	8(1)
C(13)	34(1)	31(1)	39(1)	-3(1)	-4(1)	4(1)
C(14)	29(1)	55(1)	43(1)	-2(1)	-2(1)	4(1)
C(15)	36(1)	56(1)	32(1)	1(1)	-1(1)	22(1)
C(16)	56(1)	31(1)	32(1)	-3(1)	-4(1)	19(1)
C(17)	42(1)	27(1)	33(1)	-9(1)	-7(1)	5(1)
C(18)	24(1)	21(1)	29(1)	-6(1)	-4(1)	0(1)
C(19)	30(1)	28(1)	31(1)	-9(1)	-2(1)	1(1)
C(20)	44(1)	32(1)	29(1)	-4(1)	-4(1)	-3(1)
C(21)	41(1)	24(1)	38(1)	-1(1)	-16(1)	2(1)
C(22)	28(1)	27(1)	50(1)	-9(1)	-8(1)	7(1)
C(23)	26(1)	27(1)	36(1)	-6(1)	1(1)	3(1)

Table S 10. Hydrogen coordinates (x 10^4) and isotropic displacement parameters ($Å^2$x 10^3) for **1**.

	x	y	z	U(eq)
H(1)	9083	-624	8847	35
H(2)	10356	1795	7436	35
H(3)	7296	1345	8198	35
H(4)	8578	3626	6499	34
H(6)	6958	5603	5566	33
H(8)	5412	7745	4819	32
H(9)	2757	7920	6281	31
H(10)	3916	9905	4038	31
H(11)	1352	10101	5586	32
H(13)	12537	990	8181	44
H(14)	14852	-138	8600	54
H(15)	15095	-2531	9348	54
H(16)	13027	-3813	9641	51
H(17)	10697	-2702	9271	41
H(19)	3151	11710	2496	36
H(20)	2365	13792	1089	43
H(21)	323	15093	1684	43
H(22)	-921	14303	3694	42
H(23)	-161	12207	5090	37

Table S11. Crystal data and structure refinement for **2**

Identification code	206EHR24	
Empirical formula	$C_{27} H_{28} O_3$	
Formula weight	400.49	
Temperature	150(2) K	
Wavelength	0.71073 Å	
Crystal system	Orthorhombic	
Space group	$Cmc2_1$	
Unit cell dimensions	a = 28.5258(9) Å	α= 90°.
	b = 9.7389(3) Å	β= 90°.
	c = 7.6957(2) Å	γ = 90°.
Volume	2137.94(11) $Å^3$	
Z	4	
Density (calculated)	1.244 Mg/m^3	
Absorption coefficient	0.080 mm^{-1}	
F(000)	856	
Crystal size	0.400 x 0.280 x 0.200 mm^3	
Theta range for data collection	2.210 to 27.450°.	
Index ranges	-35<=h<=36, -12<=k<=12, -9<=l<=9	
Reflections collected	19036	
Independent reflections	2447 [R(int) = 0.0276]	
Completeness to theta = 25.242°	98.9 %	
Absorption correction	Semi-empirical from equivalents	
Max. and min. transmission	0.7456 and 0.7062	
Refinement method	Full-matrix least-squares on F^2	
Data / restraints / parameters	2447 / 1 / 142	
Goodness-of-fit on F^2	1.221	
Final R indices [I>2sigma(I)]	R1 = 0.0415, wR2 = 0.0879	
R indices (all data)	R1 = 0.0456, wR2 = 0.0921	
Absolute structure parameter	-0.3(6)	
Largest diff. peak and hole	0.244 and -0.169 e.$Å^{-3}$	

Table S12. Atomic coordinates (x 10^4) and equivalent isotropic displacement parameters (Å^{2}x 10^3) for **2**. U(eq) is defined as one third of the trace of the orthogonalized U^{ij} tensor

	x	y	z	U(eq)
O(1)	4567(1)	2911(2)	2207(2)	30(1)
O(2)	5000	734(4)	5471(5)	64(1)
C(1)	2883(1)	3286(2)	3643(3)	22(1)
C(2)	3256(1)	3974(2)	4250(3)	22(1)
C(3)	3722(1)	3648(2)	3662(3)	23(1)
C(4)	4125(1)	4249(2)	4170(3)	23(1)
C(5)	4573(1)	3808(2)	3452(3)	22(1)
C(6)	5000	4321(3)	4085(5)	21(1)
C(7)	2388(1)	3485(2)	4092(3)	20(1)
C(8)	2060(1)	2523(2)	3497(3)	22(1)
C(9)	1587(1)	2661(2)	3884(3)	26(1)
C(10)	1434(1)	3773(3)	4860(3)	27(1)
C(11)	1753(1)	4742(2)	5442(3)	26(1)
C(12)	2227(1)	4600(2)	5069(3)	22(1)
C(13)	4601(1)	931(3)	6558(5)	50(1)
C(14)	4732(1)	2093(3)	7782(4)	36(1)

Table S 13. Bond lengths [Å] and angles [°] for **2**.

O(1)-C(5)	1.296(3)	C(7)-C(8)	1.402(3)
O(1)-H(1A)	0.88(5)	C(8)-C(9)	1.387(3)
O(2)-C(13)#1	1.425(4)	C(8)-H(8)	0.9500
O(2)-C(13)	1.425(4)	C(9)-C(10)	1.389(3)
C(1)-C(2)	1.342(3)	C(9)-H(9)	0.9500
C(1)-C(7)	1.465(3)	C(10)-C(11)	1.387(3)
C(1)-H(1)	0.9500	C(10)-H(10)	0.9500
C(2)-C(3)	1.440(3)	C(11)-C(12)	1.388(3)
C(2)-H(2)	0.9500	C(11)-H(11)	0.9500
C(3)-C(4)	1.346(3)	C(12)-H(12)	0.9500
C(3)-H(3)	0.9500	C(13)-C(14)	1.519(4)
C(4)-C(5)	1.459(3)	C(13)-H(13A)	0.9900
C(4)-H(4)	0.9500	C(13)-H(13B)	0.9900
C(5)-C(6)	1.403(3)	C(14)-C(14)#1	1.530(5)
C(6)-H(6)	0.9500	C(14)-H(14A)	0.9900
C(7)-C(12)	1.399(3)	C(14)-H(14B)	0.9900
C(5)-O(1)-H(1A)	108(3)	C(5)-C(6)-H(6)	119.8
C(13)#1-O(2)-C(13)	105.9(4)	C(5)#1-C(6)-H(6)	119.8
C(2)-C(1)-C(7)	128.0(2)	C(12)-C(7)-C(8)	118.3(2)
C(2)-C(1)-H(1)	116.0	C(12)-C(7)-C(1)	123.14(19)
C(7)-C(1)-H(1)	116.0	C(8)-C(7)-C(1)	118.60(19)
C(1)-C(2)-C(3)	120.9(2)	C(9)-C(8)-C(7)	121.0(2)
C(1)-C(2)-H(2)	119.6	C(9)-C(8)-H(8)	119.5
C(3)-C(2)-H(2)	119.6	C(7)-C(8)-H(8)	119.5
C(4)-C(3)-C(2)	126.9(2)	C(8)-C(9)-C(10)	119.8(2)
C(4)-C(3)-H(3)	116.6	C(8)-C(9)-H(9)	120.1
C(2)-C(3)-H(3)	116.6	C(10)-C(9)-H(9)	120.1
C(3)-C(4)-C(5)	120.7(2)	C(11)-C(10)-C(9)	119.9(2)
C(3)-C(4)-H(4)	119.7	C(11)-C(10)-H(10)	120.0
C(5)-C(4)-H(4)	119.7	C(9)-C(10)-H(10)	120.0
O(1)-C(5)-C(6)	120.6(2)	C(10)-C(11)-C(12)	120.3(2)
O(1)-C(5)-C(4)	117.7(2)	C(10)-C(11)-H(11)	119.8
C(6)-C(5)-C(4)	121.7(2)	C(12)-C(11)-H(11)	119.8
C(5)-C(6)-C(5)#1	120.4(3)	C(11)-C(12)-C(7)	120.6(2)

Cont. Bond lengths [Å] and angles [°] for **2**.

C(11)-C(12)-H(12)	119.7	H(13A)-C(13)-H(13B)	108.8
C(7)-C(12)-H(12)	119.7	C(13)-C(14)-C(14)#1	104.20(18)
O(2)-C(13)-C(14)	105.6(3)	C(13)-C(14)-H(14A)	110.9
O(2)-C(13)-H(13A)	110.6	C(14)#1-C(14)-H(14A)	110.9
C(14)-C(13)-H(13A)	110.6	C(13)-C(14)-H(14B)	110.9
O(2)-C(13)-H(13B)	110.6	C(14)#1-C(14)-H(14B)	110.9
C(14)-C(13)-H(13B)	110.6	H(14A)-C(14)-H(14B)	108.9

Symmetry transformations used to generate equivalent atoms:

#1 -x+1,y,z

Table S14. Anisotropic displacement parameters ($Å^2$x 10^3) for **2**. The anisotropic displacement factor exponent takes the form: $-2p^2[h^2 a^{*2}U^{11} + ... + 2 h k a^* b^* U^{12}]$

	U^{11}	U^{22}	U^{33}	U^{23}	U^{13}	U^{12}
O(1)	19(1)	39(1)	31(1)	-13(1)	1(1)	0(1)
O(2)	91(3)	57(2)	44(2)	-18(2)	0	0
C(1)	20(1)	24(1)	20(1)	0(1)	2(1)	2(1)
C(2)	20(1)	24(1)	23(1)	2(1)	2(1)	1(1)
C(3)	21(1)	26(1)	21(1)	1(1)	0(1)	1(1)
C(4)	19(1)	26(1)	23(1)	0(1)	1(1)	3(1)
C(5)	20(1)	25(1)	21(1)	2(1)	0(1)	1(1)
C(6)	18(2)	22(2)	24(2)	-1(1)	0	0
C(7)	18(1)	22(1)	18(1)	2(1)	1(1)	-1(1)
C(8)	22(1)	22(1)	24(1)	-1(1)	-2(1)	0(1)
C(9)	20(1)	27(1)	30(1)	1(1)	-2(1)	-5(1)
C(10)	18(1)	33(1)	29(1)	3(1)	2(1)	1(1)
C(11)	26(1)	26(1)	26(1)	-3(1)	4(1)	4(1)
C(12)	22(1)	23(1)	23(1)	-1(1)	-2(1)	-3(1)
C(13)	56(2)	42(2)	51(2)	-1(2)	-12(2)	-14(2)
C(14)	31(1)	39(2)	38(1)	-3(1)	2(1)	-2(1)

Table S15. Hydrogen coordinates (x 10^4) and isotropic displacement parameters ($Å^2$x 10^{3}) for **2**.

	x	y	z	U(eq)
H(1A)	4858(18)	2780(50)	1870(70)	36
H(1)	2947	2583	2821	26
H(2)	3213	4686	5077	27
H(3)	3749	2932	2829	27
H(4)	4116	4968	5004	27
H(6)	5000	5018	4948	26
H(8)	2162	1766	2819	27
H(9)	1369	1996	3482	31
H(10)	1110	3869	5128	32
H(11)	1648	5507	6100	31
H(12)	2443	5267	5480	27
H(13A)	4530	84	7219	59
H(13B)	4323	1181	5856	59
H(14A)	4608	1922	8964	43
H(14B)	4608	2981	7355	43

Table S16. Hydrogen bonds for 1 [Å and °].

D-H...A	d(D-H)	d(H...A)	d(D...A)	<(DHA)
O(1)-H(1A)...O(1)#1	0.88(5)	1.67(5)	2.471(3)	151(5)

Symmetry transformations used to generate equivalent atoms:

#1 -x+1,y,z

Table S17. Crystal data and structure refinement for 3.

Identification code	201EHR24	
Empirical formula	C46 H38 Cu O4	
Formula weight	718.30	
Temperature	100(2) K	
Wavelength	0.71073 Å	
Crystal system	Monoclinic	
Space group	C2/c	
Unit cell dimensions	a = 41.1114(11) Å	α= 90°.
	b = 5.56190(10) Å	β= 100.2810(10)°.
	c = 16.1765(4) Å	γ = 90°.
Volume	3639.49(15) $Å^3$	
Z	4	
Density (calculated)	1.311 Mg/m^3	
Absorption coefficient	0.644 mm^{-1}	
F(000)	1500	
Crystal size	0.346 x 0.106 x 0.089 mm^3	
Theta range for data collection	3.529 to 27.513°.	
Index ranges	-52<=h<=52, -7<=k<=7, -20<=l<=21	
Reflections collected	51317	
Independent reflections	4157 [R(int) = 0.0236]	
Completeness to theta = 25.242°	99.2 %	
Absorption correction	Semi-empirical from equivalents	
Max. and min. transmission	0.7456 and 0.7091	
Refinement method	Full-matrix least-squares on F^2	
Data / restraints / parameters	4157 / 0 / 232	
Goodness-of-fit on F^2	1.113	
Final R indices [I>2sigma(I)]	R1 = 0.0311, wR2 = 0.0747	
R indices (all data)	R1 = 0.0352, wR2 = 0.0784	
Extinction coefficient	n/a	
Largest diff. peak and hole	0.373 and -0.448 e.$Å^{-3}$	

Table S18. Atomic coordinates (x 10^4) and equivalent isotropic displacement parameters ($Å^2$x 10^3) for **3**. U(eq) is defined as one third of the trace of the orthogonalized U^{ij} tensor.

	x	y	z	U(eq)
Cu(1)	5000	10000	5000	17(1)
O(1)	5203(1)	7251(2)	4597(1)	21(1)
O(2)	4637(1)	9946(2)	4078(1)	21(1)
C(1)	3481(1)	12021(3)	2643(1)	20(1)
C(2)	3696(1)	10251(3)	2565(1)	23(1)
C(3)	4024(1)	10124(3)	3061(1)	22(1)
C(4)	4237(1)	8322(3)	3011(1)	24(1)
C(5)	4563(1)	8204(3)	3564(1)	20(1)
C(6)	4762(1)	6192(3)	3496(1)	23(1)
C(7)	5072(1)	5840(3)	4010(1)	19(1)
C(8)	5266(1)	3691(3)	3873(1)	21(1)
C(9)	5554(1)	3209(3)	4386(1)	20(1)
C(10)	5767(1)	1190(3)	4317(1)	20(1)
C(11)	6052(1)	907(3)	4858(1)	18(1)
C(12)	3143(1)	12248(3)	2169(1)	17(1)
C(13)	3006(1)	10574(3)	1556(1)	19(1)
C(14)	2688(1)	10880(3)	1110(1)	20(1)
C(15)	2500(1)	12847(3)	1267(1)	20(1)
C(16)	2630(1)	14513(3)	1872(1)	21(1)
C(17)	2950(1)	14212(3)	2317(1)	19(1)
C(18)	6300(1)	-977(3)	4846(1)	16(1)
C(19)	6243(1)	-2994(3)	4324(1)	18(1)
C(20)	6489(1)	-4690(3)	4308(1)	21(1)
C(21)	6798(1)	-4422(3)	4823(1)	21(1)
C(22)	6854(1)	-2469(3)	5359(1)	21(1)
C(23)	6609(1)	-761(3)	5370(1)	19(1)

Table S19. Bond lengths [Å] and angles [°] for **3**.

Cu(1)-O(1)#1	1.9104(10)	C(11)-C(18)	1.4641(19)
Cu(1)-O(1)	1.9104(10)	C(11)-H(11)	0.9500
Cu(1)-O(2)	1.9136(10)	C(12)-C(17)	1.396(2)
Cu(1)-O(2)#1	1.9136(10)	C(12)-C(13)	1.403(2)
O(1)-C(7)	1.2757(18)	C(13)-C(14)	1.386(2)
O(2)-C(5)	1.2776(19)	C(13)-H(13)	0.9500
C(1)-C(2)	1.343(2)	C(14)-C(15)	1.389(2)
C(1)-C(12)	1.4676(19)	C(14)-H(14)	0.9500
C(1)-H(1)	0.9500	C(15)-C(16)	1.384(2)
C(2)-C(3)	1.443(2)	C(15)-H(15)	0.9500
C(2)-H(2)	0.9500	C(16)-C(17)	1.391(2)
C(3)-C(4)	1.343(2)	C(16)-H(16)	0.9500
C(3)-H(3)	0.9500	C(17)-H(17)	0.9500
C(4)-C(5)	1.474(2)	C(18)-C(19)	1.399(2)
C(4)-H(4)	0.9500	C(18)-C(23)	1.4002(19)
C(5)-C(6)	1.402(2)	C(19)-C(20)	1.386(2)
C(6)-C(7)	1.404(2)	C(19)-H(19)	0.9500
C(6)-H(6)	0.9500	C(20)-C(21)	1.395(2)
C(7)-C(8)	1.475(2)	C(20)-H(20)	0.9500
C(8)-C(9)	1.345(2)	C(21)-C(22)	1.383(2)
C(8)-H(8)	0.9500	C(21)-H(21)	0.9500
C(9)-C(10)	1.443(2)	C(22)-C(23)	1.388(2)
C(9)-H(9)	0.9500	C(22)-H(22)	0.9500
C(10)-C(11)	1.340(2)	C(23)-H(23)	0.9500
C(10)-H(10)	0.9500		
O(1)#1-Cu(1)-O(1)	180.0	C(2)-C(1)-H(1)	116.9
O(1)#1-Cu(1)-O(2)	87.18(4)	C(12)-C(1)-H(1)	116.9
O(1)-Cu(1)-O(2)	92.82(4)	C(1)-C(2)-C(3)	123.30(15)
O(1)#1-Cu(1)-O(2)#1	92.82(4)	C(1)-C(2)-H(2)	118.4
O(1)-Cu(1)-O(2)#1	87.18(4)	C(3)-C(2)-H(2)	118.4
O(2)-Cu(1)-O(2)#1	180.0	C(4)-C(3)-C(2)	123.97(15)
C(7)-O(1)-Cu(1)	126.47(9)	C(4)-C(3)-H(3)	118.0
C(5)-O(2)-Cu(1)	125.45(9)	C(2)-C(3)-H(3)	118.0
C(2)-C(1)-C(12)	126.23(14)	C(3)-C(4)-C(5)	121.95(15)

Cont. Bond lengths [Å] and angles [°] for **3**.

C(3)-C(4)-H(4)	119.0	C(13)-C(14)-H(14)	119.8
C(5)-C(4)-H(4)	119.0	C(15)-C(14)-H(14)	119.8
O(2)-C(5)-C(6)	125.67(13)	C(16)-C(15)-C(14)	119.92(13)
O(2)-C(5)-C(4)	116.41(13)	C(16)-C(15)-H(15)	120.0
C(6)-C(5)-C(4)	117.91(14)	C(14)-C(15)-H(15)	120.0
C(5)-C(6)-C(7)	123.02(14)	C(15)-C(16)-C(17)	119.73(14)
C(5)-C(6)-H(6)	118.5	C(15)-C(16)-H(16)	120.1
C(7)-C(6)-H(6)	118.5	C(17)-C(16)-H(16)	120.1
O(1)-C(7)-C(6)	124.96(14)	C(16)-C(17)-C(12)	121.25(14)
O(1)-C(7)-C(8)	116.22(12)	C(16)-C(17)-H(17)	119.4
C(6)-C(7)-C(8)	118.82(14)	C(12)-C(17)-H(17)	119.4
C(9)-C(8)-C(7)	120.60(14)	C(19)-C(18)-C(23)	118.00(13)
C(9)-C(8)-H(8)	119.7	C(19)-C(18)-C(11)	122.72(13)
C(7)-C(8)-H(8)	119.7	C(23)-C(18)-C(11)	119.28(13)
C(8)-C(9)-C(10)	126.01(14)	C(20)-C(19)-C(18)	120.90(13)
C(8)-C(9)-H(9)	117.0	C(20)-C(19)-H(19)	119.6
C(10)-C(9)-H(9)	117.0	C(18)-C(19)-H(19)	119.6
C(11)-C(10)-C(9)	120.91(14)	C(19)-C(20)-C(21)	120.27(14)
C(11)-C(10)-H(10)	119.5	C(19)-C(20)-H(20)	119.9
C(9)-C(10)-H(10)	119.5	C(21)-C(20)-H(20)	119.9
C(10)-C(11)-C(18)	127.19(14)	C(22)-C(21)-C(20)	119.45(14)
C(10)-C(11)-H(11)	116.4	C(22)-C(21)-H(21)	120.3
C(18)-C(11)-H(11)	116.4	C(20)-C(21)-H(21)	120.3
C(17)-C(12)-C(13)	118.16(13)	C(21)-C(22)-C(23)	120.28(14)
C(17)-C(12)-C(1)	119.47(13)	C(21)-C(22)-H(22)	119.9
C(13)-C(12)-C(1)	122.35(13)	C(23)-C(22)-H(22)	119.9
C(14)-C(13)-C(12)	120.60(14)	C(22)-C(23)-C(18)	121.08(14)
C(14)-C(13)-H(13)	119.7	C(22)-C(23)-H(23)	119.5
C(12)-C(13)-H(13)	119.7	C(18)-C(23)-H(23)	119.5
C(13)-C(14)-C(15)	120.33(14)		

Symmetry transformations used to generate equivalent atoms:

#1 -x+1,-y+2,-z+1

Table S20. Anisotropic displacement parameters ($Å^2$x 10^3) for **3**. The anisotropic displacement factor exponent takes the form: $-2\pi^2[h^2 a^{*2}U^{11} + ... + 2 h k a^* b^* U^{12}]$

	U^{11}	U^{22}	U^{33}	U^{23}	U^{13}	U^{12}
Cu(1)	12(1)	17(1)	21(1)	0(1)	-1(1)	4(1)
O(1)	14(1)	19(1)	28(1)	-2(1)	-1(1)	4(1)
O(2)	15(1)	21(1)	23(1)	0(1)	-3(1)	5(1)
C(1)	17(1)	25(1)	16(1)	1(1)	1(1)	2(1)
C(2)	19(1)	31(1)	16(1)	-1(1)	-1(1)	6(1)
C(3)	18(1)	31(1)	17(1)	1(1)	0(1)	5(1)
C(4)	19(1)	34(1)	18(1)	-3(1)	-2(1)	6(1)
C(5)	15(1)	27(1)	17(1)	3(1)	1(1)	4(1)
C(6)	19(1)	28(1)	21(1)	-3(1)	-1(1)	6(1)
C(7)	16(1)	20(1)	21(1)	2(1)	3(1)	2(1)
C(8)	18(1)	22(1)	23(1)	-1(1)	4(1)	3(1)
C(9)	16(1)	19(1)	24(1)	1(1)	6(1)	2(1)
C(10)	17(1)	18(1)	24(1)	-1(1)	5(1)	2(1)
C(11)	18(1)	16(1)	21(1)	0(1)	6(1)	2(1)
C(12)	15(1)	20(1)	15(1)	3(1)	2(1)	2(1)
C(13)	17(1)	18(1)	21(1)	0(1)	3(1)	2(1)
C(14)	19(1)	20(1)	20(1)	0(1)	0(1)	-3(1)
C(15)	13(1)	25(1)	22(1)	4(1)	0(1)	1(1)
C(16)	18(1)	21(1)	23(1)	2(1)	3(1)	6(1)
C(17)	18(1)	19(1)	18(1)	-1(1)	2(1)	1(1)
C(18)	15(1)	16(1)	18(1)	3(1)	5(1)	2(1)
C(19)	16(1)	20(1)	20(1)	1(1)	3(1)	0(1)
C(20)	22(1)	17(1)	23(1)	0(1)	7(1)	0(1)
C(21)	19(1)	19(1)	26(1)	5(1)	7(1)	6(1)
C(22)	15(1)	25(1)	22(1)	3(1)	1(1)	3(1)
C(23)	19(1)	19(1)	19(1)	0(1)	2(1)	1(1)

Table S21. Hydrogen coordinates (x 10^4) and isotropic displacement parameters ($Å^2$x 10^3) for **3**.

	x	y	z	U(eq)
H(1)	3555	13244	3042	24
H(2)	3628	9021	2164	27
H(3)	4094	11391	3446	26
H(4)	4176	7088	2607	29
H(6)	4684	5014	3083	28
H(8)	5187	2639	3419	25
H(9)	5622	4298	4836	24
H(10)	5705	45	3881	23
H(11)	6098	2056	5298	22
H(13)	3132	9218	1447	22
H(14)	2599	9738	694	24
H(15)	2282	13050	960	24
H(16)	2502	15857	1983	25
H(17)	3039	15364	2730	23
H(19)	6034	-3204	3975	22
H(20)	6448	-6040	3944	25
H(21)	6967	-5570	4806	25
H(22)	7062	-2296	5721	25
H(23)	6651	576	5739	23

TGA and DSC thermograms of compounds 2-5

The TGA thermograms of **2**, **4**, and dry BCD are shown in Figure 1a. TGA of compound **2** showed a unique loss of mass (67.8%), while **4** has three losses of mass of 6.7, 40.8, and 24.8% related to loss of water and decomposition. Dry BCD has a two-loss of mass of 7.0 and 72.2% attributed to loss of water and decomposition. These results exhibit that the loss of water in 4 is less than in dry BCD, despite the water treatment of **4** after the mechanochemical procedure, therefore suggesting a loss of water due to the inclusion formation. This behaviour has been reported in inclusion complexes of essential oils with BCD [1].

The DSC curves of **2**, **4**, and dry BCD are shown in Figure 1b. DSC of **2** exhibits an endothermic peak at 192.2 °C, which corresponds to the melting point; in addition, an exothermic process at 267.6 °C is observed. In the DSC of **4**, the melting point of **2** disappeared, and four main endothermic processes at 119.4, 186.9, 313.2, and 401.1 °C are observed as a result of dehydration, partial fusion, and decomposition. Some authors attribute the disappearance of the melting point of the guest to inclusion formation [2,3].

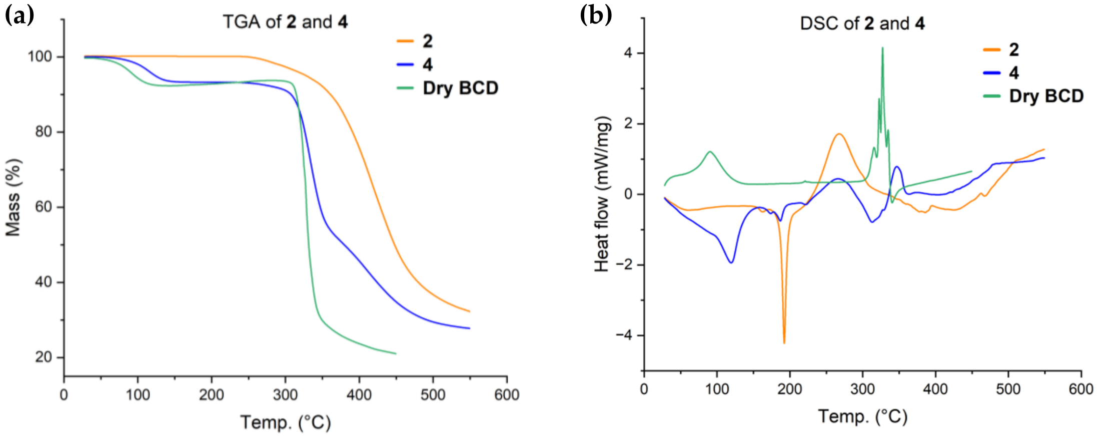

Graphic S1. TGA and DSC thermograms of compounds **2**, **4** and dry BCD

In the case of compounds **3** and **5**, the TGA and DSC thermograms are shown in Figure 2. TGA of **3** showed a first loss of water (4.7%) and a second loss of mass (42.8%), while TGA of 5 showed a first loss of water (5.7%) followed by two successive losses of mass of 32.6 and 24.9%. We can observe that the loss of water of **5** is less than dry BCD (7.0%) and is closer to the water loss of **2**, suggesting the water displacement of BCD favours the inclusion formation.

DSC curves of **3** (Figure 2b) show two endothermic peaks at 272.4 °C corresponding to the melting point and at 414.4 °C to the decomposition process. In the DSC of **5**, the melting point of 3 disappeared and two endothermic broad peaks at 112.6 and 405.1 °C are observed. Exothermic peaks were detected at 266.4 and 343.5 °C. These results suggest the formation of an inclusion complex.

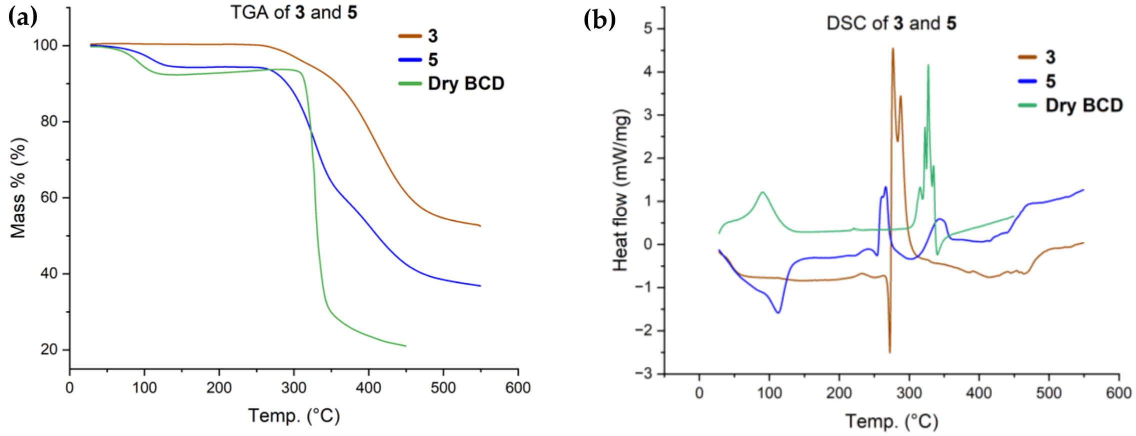

Graphic S2. TGA and DSC thermograms of compounds **3**, **5** and dry BCD

In general terms, the thermal profile of both **3** and **5** changes, indicating the formation of a new phase

References

1. 1. Abarca, R.L.; Rodríguez, F.J.; Guarda, A.; Galotto, M.J.; Bruna, J.E. Characterization of Beta-Cyclodextrin Inclusion Complexes Containing an Essential Oil Component. *Food Chem.* **2016**, *196*, 968–975. https://doi.org/10.1016/j.foodchem.2015.10.023.
2. Kim, J.-S. Study of Flavonoid/Hydroxypropyl-β-Cyclodextrin Inclusion Complexes by UV-Vis, FT-IR, DSC, and X-Ray Diffraction Analysis. *Prev. Nutr. Food Sci.* **2020**, *25*, 449–456. https://doi.org/10.3746/pnf.2020.25.4.449.
3. Zhang, W.; Zhou, T.-P.; Zou, W.; Wang, Y.; Wang, K.; Yang, Y.; Liu, C.; Tu, Z.; Liu, Q.; Yuan, Y. Formation of β-Cyclodextrin Inclusion Complexes with a Series of Structurally Related Parabens: Preparation, Physicochemical Characterization and Antifungal Properties. *Carbohydr. Polym. Technol. Appl.* **2025**, *11*, 100924. https://doi.org/10.1016/j.carpta.2025.100924.
